# CMOT: Cross-Modality Optimal Transport for multimodal inference

**DOI:** 10.1186/s13059-023-02989-8

**Published:** 2023-07-11

**Authors:** Sayali Anil Alatkar, Daifeng Wang

**Affiliations:** 1 Waisman Center, University of Wisconsin-Madison, Madison, WI 53705 USA; 2 Department of Computer Sciences, University of Wisconsin-Madison, Madison, WI 53076 USA; 3 Department of Biostatistics and Medical Informatics, University of Wisconsin-Madison, Madison, WI 53076 USA

**Keywords:** Optimal transport, Multimodal data alignment, Cross-modal inference, Single-cell multi-modality, Probabilistic coupling, Weighted nearest neighbor

## Abstract

Multimodal measurements of single-cell sequencing technologies facilitate a comprehensive understanding of specific cellular and molecular mechanisms. However, simultaneous profiling of multiple modalities of single cells is challenging, and data integration remains elusive due to missing modalities and cell–cell correspondences. To address this, we developed a computational approach, Cross-Modality Optimal Transport (CMOT), which aligns cells within available multi-modal data (source) onto a common latent space and infers missing modalities for cells from another modality (target) of mapped source cells. CMOT outperforms existing methods in various applications from developing brain, cancers to immunology, and provides biological interpretations improving cell-type or cancer classifications.

**Supplementary Information:**

The online version contains supplementary material available at 10.1186/s13059-023-02989-8.

## Background

Single-cell sequencing technologies can measure different characteristics of single cells across multi-omics such as genomics, transcriptomics, epigenomics, and proteomics. Such high-resolution measurements have enabled exploring individual cells to reveal cellular and molecular mechanisms and study cell-to-cell functional variations. For example, sci-CAR, 10xMultiome, and scCAT-seq measure single-cell gene expression and chromatin accessibility [1–3], and CITE-seq measures gene and protein expression of single cells [4, 5]. However, simultaneous profiling of such multi-omics and additional modalities continues to be a challenging task especially because of high sequencing costs, low recovery of individual cells, and sparse and noisy data [6]. Owing to these challenges, single-cell multimodal data generation may not always be feasible. This leads to the question of how we can use available multi-modalities to infer missing modalities.

Several prior works have tackled modality inference. Seurat [7, 8], infers the missing modality of a cell by weighting nearest neighboring cells with multimodalities available. MOFA + [9] uses Bayesian factor analysis to identify a lower dimensional representation of the data to infer the missing modality. However, they only work with multimodal data that must come from the same cells (i.e., fully corresponding). Alignment-based methods like non-linear manifold alignment [10] have been shown to align multimodalities with partial cell-to-cell correspondence information but have not been extended to cross-modality inference. Machine learning has also emerged to help modality inference. For instance, TotalVI [5] builds a variational autoencoder that infers missing protein profiles from gene expression using CITE-seq data. Polarbear [11] also uses autoencoders; however, trains on both single and multi-modal data to infer each modality. However, such autoencoder-based approaches are unsupervised that learn the latent embeddings that likely lack biological interpretability and lack a mechanism to introduce prior knowledge about underlying data distribution [12]. Moreover, training autoencoders typically requires considerable amounts of data and time with intensive hyperparameter tuning.

Optimal Transport (OT) is an efficient approach that uses prior knowledge about data distribution to find an optimal mapping between the distributions [13]. OT can also work on small datasets with limited parameters. Recently, OT has been applied to single-cell multiomics data for various applications [14–17]. Schiebinger et al. [14] used OT to model the developmental trajectory of single-cell gene expression through unbalanced optimal transport. Single-cell integrative analysis frameworks like SCOT [15], SCOTv2 [16], and Pamona [17] further extended the original OT problem for multi-omics data alignment. Another work [18] used OT with an additional entropic regularization term to improve the unsupervised clustering of single-cell data to understand cell types and cellular states better. However, OT has not yet been applied for cross-modality inference. Thus, we propose that integrating OT with multimodal data alignment can work for cross-modality inference and address the above limitations of prior works.

Particularly, we developed CMOT (Cross-Modality Optimal Transport), a computational approach to infer missing modalities of single cells. CMOT first aligns the cells with multimodal data (source) if the cells do not have complete correspondence, and then applies OT to map the cells from single modality (target) to the source cells via shared modality. Finally, CMOT uses the k-Nearest-Neighbors (kNN) of source cells to infer missing modality for target cells. Moreover, CMOT does not need paired multi-modal data for alignment. We found that not only does CMOT outperform existing state-of-art methods, but its inferred gene expression is biologically interpretable by evaluating on emerging single-cell multi-omics datasets. Finally, CMOT is open source at: https://github.com/daifengwanglab/CMOT.

## Results

### Overview of Cross-Modality Optimal Transport

**Fig. 1:**
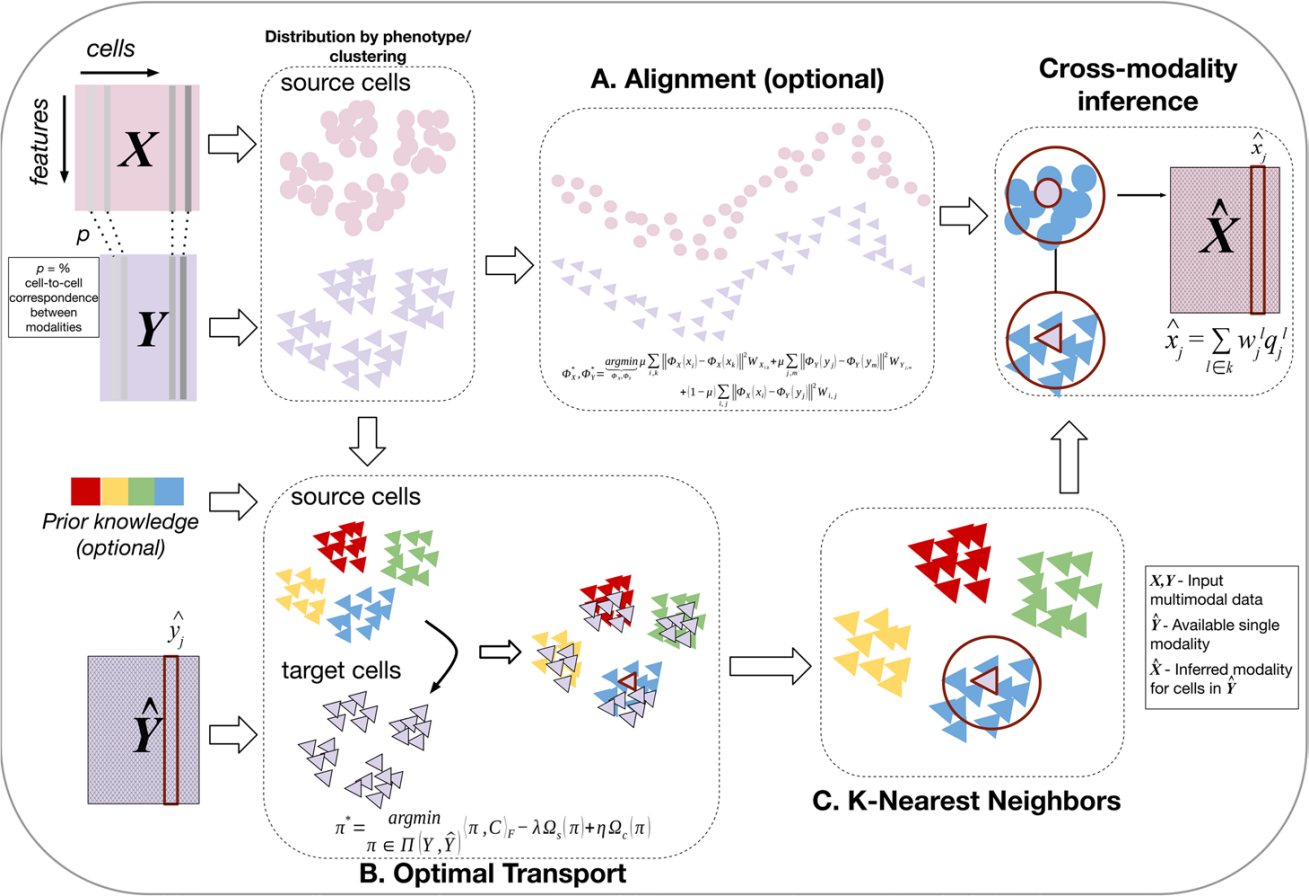
Cross-Modality Optimal Transport (CMOT): CMOT is a computational approach to infer missing modalities for existing single-cell modalities. It has three main steps: **A** Alignment (optional), **B** Optimal Transport, and (**C**) k-Nearest Neighbors inference. CMOT inputs two multi-modalities $X$ and $Y$ (source), where the cells in $X$ and $Y$ need not be completely corresponding. The cell-to-cell correspondence information between $X$ and $Y$ can be specified through $p$. CMOT aligns $X$ and $Y$ using non-linear manifold alignment (NMA) onto a common low-dimensional latent space if cells in $X$, $Y$ do not have complete correspondence. Then, CMOT uses optimal transport (OT) to map the cells in source $Y$ to the cells in target $\widehat{Y}$, where $Y$ and $\widehat{Y}$ share modalities. CMOT minimizes the cost of transportation by finding the Wasserstein distance between cells in $Y$ and $\widehat{Y}$ which is further regularized by prior knowledge or induced cell clusters and entropy of transport. Finally, CMOT infers the missing modality $\widehat{X}$ for cells in $\widehat{Y}$ using k-Nearest Neighbors (kNN). It calculates a weighted average of the k-nearest mapped cells in *Y* for every cell in $\widehat{Y}$, using their values from $X$, and infers $\widehat{X}$

CMOT (Cross-Modality Optimal Transport) is a computational approach for cross-modality inference of single cells (Fig. 1). CMOT accepts available multimodal and single modality datasets as inputs. CMOT does not require that the available multimodalities have complete corresponding information, i.e., allowing a fraction of unmatched cells in the source.

CMOT first aligns a group of cells $X$ and $Y$ (source) within available multi-modal data onto a common latent space (Step A), if the cells across multimodalities do not have complete correspondence. However, this is an optional step if the cells across multimodalities have complete correspondence. In this study, we used Non-linear Manifold Alignment (NMA) [19] to align the unmatched multimodalities. Next, CMOT applies optimal transport to map the cells with a single modality $\widehat{Y}$ (target) to cells in the source from the same modality $Y$ by minimizing their cost of transportation using Wasserstein distance (Step B). This distance can be regularized by prior knowledge (e.g., cell types) or induced cell clusters to improve mapping, and an entropy of transport to speed up OT computations. The optimal transport optimization tries to find an efficient mapping ${\pi }^{*}$ between cells of $Y$ and $\widehat{Y}$ that is used to transport cells in $Y$ to the same space as cells in $\widehat{Y}$. Once transported, CMOT uses k-Nearest-Neighbors to infer the missing modality $\widehat{X}$ for the cells in target $\widehat{Y}$ (Step C). Here, the missing or additional modality $\widehat{X}$ inferred by CMOT has the same number of features as $X$, and in the same space as $X$. Details about each step can be found in the “Methods” section.

We benchmarked CMOT with state-of-art methods [5, 7,-9, 11, 20, 21] on large-scale single-cell multi-omics (e.g., scRNA-seq and scATAC-seq (Additional File 1: Fig. S1A, Fig. S2A, Fig. S3A, Fig. S4A)). Also, we applied CMOT to additional omics datasets like protein expression. These datasets span across broad contexts including human and mouse brains, cancers and immunology, showing the generalizability of CMOT.

### Single-cell gene expression inference from chromatin accessibility in human and mouse brain

#### Human brain

We first applied CMOT to single-cell human brain data with jointly profiled chromatin accessibility and gene expression by 10xMultiome (scATAC-seq and scRNA-seq of 8981 cells) and inferred gene expression of cells from open chromatin regions (OCRs by peaks from scATAC-seq) [1]. We selected the top 1000 most variable genes & peaks (Additional File 1: Supplementary Methods). We randomly split the cells into 80% training for cross-validation and 20% testing set for evaluation. We split the training set into training and validation to find optimal parameters for the model using 5-fold cross-validation. For the alignment, we set *K* = 5, and latent dimension *d* = 20. For optimal transport, we set parameters λ = 200 and η = 1. For kNN modality inference, we set *k* = 600. Also, we used 10 major brain cell types from the dataset. However, to test CMOT’s performance when such cell-type information is absent, we induced cell labels by two major cell clusters. We also tested CMOT’s performance for different levels of correspondence: *p* = 25%, 50%, 75%, 100%.

**Fig. 2:**
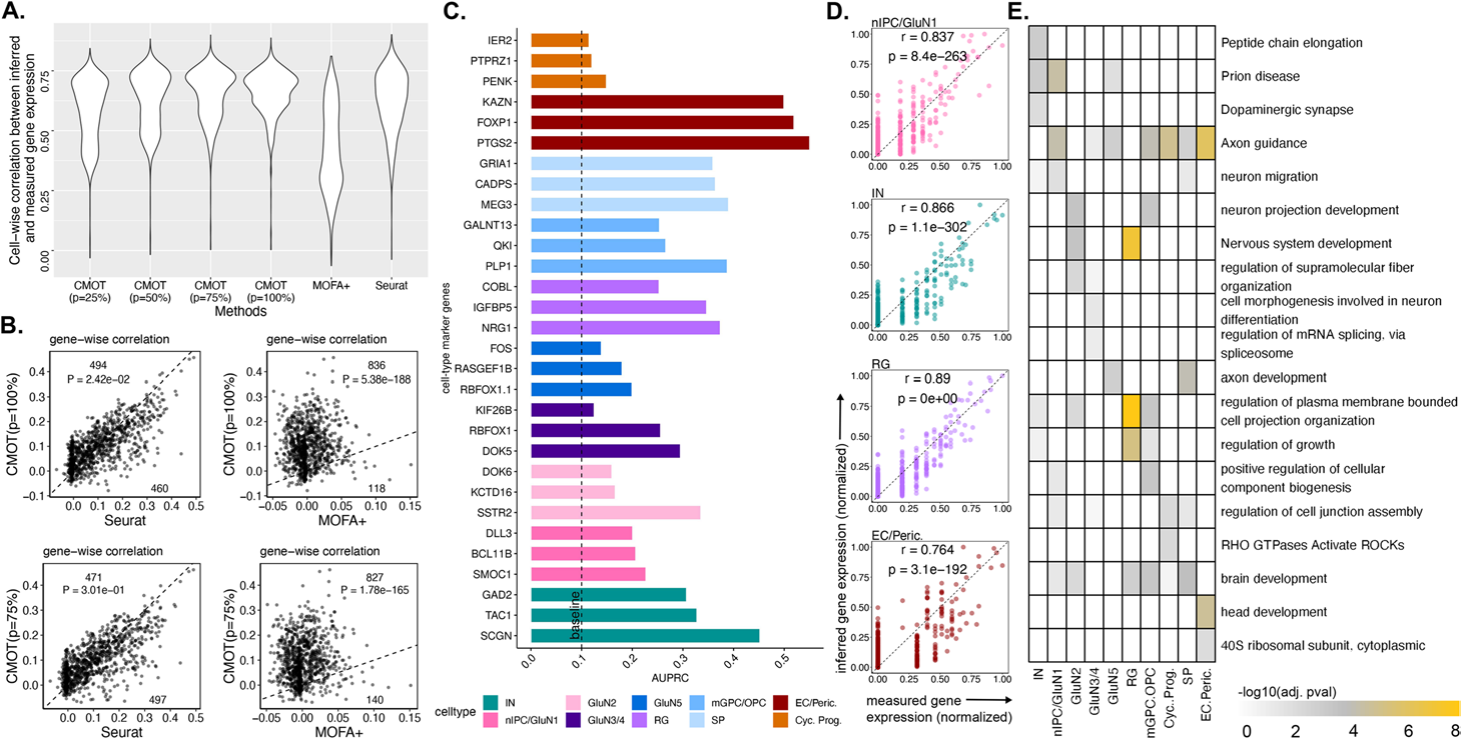
Single-cell gene expression inference from chromatin accessibility in developing human brain.** A** Cell-wise Pearson correlation (*y*-axis) of inferred and measured gene expression by different methods (*x*-axis): CMOT (*p* = 25%, 50%, 75%, 100%), Seurat, MOFA + (Additional File 1: Table S1-S4). See Additional File 1: Fig. S1 A and Additional File 1: Supplementary Methods for additional benchmarking. **B** Gene-wise correlation between the inferred and measured gene expression, comparing CMOT (*y*-axis) with MOFA + and Seurat (*x*-axis). Dots: Genes; Numbers: numbers of genes with improved inference by comparing methods. *P*-values are from one-sided Wilcoxon rank-sum tests. **C** Gene-wise AUPRC of cell type marker gene for classifying the cell type. Dashline: baseline = 0.1 (see the “Methods” section). **D** The measured (*x*-axis) versus inferred normalized expression (y-axis) of genes (dots) for one cell across four cell types: nIPC/GluN1(first), E.C./Peric. (second), IN (third), R.G. (last). **E** Heatmap showing different enriched terms ranked by -log10(adj. p-value of enrichment) values for the top 100 highly predictive genes within each cell type (see the “Methods” section). *r* is the Pearson correlation coefficient. *p* is the correlation *p*-value

CMOT achieves a strong performance for gene expression inference on the testing data, outperforming state-of-the-art methods like Seurat and MOFA + (Fig. 2A, B). For instance, CMOT reports a mean cell-wise Pearson correlation* r* = 0.66 for *p* = 100%, significantly higher than both MOFA + (median *r* = 0.43, Wilcoxon rank-sum test *p*-value = 0) and Seurat (median *r* = 0.62, Wilcoxon *p*-value < 1.23e − 14). Even for partial correspondences, CMOT has significantly higher performances (median *r* = 0.65 for *p* = 75%, and *r* = 0.63 for *p* = 50%) than MOFA + (Wilcoxon *p*-value < 2.8e − 294) and Seurat (Wilcoxon *p*-value < 3.43e − 10). Also, with low correspondence such as *p* = 25%, CMOT’s performance is still significantly higher than MOFA + (Wilcoxon *p*-value < 1.65e − 157). For gene-wise correlation (Fig. 2B), CMOT *p* = 100% and *p* = 75%, both outperform MOFA + for 836 versus 118 genes (Wilcoxon *p*-value < 5.38e − 118) and 827 versus 140 genes (Wilcoxon *p*-value < 1.78e − 165), respectively (Fig. 2B). Also, CMOT *p* = 100% outperforms Seurat for 494 versus 460 genes (Wilcoxon *p*-value < 2.42e − 2). For CMOT *p* = 75%, Seurat slightly performs better for 497 genes versus 471 for CMOT (Wilcoxon *p*-value < 3.01e − 1).

Next, we evaluated if the CMOT inferred gene expression to classify brain cell types. We used known cell-type marker genes provided along with the dataset and selected the top 8 highly predictive cells from each cell type within our inferred gene expression (see Additional File 2). Due to high imbalance of the number of cells within each cell type, we picked the number of cells based on the size of the smallest cell type. In this case, the cell type EC/Peric. had only 8 cells; therefore, we picked only 8 cells from the other cell types. We then calculated the AUPRC of the respective inferred genes in a one-vs-all manner for each cell type against the rest (“Methods”). CMOT obtains the higher AUPRCs for these genes against a *baseline* of 0.1 (Fig. 2C) for all cell types. The *baseline* is defined as the proportion of positives in the data. This suggests that the CMOT inferred expression is capable to distinguish cell types, providing the biological interpretability of the CMOT inference. Looking at individual cells (Fig. 2D), CMOT infers individual cell expression with high Pearson correlation and significance (*p* < 0.05). Furthermore, we also found the enriched functions and pathways relating to brain development from the top 100 highly predictive genes (Fig. 2E). For results of benchmarking on additional state-of-art methods, see Additional File 1: Fig. S1, Tables S1-S4, and Supplemental Methods.

Furthermore, we benchmarked CMOT with the state-of-art methods on a SNARE-seq dataset [22] consisting of jointly profiled gene expression and chromatin peaks in adult mouse brain (see Additional File 1: Fig. S2, Fig. S3, Tables S5-S8, S9-S10, and Supplemental Methods).

### Inferring protein expression from gene expression in peripheral blood mononuclear cells

We applied CMOT to infer protein expression from gene expression of peripheral blood mononuclear cells (PBMCs) using emerging CITE-seq data [5]. We trained CMOT on 6885 cells from PBMC10k, with parameters: *K* = 5, *d* = 15, λ = 1e02, η = 1, *k* = 100, and used the top 200 highly variable genes in the training data to find the *k* nearest neighbors. We induced cell labels by identifying two clusters using gene expression for the label regularization in optimal transport. We evaluated CMOT, MOFA + , Seurat, and TotalVI’s using 3994 cells from a different dataset, PBMC5k. Here we show an independent evaluation of CMOT and other methods on PBMC5k while using PBMC10k as the training data (Additional File 1: Tables S11-S13). Additionally, we also show benchmarking on PBMC10k, by splitting it into 80% training and 20% testing data (see Additional File 1: Fig. S7, Supplementary Methods, and Tables S14-S16).

**Fig. 3:**
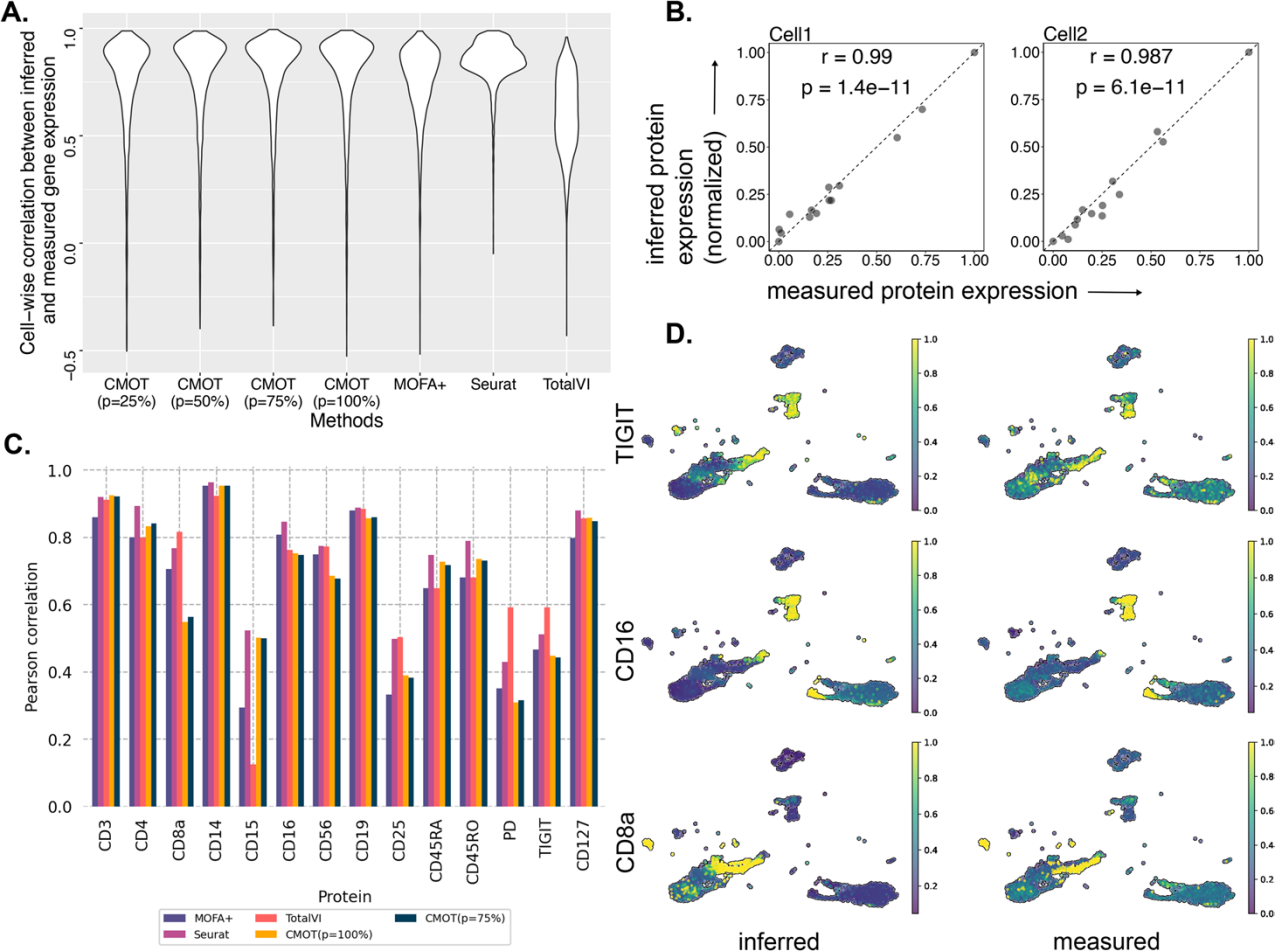
Inferring protein expression from gene expression in single-cell peripheral blood mononuclear cells.** A** Cell-wise Pearson correlation (*y*-axis) of inferred and measured protein expression by different methods (*x*-axis): CMOT (*p* = 25%, 50%, 75%, 100%), Seurat, MOFA + , TotalVI (Additional File 1: Tables S11-S12). **B** The measured (*x*-axis) versus inferred normalized expression (*y*-axis) of 14 proteins (dots) for two select cells. **C** Pearson correlations of inferred and measured expression (*y*-axis) of individual proteins (*x*-axis) by CMOT (*p* = 100%, 75%), Seurat, MOFA + , TotalVI (Additional File 1: Table S13). **D** UMAPs of inferred and measured expressions for three proteins: TIGIT (*r* = 0.45; *p* = 6.18e − 197) (top), CD16 (*r* = 0.75; *p* = 0) (middle), CD8a (*r* = 0.55; *p* = 3.11e − 312) (bottom) (Additional File 1: Table S13). The intensity represents the protein expression level. r is the Pearson correlation coefficient. p is the correlation *p*-value

As shown in Fig. 3A, CMOT achieves a median cell-wise Pearson correlation = 0.86 for *p* = 100%, significantly outperforming MOFA + (*r* = 0.79, Wilcoxon *p*-value < 6.9e − 57) and TotalVI (default parameters) (*r* = 0.61, Wilcoxon *p*-value 0) as well as comparable with Seurat. For instance, we show two cells and their Pearson correlation of inferred versus measured protein expressions (*r* = 0.99, *p* = 1.4e − 11 and *r* = 0.98, *p* = 6.1e − 11) in Fig. 3B. Moreover, even for partial correspondences, *p* = 25%, 50%, 75%, CMOT performs consistently with significantly higher cell-wise correlation than MOFA + (Wilcoxon *p*-values 0,0,0) and TotalVI (Wilcoxon *p*-values < 8.36e − 58, 1.73e − 45, 5.25e − 12). Also, for inferring individual protein expression, CMOT has high correlations for all proteins, consistent with state-of-art methods (Fig. 3C), with some examples shown in Fig. 3D. Rest of the proteins along their inference statistics are reported in Additional File 1: Fig. S5 and Additional File 1: Tables S11-S13.

### Inference of gene expression using chromatin accessibility for drug-treated lung cancer cells

**Fig. 4:**
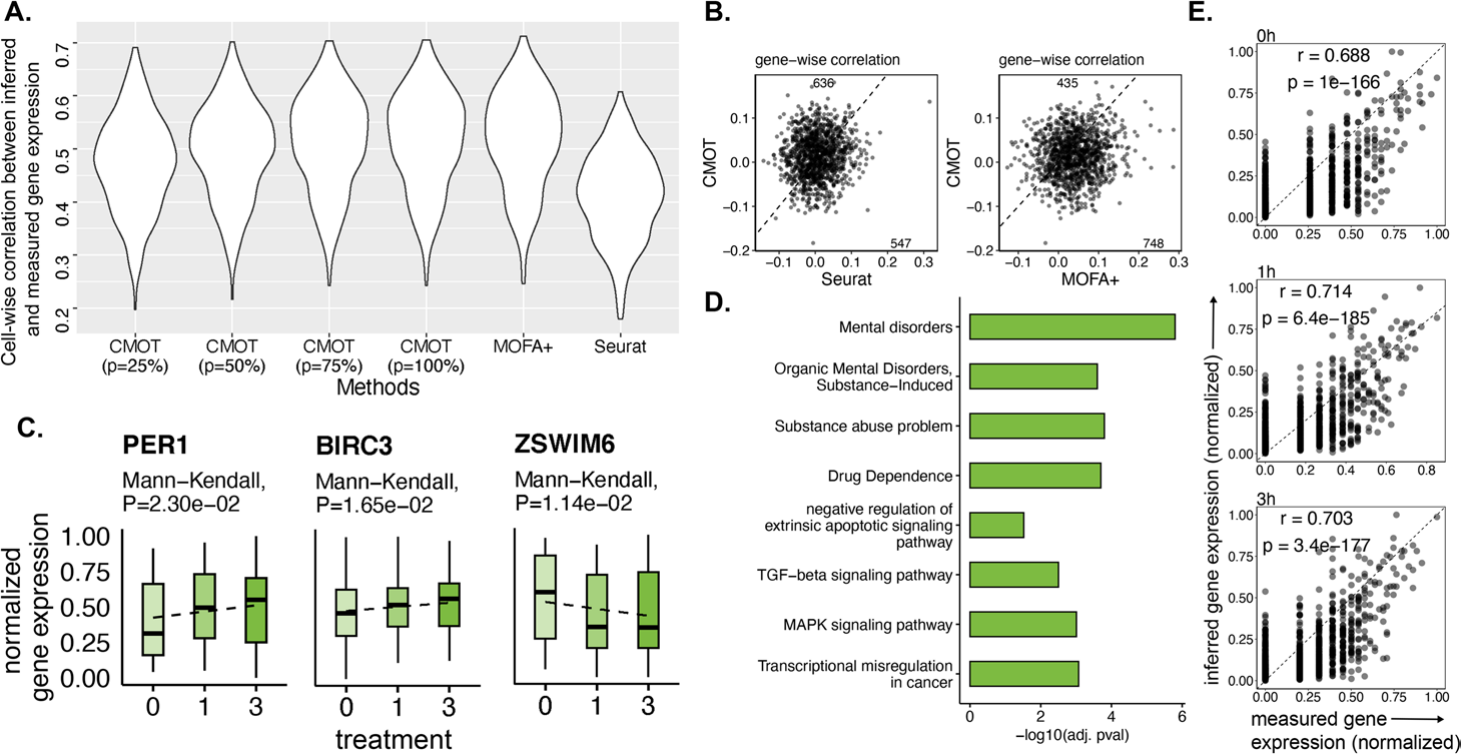
Inference of gene expression for drug-treated A549 lung cancer cells using chromatin accessibility.** A** Cell-wise Pearson correlation (*y*-axis) of inferred and measured gene expression by different methods (*x*-axis): CMOT (*p* = 25%, 50%, 75%, 100%), Seurat, MOFA+ (Additional File 1: Tables S17-S20). See Additional File 1: Fig. S4 A and Supplementary Methods for additional benchmarking. **B** Gene-wise correlation between the inferred (*y*-axis) and measured (*x*-axis) expression, comparing CMOT with MOFA + and Seurat. Dots: Genes; Numbers: Gene numbers above and below the dotted line. *P*-values are calculated by a one-sided Wilcoxon rank-sum test (Additional File 1: Table S21). **C** CMOT inferred normalized gene expression trend (*y*-axis) across treatment hours (*x*-axis). Key genes: PER1 and BIRC3 [23–25] are markers for glucocorticoid receptor (GR) activation seen later in treatment (3 h). ZSWIM6 [26] is a key gene of early events of DEX treatment (0 h, 1 h) (see Additional File 2 for top 100 highly predictive genes). **D** Enriched terms associated with CMOT inferred gene expression using 435 genes with a higher gene-wise Pearson correlation compared to MOFA+ ’s 748 genes (**B**, Additional File 1: Fig. S6B). **E** The measured (*x*-axis) versus inferred normalized expression (*y*-axis) of genes (dots) for three select cells. *r* is the Pearson correlation coefficient. p is the correlation *p*-value

Next, we applied CMOT to 100nM dexamethasone (DEX)-treated A549 single-cells from lung adenocarcinoma. The DEX-treated 2641 cells were profiled after 0, 1, and 3 h of treatment for gene expression and open chromatin regions (OCRs) using sci-CAR experiments [2]. We focus on the CMOT’s performance for gene expression inference from peak signals of OCRs. We stratified-split the dataset into 80% training and 20% test cells using the treatment hours. We used the treatment hours as the classes for label regularization in optimal transport for training cells. We trained CMOT with the parameters *K* = 5, *d* = 10, λ = 1e02, η = 5e − 3, and *k* = 500 and used the top 20 highly variable OCRs in scATAC-seq to find the *k* nearest neighbors. Again, we found that CMOT shows a consistent performance across different cell-to-cell correspondence information (*p*) with high correlation. CMOT (*p* = 100%) infers gene expression with a mean Pearson correlation of 0.52, similar to MOFA + and outperforming Seurat (median correlation = 0.5, Wilcoxon *p*-value < 1.27e − 05) (Fig. 4A).

Moreover, CMOT shows a high gene-wise Pearson correlation outperforming Seurat for 636 versus 547 genes (Fig. 4B, Additional File 1: Table S21). Although MOFA + reports a higher gene-wise Pearson correlation for some genes than CMOT (Fig. 4B, Additional File 1: Table S21), we still see that CMOT’s inferred expression shows the transitory trend of key druggable marker genes across drug-treatment hours. Figure 4C shows three key genes, identified as makers of early (ZSWIM6) [26] and late (PER1, BIRC3) [23–25] events of treatment. Also, we performed enrichment of the 435 high correlation genes identified by CMOT (versus MOFA + in Fig. 4B) (see Additional File 3 for list of genes). As shown in Fig. 4D, we saw a higher enrichment of terms associated with DEX-treated A549 cells like TGF-beta signaling, along with effects on DEX treatment in general, like Mental disorders as compared to enrichment given by MOFA + (Additional File 1: Fig. S6, Additional File 2). Lastly, we also found that the cell-wise correlations between inferred and measured gene expression are also significantly highly correlated in each treatment hour (Fig. 4E). For results of benchmarking on additional state-of-art methods, see Additional File 1: Fig. S4, Supplemental Tables S17-S20, and Supplemental Methods.

### Cross-modality inference between gene expression and chromatin accessibility to distinguish cancer types

Finally, we tested CMOT to see how well it can infer between two modalities, especially for relevantly small datasets. We used a pan-cancer scCAT-seq dataset which jointly profiled 206 single-cell gene expression and chromatin accessibility on OCRs for three cancer cell lines: HCT116, HeLa-S3, and K562 [3]. We stratified split data into 80% training and 20% testing sets using cancer-type information. We induced our cell labels for training cells for label regularization in optimal transport. For gene expression inference from OCR peaks, we identified two clusters in chromatin peaks and vice versa. We trained CMOT with the following parameters for gene expression inference from chromatin peaks: *K* = 5, *d* = 10, λ = 5e03, η = 1, *k* = 40, and used the top 150 highly variable OCRs to find the *k* nearest neighbors. For inferring gene expression from binarized OCR peaks, we evaluated the inferred expression using the same metrics (cell-wise and gene-wise Pearson correlation) as above.

**Fig. 5:**
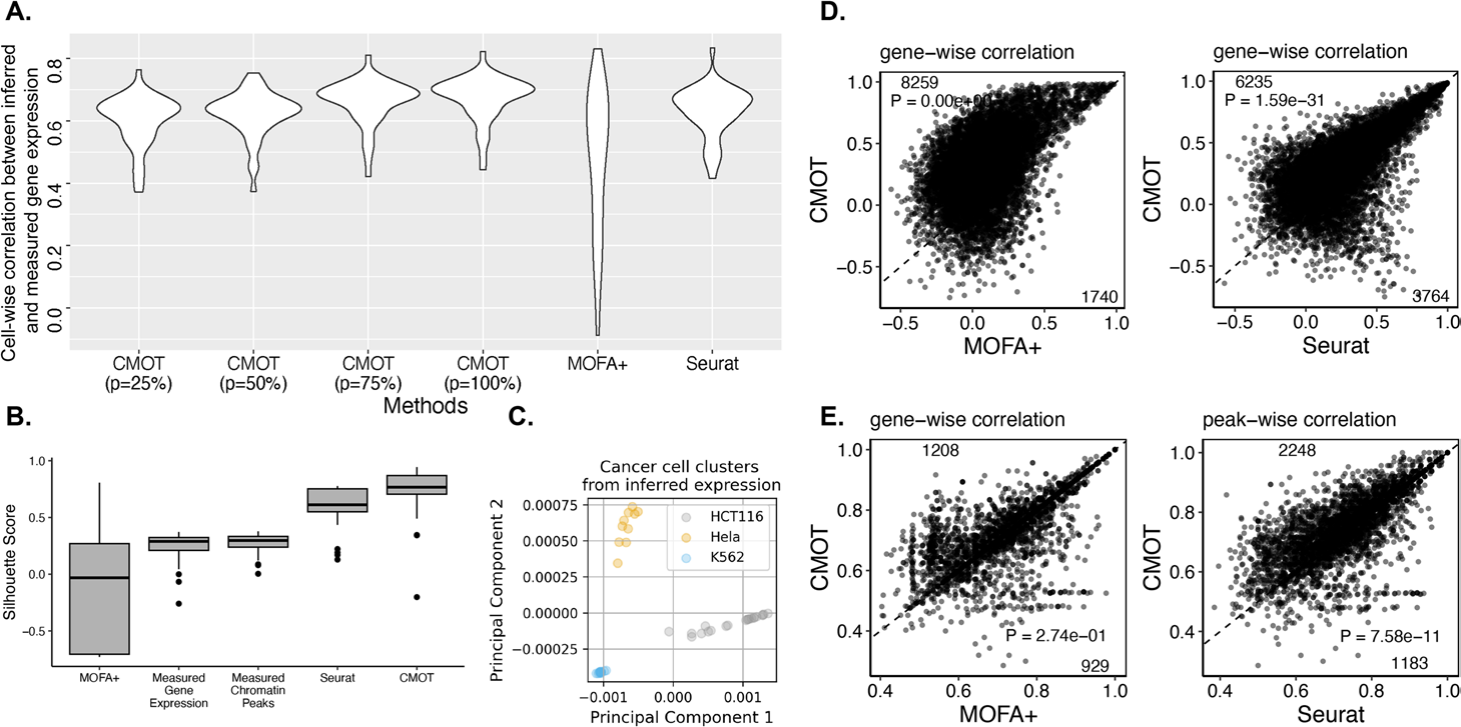
Cross-modality inference between gene expression and chromatin accessibility can distinguish cancer types.** A** Cell-wise Pearson correlation (*y*-axis) of inferred and measured gene expression by different methods (*x*-axis): CMOT (*p* = 25%, 50%, 75%, 100%), Seurat, MOFA + (Additional File 1: Tables S22-S25). **B** Silhouette score (*x*-axis) across measured and inferred gene expressions (*x*-axis), and measured chromatin peaks (Additional File 1: Table S26). **C** PCA of inferred gene expression. **D** Gene-wise correlation between the inferred and measured expression, comparing CMOT (*y*-axis) with MOFA + and Seurat (*x*-axis). Dots: Genes; Numbers: Gene numbers above and below the dotted line. **E** Peak-wise AUROC, comparing CMOT (*y*-axis) with MOFA + and Seurat (*x*-axis). Dots: Peaks; Numbers: Peak numbers above and below the dotted line. *P*-values are calculated by a one-sided Wilcoxon rank-sum test

CMOT significantly outperforms both MOFA + and Seurat, with a cell-wise mean correlation of 0.67 compared to 0.47 (Wilcoxon *p*-value < 6.81e − 17) and 0.63 (Wilcoxon *p*-value < 1.32e − 05), respectively (Fig. 5A, Additional File 1: Tables S22-S25). Moreover, CMOT (*p* = 100%) yields an improved gene-wise correlation for 6235 genes versus 3764 against Seurat (Wilcoxon *p*-value < 1.59e − 31), and 8259 versus 1740 against MOFA + (Wilcoxon *p*-value = 0) (Fig. 5D). Moreover, CMOT’s inference is particularly useful to identify the cancer type specific cell clusters. For instance, we calculated the silhouette score (see the “Methods” section) to see if the cells from the same cancer lines exhibit similar gene expression patterns. CMOT reports a high median silhouette score of 0.74 compared to the measured gene expression (0.25), measured chromatin peaks (0.27), and inferred expressions from Seurat (0.61) and MOFA + (− 0.07) (Fig. 5B, Additional File 1: Table S26). As shown in Fig. 5C, the cancer cells from three cancer cell lines can be separated using CMOT-inferred gene expression, suggesting the capability of CMOT inference to reveal cancer-type-specific expression. Then, we evaluated the CMOT’s OCR peaks inference from gene expression. We trained CMOT with the parameters: *K* = 5, *d* = 10, λ = 1e03, η = 1, *k* = 10, and used the top 50 highly variable genes to find the *k.*nearest neighbors. We also stratified split the data into 80% training and 20% testing sets using cancer-type information. We normalized CMOT’s inferred peaks and then binarized them by a cutoff of 0.5, and then calculated the peak-wise area under the receiver operating curve (AUORC) of the inferred binarized peaks relative to the binarized measured profile. We also found that CMOT significantly outperforms both MOFA + and Seurat with Wilcoxon *p*-values < 9.42e − 77 and 9.48e − 10, respectively, for OCR peak inference from gene expression (Fig. 5E, Additional File 1: Fig. S8, Additional File 1: Table S27-S28).

## Discussion

With the advent of new single-cell technologies, data is generated with even greater precision. However, most of these technologies continue to profile a single modality for each cell, creating a need for robust cross-modality inference frameworks to jointly study the underlying that can infer additional or missing modalities for such cells.

In this study, we introduce CMOT as a computational approach that integrates manifold alignment, regularized optimal transport, and k-Nearest Neighbors (kNN) for cross-modality inference. By applying emerging single-cell multimodal data, we demonstrated that CMOT was able to predict multimodal features of single cells such as gene expression, chromatin accessibility, and protein expression. Note that CMOT does not require paired samples for aligning multimodalities as shown by its out performances over state-of-arts in some applications. This attribute is particularly useful since joint multimodal profiling is typically challenging and sometimes costly and single modality data is thus still widely favored. To demonstrate this, we evaluated CMOT and other state-of-art methods on single-profile scRNA-seq and scATAC-seq dataset [1] and found that CMOT outperforms all methods (Additional File 1: Fig. S14). Moreover, CMOT is more computationally efficient and faster than state-of-art methods (Additional File 1: Table S29). In the paper, CMOT primarily used nonlinear manifold alignment (NMA) to align multimodalities for achieving the best inference. However, CMOT is flexible and the user can substitute NMA with their preferred alignment method, e.g., SCOT [15, 16], MMD-MA [27], and WNN [8].

Furthermore, the optimal transport step in CMOT leverages the information within shared modalities between source and target cells to compute a mapping matrix through Wasserstein distances between them. These distances quantify and minimize the geometric discrepancy of the distributions and map the two distributions for improving cross-modal inference in CMOT.

Additionally, we see that the overall correlation scores of all methods consistently vary across datasets. We attribute this variation in correlation across datasets to factors like the number of available cells, total features used, and the sparsity of the datasets used in training. We noticed a higher correlation for datasets either with more training cells or a high number of features. However, data sparsity continues to be a challenge with single-cell profiling technologies and therefore affects inference performance for such datasets.

Nonetheless, we also examined CMOT’s potential limitations. First, nonlinear manifold alignment comes with a high computational cost as the data size increases. It needs to compute Laplacians and similarity matrices of multimodal inputs which scale quadratically with the datasets and slow CMOT’s computations over large datasets. However, this can potentially be sped up by using other alignment methods like SCOT [15, 16] or Unioncom [28]. Second, Optimal transport assumes a mass-balancing approach between the source and target distributions, where every s (e.g., a cell) in the source has to map to a point in the target. This is a relatively strong assumption requiring a balanced data distribution between the source and target to fit a conservative transport plan. Given many imbalanced datasets in the real world, this limitation can be improved by recent optimal transport techniques such as SCOTv2 [16] through emerging unbalanced optimal transport approaches [29, 30].

Also, CMOT can adapt other optimal transport variants to even transport different modalities between the source and target cells. For instance, Gromov Wasserstein distance can map distributions from different modalities [31, 32]. Moreover, CMOT has the potential to work with additional single-cell modalities like the morphology and electrophysiology of single neuronal cells from Patch-seq [33]. In addition to inferring modalities, CMOT has the potential to be extended to infer the sample labels such as phenotypes across modalities, e.g., via label transferring [8]. For example, it can predict cell types or disease states of single cells for the modalities without such information.

## Conclusion

In this study, we introduced CMOT as a computational framework that can successfully infer additional or missing modalities for cells with single modalities. We applied CMOT to single-cell datasets of different scales and profiles (e.g., gene expression, peaks, proteins), which can be easily extended to other modalities and applications. CMOT uses the underlying data distributions of multimodalities and uses optimal transport to find efficient mappings between available multimodalities and the target single modality, without requiring prior assumptions about their distributions. Moreover, CMOT’s design is computationally efficient.

## Methods

### Cross-Modality Optimal Transport (CMOT) workflow

CMOT workflow for cross-modality inference can be divided into 3 steps including an optional first step:

- Step A (optional): Alignment to project the cells with available multimodal data (source cells) onto common low-dimensional latent space.
- Step B: Optimal transport to map cells with the single modality (target cells) to the aligned source cells from the same modality.
- Step C: k-Nearest-Neighbors to infer the missing or unprofiled modality of target cells using another modality of nearest mapped source cells.

We describe each step in detail below by introducing the necessary notations:

#### Step A: Alignment of source cells with multimodal data

We set this as an optional step if the cells across available multimodalities do not have a complete correspondence. That is, if cells across modalities have none to partial correspondence between them, then CMOT first aligns them. Although users are free to use their choice of alignment in such scenarios, we use Nonlinear Manifold Alignment (NMA) [19].

Alignment is an important step that accounts for when the source cells have partial correspondence. NMA is based on a manifold hypothesis that high dimensional multimodal datasets have similar underlying low dimensional manifolds, and therefore, they can be projected onto a common manifold space that preserves the local geometry of each modality and minimizes the differences between the manifolds of modalities. We define $X=\{{x}_{i}{\}}_{i=1,..,{s}_{X}}$ and $Y=\{{y}_{j}{\}}_{j=1,..,{s}_{Y}}$ as two multimodal measurements of ${s}_{X}$, ${s}_{Y}$ source cells in Modalities $X$, $Y$ respectively, where ${x}_{i}\in {R}^{{d}_{X}}$ and ${y}_{j}\in {R}^{{d}_{Y}}$ represent the measurements of ${d}_{X}$ features in ${i}^{th}$ cell of Modality $X$, and ${d}_{Y}$ features in ${j}^{th}$ cell of Modality $Y$, respectively. We also define ${W}_{X}\in {R}^{{s}_{X}\times {s}_{X}}$ and ${W}_{Y}\in {R}^{{s}_{Y}\times {s}_{Y}}$ as cell similarity matrices for $X$ and $Y$, respectively, where each similarity matrix is constructed by connecting a cell with its $K$ nearest neighboring cells within the modality. The partial prior known cell-to-cell correspondence information can be quantified by $p$ (0 < *p* < 100%) to quantify the partial prior known cell-to-cell correspondence information (for example, $p\text{\%}$ of paired cells across modalities) and encode this information as a cross-modal similarity matrix $W\in {R}^{{s}_{X}\times {s}_{Y}}$. NMA then learns two mapping functions ${\Phi }_{X}$ and ${\Phi }_{Y}$ that project ${x}_{i}$ and ${y}_{j}$ to ${\Phi }_{X}\left({x}_{i}\right)\in {R}^{d}$ and ${\Phi }_{Y}\left({y}_{j}\right)\in {R}^{d}$, respectively onto a common manifold space with dimension $d\ll min\left({d}_{X},{d}_{Y}\right).$ The $d$-dimensional manifold preserves the local geometry of each modality and minimizes the distances between corresponding samples after projection. Solving manifold alignment can be reformulated as manifold co-regularization in reproducing kernel Hilbert spaces. The manifold alignment optimization finds optimal mapping functions ${\Phi }_{X}^{*}$, ${\Phi }_{Y}^{*}$ by solving the following:

$$\Phi_X^\ast,\Phi_Y^\ast=\begin{array}{c}\underset{\Phi_X,\Phi_Y}{\underbrace{argmin}\;}\mu\sum\nolimits_{i,k}{\Arrowvert\Phi_X\left(x_i\right)-\Phi_X\left(x_k\right)\Arrowvert}^2W_{X_{i,k}}+\mu\sum\nolimits_{j,m}{\Arrowvert\Phi_Y\left(y_j\right)-\Phi_Y\left(y_m\right)\Arrowvert}^2W_{Y_{j,m}}\\+\left(1-\mu\right)\sum\nolimits_{i,j}{\Arrowvert\Phi_X\left(x_i\right)-\Phi_Y\left(y_j\right)\Arrowvert}^2W_{i,j}\end{array}$$,

 where the first two terms preserve the local geometry within each modality, the similarity matrices ${W}_{X}$ and ${W}_{Y}$ model the relationships of the cells in each modality that can be identified by *K*-nearest neighbor graph, and the third term preserves the correspondence information across $X$ and $Y$ modeled by $W$. The parameter $\mu$ controls the trade-off between conserving the local geometry of each modality and cell-to-cell correspondences across modalities. Here, we set $\mu$ to 0.5. This allows equal importance to both preserving the local geometry of each modality as well as cell-to-cell correspondences, thereby eliminating the need for assuming underlying assumptions about data distributions of modalities.

We also need to add an additional non-zero constraint to avoid mapping of all cells onto a latent space with dimension zero: ${P}^{T}DP = I$, where $$P=\left[\begin{array}{c}{\Phi }_{X}\\ {\Phi }_{Y}\end{array}\right]$$, ${\Phi }_{X}={\left[{\Phi }_{X}\left({x}_{1}\right),....,{\Phi }_{X}\left({x}_{{s}_{X}}\right)\right]}^{T}$, ${\Phi }_{Y}={\left[{\Phi }_{Y}\left({y}_{1}\right),....,{\Phi }_{Y}\left({y}_{{s}_{Y}}\right)\right]}^{T}$, $D$ is the diagonal matrix with  $diag({\sum_i W}^{1,i}_{X} ... {\sum_i W}^{S_X,i}_{X})$ and $diag({\sum_j W}^{1,j}_{Y} ... {\sum_j W}^{S_Y,j}_{Y})$ as diagonal elements, and $I$ is the identity matrix [18, 34].

Also, two modalities are not required to have a complete correspondence between the cells. Therefore, $W$ is a binary correspondence matrix between cells of $X$ and $Y$ such that if $p$=100%, i.e., 100% correspondence across cells in $X$ and $Y$, $W$ would be an identity matrix. For $p$<100%, ${W}_{i,j}=1$ if ${x}_{i}$ and ${j}^{th}$ cells from Modalities $X$ and $Y$ respectively are the corresponding cells and 0 otherwise. After alignment, the resulting $d$-dimensional modalities share a common latent space that can easily be compared using Euclidean distances. For instance, for every cell ${y}_{j}\in Y$, we find an aligned cell ${x}_{j,a}\in X$ by finding the closest cell in $X$ using the Euclidean distance. To implement our alignment step, we used the non-linear manifold module from our published Python package ManiNetCluster [34].

Unless otherwise stated, we use the term CMOT for our model trained with full correspondence ($p$=100%).

#### Step B: Optimal Transport to map source and target cells by shared modality

The optimal transport theory [35, 36] tries to find the most efficient mapping ${\pi }^{*}$ that transports one probability distribution to another with minimum transportation cost. A mapping $\pi\in\prod\left(Y,\widehat{Y}\right)$ represents transport plan to map cells from source ($Y$) and target ($\widehat{Y}$) modalites, where $\prod\left(Y,\widehat{Y}\right)$ contains all probabilistic mappings between the source ($Y$) and target ($\widehat{Y}$) cells. We define $\widehat{Y}=\{{\widehat{y}}_{j}{\}}_{j=1,..,{s}_{\widehat{Y}}}$ as the target single modality measurement with ${s}_{\widehat{Y}}$ cells, where $\widehat{{y}_{j}}\in {R}^{{d}_{Y}}$ represents the measurement of ${d}_{Y}$ features in ${j}^{th}$ cell of Modality $\widehat{Y}$. The classical OT distance (Wasserstein distance) gives the mappings between two probability distributions as the transportation cost. Let $C$ be the cost matrix where ${C}\in {R}^{+{s}_{Y}\times {s}_{\widehat{Y}}}$ and ${C}_{i,j}$ represents the pairwise cost of mapping the source cell ${y}_{i}\in Y$ to the target cell $\widehat{{y}_{j}}\in \widehat{Y}.$ For discrete probability distributions like $Y$ and $\widehat{Y}$ over the same metric spaces (i.e., matched features of the shared modality), we define the OT problem as:

$${\pi }^{*}=\begin{array}{c}argmin\\ \pi \in \Pi \left(Y,\widehat{Y}\right)\end{array}{\langle \pi ,C\rangle }_{F}-\lambda {\Omega }_{s}\left(\pi \right)+\eta {\Omega }_{c}\left(\pi \right)$$

where the first term computes the Frobenius dot product ${\langle .,.\rangle }_{F}$ between the cost matrix $C$ and $\pi .$ The set $\prod$ is defined as $\prod \left(Y,\widehat{Y}\right)=\{\pi \in {R}^{+{s}_{Y}\times {s}_{\widehat{Y}}}:\pi {1}_{{s}_{\widehat{Y}}}={1}_{{s}_{Y}},{\pi }^{T}{1}_{{s}_{Y}}={1}_{{s}_{\widehat{Y}}}\}.$

The second term, also called entropic regularization, calculates the entropy of transportation for $\pi$ where ${\Omega }_{s}\left(\pi \right)=-{\sum }_{i,j}\pi \left(i,j\right)log\pi \left(i,j\right).$ Entropic regularization addresses the computational complexity of OT as the sample size increases [37]. The intuition behind this term is to relax the sparsity constraints of the OT problem by increasing its entropy so that ${\pi }^{*}$ is denser, as source cells ($Y$) are distributed more towards target cells ($\widehat{Y}$). The resulting formulation is strictly convex and can be solved through Sinkhorn’s Algorithm [22]. The parameter $\lambda$ weights the entropic regularization. As the parameter increases, the sparsity of ${\pi }^{*}$ decreases, giving a smoother transport.

The third term is the label regularizer [37], ${\Omega }_{c}={\sum }_{j}{{\sum }_{c}\Vert \pi \left({I}_{c},j\right)\Vert }_{p}^{q}$, where ${I}_{c}$ contains the index of rows in $\pi$ related to the source cells ($Y$) that belong to class $c$ if we have such prior knowledge, e.g., known cell types. Hence, $\pi \left({I}_{c},j\right)$ is a vector containing the coefficients of the ${j}^{th}$ target cell in $\widehat{Y}$. The norm ${\Vert .\Vert }_{p}^{q}$ denotes the ${l}_{p}$ norm to the power of $q$ (here we set *p*=1 and *q*=0.5). The parameter $\eta$ weights the label regularization. The intuition behind this term is to penalize the mappings that match together samples from different labels. This means that even if we do not have the label information for the target cells ($\widehat{Y}$), we can promote group sparsity within the columns of $\pi$ such that each target cell is only associated with a class. However, in the absence of such label information, we can compute our own labels through unsupervised clustering techniques like hierarchical clustering to induce cell clusters as labels for source cells in $Y$. Finally, to map the source cells ($Y$) to the target space ($\widehat{Y}$), we use barycentric mapping using ${Y}^{\left(t\right)}={\pi }^{*}\widehat{Y}$ [37]. Now, we can easily compare ${Y}^{\left(t\right)}$ and $\widehat{Y}$ using euclidean distance. To solve the regularized OT optimization step, we used Domain Adaptation functions (*ot.da*) provided in the Python package Python optimal transport (POT) [38].

##### Identify outlier cells in target modality

Some cells from the target modality ($\widehat{Y}$) may have a different distribution (e.g., belonging to a cell type absent in source modality). For such cells, the inference is difficult and may even lead to false predictions. To avoid this, we add an additional mechanism to identify and remove such cells. We use the optimal coupling ${\pi }^{*}$ and the cost matrix $C$ calculated in step B, and compute an element-wise dot product $P={\pi }^{\text{*}T}\circ {C}^{T}$, where $P\in {R}^{{s}_{\widehat{Y}}\times {s}_{Y}}$. Then, we find any outliers within $P$ using the Isolation Forest (IF) algorithm [39]. The Isolation Forest (IF) algorithm isolates samples by randomly selecting a feature and then randomly selecting a split value between the minimum and maximum values of the selected features. Since this algorithm is prone to the curse of dimensionality, we first apply principal component analysis (PCA) to $P$ and use components that explain at least 95% variance. We then apply the IF to the lower dimensional $P$. We tested this mechanism by randomly replacing cells with noise in the DEX-treated A549 dataset (see Additional File 1). Additionally, CMOT provides a warning to users informing them about the percentage of poorly mapped cells that can be removed to avoid incorrect inferences.

#### Step C: k-Nearest Neighbors to infer the additional modality of target cells

Finally, we apply k-Nearest Neighbors (kNNs) to infer the missing modality $\widehat{X}$ of target cells in $\widehat{Y}$. For each target cell $\widehat{{y}_{j}}\in \widehat{Y}$, we find its kNN in ${Y}^{\left(t\right)}$ using Euclidean distance. Let ${S}_{j}=\{{c}_{j}^{l}:l=\mathrm{1,2},..k\}$ be the set of $k$ nearest neighboring cells of $\widehat{{y}_{j}}$ in ${Y}^{\left(t\right)}$, where ${c}_{j}^{l}$ is a cell in ${Y}^{\left(t\right)}$. For cells in ${S}_{j}$, we use their values from the aligned modality $X$ to define another set${Q}_{j}=\{{q}_{j}^{l}:l=\mathrm{1,2},...k\}$, where ${q}_{j}^{l}$ represents the profile of the cell ${c}_{j}^{l}$ within the aligned modality $X$. Finally we calculate the weighted average of the profiles of all cells in ${Q}_{j}$ to get ${\widehat{x}}_{j}$. This is calculated as:

$${\widehat x}_j=\sum_{l=1}^kw_j^lq_j^l$$

where ${w}_{j}^{l}$ is the weightage given to ${q}_{j}^{l}$ such that ${w}_{j}^l={e}^{\left(-\sqrt{{\Vert \widehat{{y}_{j}}-{y}^{l}_{{S}_{j}}\Vert }^{2}}\right)}$. Thus, we get the corresponding modality $\widehat{X}$ for $\widehat{Y}.$ We used sklearn’s [40] nearest neighbor function for kNN implementation.

### Single-cell multi-omics datasets

We tested CMOT on four single-cell multiomics datasets: (1) Gene expression and chromatin accessibility of single cells in human and mouse brains (scRNA-seq and scATAC-seq) [1, 22]; (2) Gene and protein expression of peripheral blood mononuclear cells (CITE-seq) [5]; (3) Gene expression and chromatin accessibility of A549 lung cancer cells (sci-CAR) [2]; (4) Gene expression and chromatin accessibility of pan-cancer cells (scCAT-seq) [3]. All details on data and data processing are available in Additional file 1: supplementary methods.

#### Partial correspondence in multi-omics data

Joint profiling of single cells is challenging and therefore, it may not always be feasible to get completely corresponding cells across profiled modalities. In such scenarios, there could be partial to no correspondence across cells of multimodalities. For example, a 50% cell-to-cell correspondence between modalities means that only 50% of the cells have been jointly profiled between the modalities. As a result, training on partially corresponding multimodalities for cross-modality inference can lead to misleading or wrong inferences. Therefore, to address this problem, CMOT first aligns such partially corresponding datasets and then performs inference. In this paper, we have used datasets that have a 100% correspondence originally, so that we can validate the inference performance. However, we simulate different levels of cell-to-cell correspondence by setting the $p$ value in non-linear manifold alignment (Methods Step A). In particular, we randomly chose $p$ percent cells for whom correspondence information is assumed available, while the remaining cells are treated as non-corresponding. We report CMOT’s performance when trained on $p$=25%, 50%, 75%, 100% cell-to-cell correspondence levels, and show that CMOT’s cross-modality inference performance can beat state-of-the-art methods that require 100% cell-to-cell correspondence for training.

### Datasets preprocessing and feature selection

#### Human brain

The human brain dataset was generated by 10xGenomics, containing gene expression and open chromatin regions multiome data from the same cells (8981 cells) profiled from post-conceptual week 21 (PCW21) [1]. We filtered out peaks and genes that occurred in less than 3 cells. For scATAC, we normalized the peaks using term frequency-inverse document frequency (TF-IDF) transformation using RunTFIDF [41] to identify the top 1000 most variable peaks. For scRNA, we performed normalization and variance stabilization using SCTransform [42] and picked the top 1000 most variable genes. The resulting data includes gene expression and chromatin regions of 8981 for 1000 genes and regions respectively.

#### Peripheral Blood Mononuclear cells

The Peripheral Blood Mononuclear cells (PBMC) dataset [5] was generated by CITE-seq, containing genes and proteins from the same cells. This data contains cells from two experiments performed on PBMCs: 6855 cells from PBMC10k and 3994 cells from PBMC5k. We preprocessed multiome data from two experiments independently. For scRNA-seq, we performed normalization and variance stabilization using SCTransform [42] and picked 2960 highly variable genes. We identified the variable genes in PBMC10k first and used them as a reference to subset genes in PBMC5k scRNA data. For protein expression, we performed centered log-ratio (CLR) normalization using Seurat’s functions in both PBMC10k and PBMC5k. The resulting PBMC10k data dimension was 6855 by 2960 for gene expression and 6855 by 14 for protein expression. The resulting PBMC10k data includes gene and protein expression data of 3994 cells for 2960 genes and 14 proteins.

#### Dexamethasone-treated A549 cells

The dexamethasone (DEX)-treated A549 dataset [2] was generated using a sci-CAR experiment for single cells from the A549 lung adenocarcinoma cell line. The data contains jointly profiled 2641 cells after 0, 1, and 3 h of 100 nM DEX treatment for gene expression and open chromatin regions. We used a preprocessed dataset previously used by Jin et al. [43], and filtered out lowly expressed cells by gene expression. We reduced the dataset to 2391 cells. For scRNA, we used all 1183 genes. For scATAC, we picked the top highly variable 1183 peaks. The resulting data includes gene expression and chromatin regions of 2391 cells for 1183 genes and regions respectively.

#### Pan-cancer cell lines

This dataset contains three cancer cell lines [3]: HCT116, HeLa-S3, and K562, generated by joint profiling of 206 single cells using scCAT-seq containing gene expression and open chromatin regions. We used a preprocessed dataset previously used by Huizing et al. [18], however, we reduced the number of genes and peaks to 10,000 by selecting the most variable features. We binarized the scATAC profile where we set all values greater than 0 to 1 and 0 otherwise. The resulting data includes gene expression and chromatin regions of 206 cells for 10,000 genes and regions respectively.

### Runtime evaluations

We compare CMOT’s running time with state-of-art methods MOFA + , Seurat, TotalVI, and Polarbear for the best-performing parameters used for cross-modality inference (Table S29). We benchmarked all methods on Intel Xeon Gold 6242R CPU @3.10 GHz × 40 with 251.4GiB RAM and NVIDIA RTX A6000 GPU, Hierarchical Clustering.

We induced cell labels for datasets with no prior knowledge (e.g., cell types). We use these labels for label regularization in OT optimization (see Methods and Materials Step B) to improve the mappings between cells in the source ($X$) and target ($Y$) modalities. To induce cell labels, we performed hierarchical clustering of training and validation sets combined using the scikit-learn clustering functions [23].

### Training and cross-validation

We split the human brain [1], PBMCs [5], DEX-A549 lung cancer [2], and pan-cancer [3] datasets into 80% train and 20% test.

We trained all methods: Seurat [7, 8], MOFA + [9], TotalVI [5], and Polarbear [11] using default parameters for all datasets except DEX-treated A549 [2]. For modality inference in Seurat [7, 8], we integrated the training modalities first, and then we inferred the missing modality using FindTransferAnchor and TransferData functions [7] between the integrated training modalities and source test modality. For MOFA + [9], we input the missing modality as NA values and trained the model on the multimodalities. We trained TotalVI [5] autoencoder with default parameters, with latent distribution set to “normal,” on the training set. Finally, we trained Polarbear and Polarbear co-assay models [11] using default parameters on the training set.

To identify the highest performing parameters for Steps B and C of CMOT, we performed 5-fold cross-validation on the training set. We reported the best-performing parameters for each dataset in the Results.

For the DEX-treated A549 dataset, we tuned parameters for all methods (Additional File 1: Fig. S9) and benchmark inference performance of CMOT against state-of-arts (Fig. 4A, Additional File 1: Fig. S4).

### Parameter selection

In Step A, we found the optimal alignment by testing different values of $d$ common manifold dimensions and *K* nearest neighbors for building the similarity within each modality (Additional File 1: Fig. S10). In Step B and Step C, we performed cross-validation to select the regularization coefficients $\lambda$ and $\eta$ for optimal transport, and top highly variable features and *k*-nearest neighbors for modality inference. In particular, we chose the optimal number of features and* k*-nearest neighbors based on CMOT’s performance saturation (Additional File 1: Fig. S11)*.* Also, for all datasets, applied in this paper, we held out a 20% testing set to report CMOT’s performance. For datasets with no prior knowledge (e.g., cell types), we induced cell labels by cell clusters through hierarchical clustering of the training set, when training the final model (see Additional File 1: Supplementary Methods). We split the training data into training and validation sets to select parameters through 5-fold cross-validation (see Additional File 1: Supplementary Methods).

### Evaluation

#### Inference versus measurement

To evaluate CMOT’s inferred gene and protein expressions, we calculated Pearson’s correlation coefficient between the inferred and measured expression values of each cell (cell-wise). Also, we computed the gene-wise correlation between inferred and measured expression values across cells for each gene [11]. For peak inference in open chromatin regions, we used AUROC to evaluate the quality of CMOT’s binarized inferred peaks [11]. We computed peak-wise AUROC between individual inferred peak profiles versus measured profiles. This evaluation also applied to the state-of-art methods that we compared. We reported the number of genes with improved correlation/AUROC w.r.t. these methods along with a one-sided Wilcox rank-sum test *p*-value for each [11].

#### Classifying known cell type using inferred expression

For the human brain data with known brain cell type information, we evaluated the CMOT inferred expression of cell-type marker genes for classifying the cell type and calculated the AUPRC of the classification [11]. To this end, given a cell type, we labeled all cells that belong to the cell type as positive and the rest as negative. Specifically, we evaluated the Top 8 marker genes from each cell type, due to disproportionate cell-type distribution within the dataset, using a total of 80 cells. We then defined a baseline = 0.1 for the AUPRC as the ratio of the number of positives versus total cells.

#### Clustering cancer types using inferred gene expression by silhouette score

For the pan-cancer dataset, we evaluated CMOT to separate the cancer types. In particular, we assessed if CMOT’s inferred gene expression data can cluster the cells and cell clusters corresponding to different cancer types [3], using the silhouette score. The silhouette score $S\left(m\right)$ of a cell $m$ belonging to the cluster ${C}_{M}$ is calculated as:

$$S\left(m\right)=\frac{E\left(m\right)-e\left(m\right)}{max\left(E\left(m\right),e\left(m\right)\right)}$$

where $$E\left(m\right)=\begin{array}{c}min\\ M\ne N\end{array}\frac{\sum d\left(m,n\right)}{\left|{C}_{N}\right|}$$ is the inter-cluster distance defined as the average distance to the closest cluster of cell $m$ except that which it’s a part of (i.e., $n\in {C}_{N}$) and $e\left(m\right)=\frac{1}{\left|{C}_{M}\right|-1}\sum d\left(m,n\right)$ is the intra-cluster distance defined as the average distance to all other cells in the cluster to which it’s a part of (i.e. $n\in {C}_{M},m\ne n$). We calculated the silhouette scores by the Python package Scikit-learn [40].

#### Gene set enrichment analysis

We used Metascape [44] to perform gene set enrichment analysis for the highly predictive genes by CMOT.

#### Comparison with state-of-arts

We compared CMOT with existing state-of-the-art methods, Seurat [7, 8], MOFA + [9], TotalVI [5], Polarbear [11], bindSC [20], and GLUE [21]. First, for the human brain data [1], we benchmarked CMOT against Seurat and MOFA + for the human brain data (Fig. 2); additionally, we also benchmarked CMOT against other state-of-art methods (Additional File 1: Fig. S1). Next, for the CITE-seq data [5], we compared CMOT with Seurat, MOFA + , and TotalVI (Fig. 3, Additional File 1: Fig. S7). We added TotalVI to the comparison since it was specifically designed for CITE-seq datasets. For the DEX-treated A549 dataset [2], we benchmarked CMOT against Seurat and MOFA + (Fig. 4), as well as other state-of-art methods (Additional File 1: Fig. S4). For the pan-cancer dataset [3], we benchmarked Seurat and MOFA + due to the small dataset size. Finally, we benchmarked additional state-of-art methods on the mouse brain dataset [22] (Additional File 1: Fig. S2, Fig. S3).

## Supplementary Information

**Additional file 1. **Supplementary Tables S1-S29, Supplementary Figures S1-S13, Supplementary Methods

**Additional file 2. **Top predictive genes by CMOT in human brain [1] and DEX-treated A549 lung cancer cells [2]

**Additional file 3. **CMOT versus MOFA + inferred genes in DEX-treated A549 lung cancer cells [2]

**Additional file 4. **Review history

## Data Availability

CMOT is implemented as an open-source Python package available at https://github.com/daifengwanglab/CMOT [46], and the latest release is hosted by Zenodo [47] under the GNU General Public License v3.0. The single-cell human brain data was downloaded from https://github.com/GreenleafLab/brainchromatin/blob/main/links.txt [1]. The mouse brain dataset from GLUE was downloaded from http://download.gao-lab.org/GLUE/tutorial/Chen-2019-RNA.h5ad, http://download.gao-lab.org/GLUE/tutorial/Chen-2019-ATAC.h5ad [21], and from https://noble.gs.washington.edu/~ranz0/Polarbear/data/ for Polarbear [11]. The A549 dataset was downloaded from https://github.com/sqjin/scAI/tree/master/data [43]. The preprocessed scCAT-seq pan-cancer dataset was downloaded from https://github.com/cantinilab/OT-scOmics/tree/main/data [18]. The CITE-seq PBMC dataset was downloaded from the scvi-tools website https://docs.scvi-tools.org/en/stable/tutorials/notebooks/totalVI.html [5]. The single-cell developing human brain dataset is available in the Gene Expression Omnibus (GEO) with the accession number GSE162170 [48]. The mouse brain dataset is available in GEO with the accession number GSE126074 [49]. The A549 dataset is available in GEO with the accession numbers GSM3271040 and GSM3271041 [50]. The pan-cancer is available in GEO with the accession number GSE81861 [51]. The PBMC datasets are available at 10 × Genomics (PBMC5K [52]; PBMC10K [53]).
