# Supplementary material for: CMOT: Cross-Modality Optimal Transport for multimodal inference
Source: Genome Biol. 2023 Jul 11;24:163. doi: 10.1186/s13059-023-02989-8 (PMC10334579; doi:10.1186/s13059-023-02989-8)
Supplement: Supplementary file 1 — Additional file 1. Supplementary Tables S1-S29, Supplementary Figures S1-S13, Supplementary Methods [file 13059_2023_2989_MOESM1_ESM.docx]

# Supplementary Tables

| **Methods** | **Mean Pearson Correlation** | **Median Pearson Correlation** |
| --- | --- | --- |
| **CMOT (*p*=100%)** | 0.66 | 0.67 |
| **CMOT (*p*=75%)** | 0.65 | 0.68 |
| **CMOT (*p*=50%)** | 0.63 | 0.65 |
| **CMOT (*p*=25%)** | 0.58 | 0.61 |
| **Seurat** | 0.62 | 0.64 |
| **MOFA+** | 0.43 | 0.41 |
| **GLUE** | 0.46 | 0.47 |
| **bindSC** | 0.63 | 0.68 |
| **Seurat CCA** | 0.66 | 0.68 |

**Table S1: Gene expression inference from chromatin accessibility in human developing brain data [1].** Mean and median cell-wise Pearson correlation between inferred and measured gene expression comparing methods (x-axis) CMOT(*p*=25%,50%,75%,100%), Seurat, MOFA+, GLUE, bindSC, and, Seurat CCA plotted in Fig. S6 for human developing brain [1].

| **Methods** | **Mean Spearman Correlation** | **Median Spearman Correlation** |
| --- | --- | --- |
| **CMOT (*p*=100%)** | 0.44 | 0.44 |
| **CMOT (*p*=75%)** | 0.44 | 0.44 |
| **CMOT (*p*=50%)** | 0.43 | 0.44 |
| **CMOT (*p*=25%)** | 0.43 | 0.43 |
| **Seurat** | 0.45 | 0.45 |
| **MOFA+** | 0.17 | 0.16 |
| **GLUE** | 0.42 | 0.42 |
| **bindSC** | 0.44 | 0.45 |
| **Seurat CCA** | 0.44 | 0.44 |

**Table S2: Gene expression inference from chromatin accessibility in human developing brain data [1].** Wilcox-rank sum test p-values of cell-wise Pearson correlation between inferred and measured gene expression comparing methods (x-axis) CMOT (*p*=25%,50%,75%,100%), Seurat, MOFA+, GLUE, bindSC, and, Seurat CCA plotted in Fig. S6 for human developing brain [1]. All values have been calculated between CMOT variations (*p*=100%,75%,50%,25%) , and competing methods by setting alternative=“greater”.

| **Methods** | **Seurat** | **MOFA+** | **GLUE** | **bindSC** | **Seurat CCA** |
| --- | --- | --- | --- | --- | --- |
| **CMOT (*p*=100%)** | 1.23×10−14 | 0 | 1.4×10−236 | 0.97 | 0.98 |
| **CMOT (*p*=75%)** | 3.4×10−10 | 2.8×10-294 | 1.3×10−216 | 0.99 | 0.99 |
| **CMOT (*p*=50%)** | 0.3 | 3.3×10-240 | 1.5×10−162 | 1 | 1 |
| **CMOT (*p*=25%)** | 1 | 1.65×10-157 | 2.4×10−86 | 1 | 1 |

**Table S3: Gene expression inference from chromatin accessibility in human developing brain data [1].** Wilcox-rank sum test p-values of cell-wise Pearson correlation between inferred and measured gene expression comparing methods (x-axis) CMOT(*p*=25%,50%,75%,100%), Seurat, MOFA+, GLUE, bindSC, and, Seurat CCA plotted in Fig. S6 for human developing brain [1]. All values have been calculated between CMOT variations (*p*=100%,75%,50%,25%), and competing methods by setting alternative=” greater”.

| **Methods** | **Seurat** | **MOFA+** | **GLUE** | **bindSC** | **Seurat CCA** |
| --- | --- | --- | --- | --- | --- |
| **CMOT (*p*=100%)** | 0.93 | 0 | 5.51×10-30 | 0.99 | 0.27 |
| **CMOT (*p*=75%)** | 0.99 | 0 | 6.36×10-21 | 1 | 0.97 |
| **CMOT (*p*=50%)** | 1 | 0 | 4.49×10-12 | 1 | 0.99 |
| **CMOT (*p*=25%)** | 1 | 0 | 2.87×10-05 | 1 | 1 |

**Table S4: Gene expression inference from chromatin accessibility in human developing brain data [1].** Wilcox-rank sum test p-values of cell-wise Spearman correlation between inferred and measured gene expression comparing methods (x-axis) CMOT(*p*=25%,50%,75%,100%), Seurat, MOFA+, GLUE, bindSC, and, Seurat CCA plotted in Table S2 for human developing brain [1]. All values have been calculated between CMOT variations (*p*=100%,75%,50%,25%), and competing methods by setting alternative=” greater”.

| **Methods** | **Mean Pearson Correlation** | **Median Pearson Correlation** |
| --- | --- | --- |
| **CMOT (*p*=100%)** | 0.71 | 0.76 |
| **CMOT (*p*=75%)** | 0.71 | 0.76 |
| **CMOT (*p*=50%)** | 0.71 | 0.76 |
| **CMOT (*p*=25%)** | 0.67 | 0.71 |
| **Seurat** | 0.66 | 0.7 |
| **MOFA+** | 0.69 | 0.74 |
| **GLUE** | 0.67 | 0.72 |
| **bindSC** | 0.68 | 0.72 |
| **Seurat CCA** | 0.7 | 0.75 |

**Table S5: Gene expression inference from chromatin accessibility in mouse brain data [22].** Mean and median cell-wise Pearson correlation between inferred and measured gene expression comparing methods (x-axis) CMOT (*p*=25%,50%,75%,100%), MOFA+, Seurat, GLUE, bindSC, Seurat CCA plotted in Fig. S1 for mouse brain [22].

| **Methods** | **Mean Spearman Correlation** | **Median Spearman Correlation** |
| --- | --- | --- |
| **CMOT (*p*=100%)** | 0.34 | 0.34 |
| **CMOT (*p*=75%)** | 0.34 | 0.34 |
| **CMOT (*p*=50%)** | 0.35 | 0.35 |
| **CMOT (*p*=25%)** | 0.35 | 0.35 |
| **Seurat** | 0.4 | 0.4 |
| **MOFA+** | 0.31 | 0.32 |
| **GLUE** | 0.32 | 0.33 |
| **bindSC** | 0.44 | 0.46 |
| **Seurat CCA** | 0.36 | 0.36 |

**Table S6: Gene expression inference from chromatin accessibility in mouse brain data[22].** Wilcox-rank sum test p-values of cell-wise Spearman correlation between inferred and measured gene expression comparing methods (x-axis) CMOT (*p*=25%,50%,75%,100%), and, MOFA+ plotted in Fig. S1 for mouse brain [22]. All values have been calculated between CMOT variations (*p*=100%,75%,50%,25%) , and competing methods by setting alternative=“greater”.

| **Methods** | **Seurat** | **MOFA+** | **GLUE** | **bindSC** | **Seurat CCA** |
| --- | --- | --- | --- | --- | --- |
| **CMOT (*p*=100%)** | 3.4×10-40 | 1.15×10-05 | 5.6×10-25 | 8×10-19 | 1.9×10-04 |
| **CMOT (*p*=75%)** | 1.25×10-39 | 1.67×10-05 | 1.63×10-24 | 1.8×10-18 | 2.8×10-4 |
| **CMOT (*p*=50%)** | 1.34×10-35 | 8×10-04 | 5.04×10-21 | 2.06×10-15 | 8×10-03 |
| **CMOT (*p*=25%)** | 0.07 | 1 | 0.9 | 0.99 | 1 |

**Table S7: Gene expression inference from chromatin accessibility in mouse brain data [22].** Wilcox-rank sum test p-values of cell-wise Pearson correlation between inferred and measured gene expression comparing methods (x-axis) CMOT (*p*=25%,50%,75%,100%), and, MOFA+ plotted in Fig. S1 for mouse brain [22]. All values have been calculated between CMOT variations (*p*=100%,75%,50%,25%), and competing methods by setting alternative=“greater”.

| **Methods** | **Seurat** | **MOFA+** | **GLUE** | **bindSC** | **Seurat CCA** |
| --- | --- | --- | --- | --- | --- |
| **CMOT (*p*=100%)** | 1 | 5×10-28 | 6.1×10-13 | 1 | 1 |
| **CMOT (*p*=75%)** | 1 | 4.72×10-30 | 2.04×10-14 | 1 | 1 |
| **CMOT (*p*=50%)** | 1 | 5×10-37 | 1.64×10-19 | 1 | 1 |
| **CMOT (*p*=25%)** | 1 | 1.47×10-55 | 3×10-34 | 1 | 1 |

**Table S8: Gene expression inference from chromatin accessibility in mouse brain data [22].** Wilcox-rank sum test p-values of cell-wise Spearman correlation between inferred and measured gene expression comparing methods (x-axis) CMOT (*p*=25%,50%,75%,100%), and, MOFA+ plotted in Fig. S1 for mouse brain [22]. All values have been calculated between CMOT variations (*p*=100%,75%,50%,25%), and competing methods by setting alternative=“greater”.

| **Methods** | **Mean Pearson Correlation** | **Median Pearson Correlation** |
| --- | --- | --- |
| **CMOT (*p*=100%)** | 0.67 | 0.71 |
| **CMOT (*p*=75%)** | 0.67 | 0.71 |
| **CMOT (*p*=50%)** | 0.66 | 0.7 |
| **CMOT (*p*=25%)** | 0.64 | 0.68 |
| **Seurat** | 0.6 | 0.63 |
| **MOFA+** | 0.68 | 0.71 |
| **Polarbear** | 0.68 | 0.71 |
| **Polarbear-coassay** | 0.67 | 0.71 |

**Table S9: Gene expression inference from chromatin accessibility in mouse brain [22].** Mean and median cell-wise Pearson correlation between inferred and measured gene expression comparing methods (x-axis) CMOT (*p*=25%,50%,75%,100%), MOFA+, Seurat, and, Polarbear plotted in Fig. S12 for mouse brain [22].

| **Methods** | **Seurat** | **MOFA+** | **Polarbear** | **Polarbear-coassay** |
| --- | --- | --- | --- | --- |
| **CMOT (*p*=100%)** | 2.97×10-69 | 0.7 | 0.7 | 0.29 |
| **CMOT (*p*=75%)** | 2.97×10-69 | 0.7 | 0.7 | 0.29 |
| **CMOT (*p*=50%)** | 1.51×10-51 | 0.9 | 0.9 | 0.9 |
| **CMOT (*p*=25%)** | 9.05×10-26 | 1 | 1 | 1 |

**Table S10: Gene expression inference from chromatin accessibility in mouse brain [22].** Wilcox-rank sum test p-values of cell-wise Spearman correlation between inferred and measured gene expression comparing methods (x-axis) CMOT (*p*=25%,50%,75%,100%), Polarbear, and, MOFA+ plotted in Fig. S12 for mouse brain [22]. All values have been calculated between CMOT variations (*p*=100%,75%,50%,25%), and competing methods by setting alternative=“greater”.

| **Methods** | **Mean Pearson Correlation** | **Median Pearson Correlation** |
| --- | --- | --- |
| **CMOT (*p*=100%)** | 0.78 | 0.86 |
| **CMOT (*p*=75%)** | 0.78 | 0.86 |
| **CMOT (*p*=50%)** | 0.77 | 0.85 |
| **CMOT (*p*=25%)** | 0.74 | 0.83 |
| **Seurat** | 0.84 | 0.85 |
| **MOFA+** | 0.75 | 0.79 |
| **TotalVI** | 0.61 | 0.61 |

**Table S11: Protein expression inference from gene expression in PBMC [5].** Mean and median cell-wise Pearson correlation between inferred and measured gene expression comparing methods (x-axis) CMOT(*p*=25%,50%,75%,100%), Seurat, MOFA+, TotalVI plotted in Fig. 3A for PBMC [5].

| **Methods** | **Seurat** | **MOFA+** | **TotalVI** |
| --- | --- | --- | --- |
| **CMOT (*p*=100%)** | 1 | 6.9×10−57 | 0 |
| **CMOT (*p*=75%)** | 1 | 0 | 8.36×10−58 |
| **CMOT (*p*=50%)** | 1 | 0 | 1.73×10−45 |
| **CMOT (*p*=25%)** | 1 | 0 | 5.25×10−12 |

**Table S12: Protein expression inference from gene expression in PBMC [5].** Wilcox-rank sum test p-values of cell-wise Pearson correlation between inferred and measured gene expression comparing methods (x-axis) CMOT(*p*=25%,50%,75%,100%), Seurat, and, MOFA+ plotted in Fig. 3A for Peripheral Blood Mononuclear Cells (PBMCs) [5]. All values have been calculated between CMOT variations (*p*=100%,75%,50%,25%), and competing methods by setting alternative=” greater”.

| **Protein** | **Pearson Correlation** | **P-value** | **Spearman Correlation** | **P-value** |
| --- | --- | --- | --- | --- |
| **CD3** | 0.92 | 0 | 0.79 | 0 |
| **CD4** | 0.83 | 0 | 0.76 | 0 |
| **CD8a** | 0.54 | 3.1×10−312 | 0.018 | 0.25 |
| **CD14** | 0.95 | 0 | 0.67 | 0 |
| **CD15** | 0.5 | 1.58×10−252 | 0.46 | 1.18×10-218 |
| **CD16** | 0.75 | 0 | 0.4 | 3.98×10-153 |
| **CD56** | 0.69 | 0 | 0.34 | 2.16×10-114 |
| **CD19** | 0.86 | 0 | 0.23 | 1.92×10-50 |
| **CD25** | 0.4 | 3.06×10−144 | 0.39 | 2.07×10-145 |
| **CD45RA** | 0.72 | 0 | 0.72 | 0 |
| **CD45RO** | 0.73 | 0 | 0.7 | 0 |
| **PD-1** | 0.31 | 2.36×10−89 | 0.26 | 8.95×10-65 |
| **TIGIT** | 0.45 | 6.17×10−197 | 0.34 | 9.29×10-112 |
| **CD127** | 0.85 | 0 | 0.71 | 0 |

**Table S13: Pearson correlation between inferred and measured protein expression in PBMC [5]:** Pearson and Spearman correlations, and, p-values between CMOT’s inferred and measured protein expression for PBMCs 5K [5] (Fig. 3B, 3C) (independent evaluation).

| **Methods** | **Mean Pearson Correlation** | **Median Pearson Correlation** |
| --- | --- | --- |
| **CMOT (*p*=100%)** | 0.83 | 0.91 |
| **CMOT (*p*=75%)** | 0.83 | 0.9 |
| **CMOT (*p*=50%)** | 0.82 | 0.9 |
| **CMOT (*p*=25%)** | 0.8 | 0.88 |
| **Seurat** | 0.87 | 0.92 |
| **MOFA+** | 0.83 | 0.89 |
| **TotalVI** | 0.01 | -.0.08 |

**Table S14: Protein expression inference from gene expression in PBMC [5].** Mean and median cell-wise Pearson correlation between inferred and measured gene expression comparing methods (x-axis) CMOT(*p*=25%,50%,75%,100%), Seurat, MOFA+, TotalVI plotted in Fig. S8 for PBMC [5].

| **Methods** | **Seurat** | **MOFA+** | **TotalVI** |
| --- | --- | --- | --- |
| **CMOT (*p*=100%)** | 0.99 | 1.2×10−05 | 0 |
| **CMOT (*p*=75%)** | 1 | 1.4×10−2 | 0 |
| **CMOT (*p*=50%)** | 1 | 0.13 | 0 |
| **CMOT (*p*=25%)** | 1 | 0.99 | 0 |

**Table S15: Protein expression inference from gene expression in PBMC [5].** Wilcox-rank sum test p-values of cell-wise Pearson correlation between inferred and measured gene expression comparing methods (x-axis) CMOT(*p*=25%,50%,75%,100%), Seurat, and, MOFA+ plotted in Fig. S8 for Peripheral Blood Mononuclear Cells (PBMCs) [5]. All values have been calculated between CMOT variations (*p*=100%,75%,50%,25%), and competing methods by setting alternative=” greater”.

| **Protein** | **Pearson Correlation** | **P-value** | **Spearman Correlation** | **P-value** |
| --- | --- | --- | --- | --- |
| **CD3** | 0.95 | 0 | 0.8 | 5.5×10-317 |
| **CD4** | 0.9 | 0 | 0.86 | 0 |
| **CD8a** | 0.82 | 0 | 0.4 | 4.1×10-52 |
| **CD14** | 0.96 | 0 | 0.55 | 1.6×10-111 |
| **CD15** | 0.56 | 1×10-118 | 0.65 | 8.8×10-169 |
| **CD16** | 0.79 | 2×10-302 | 0.54 | 7.5×10-107 |
| **CD56** | 0.84 | 0 | 0.57 | 1.2×10-122 |
| **CD19** | 0.95 | 0 | 0.18 | 1.34×10-11 |
| **CD25** | 0.5 | 7.7×10-90 | 0.51 | 2.25×10-95 |
| **CD45RA** | 0.83 | 0 | 0.83 | 0 |
| **CD45RO** | 0.8 | 5.9×10-306 | 0.76 | 3.6×10-262 |
| **PD-1** | 0.43 | 9.6×10-66 | 0.32 | 7.28×10-35 |
| **TIGIT** | 0.72 | 5.7×10-222 | 0.47 | 9.8×10-78 |
| **CD127** | 0.85 | 2.2×10-16 | 0.8 | 8.6×10-316 |

**Table S16: Pearson correlation between inferred and measured protein expression in PBMC [5]:** Pearson correlation and p-values between CMOT’s inferred and measured protein expression for PBMCs 10K [5] (Fig. S8)

| **Methods** | **Mean Pearson Correlation** | **Median Pearson Correlation** |
| --- | --- | --- |
| **CMOT (*p*=100%)** | 0.52 | 0.52 |
| **CMOT (*p*=75%)** | 0.51 | 0.52 |
| **CMOT (*p*=50%)** | 0.5 | 0.51 |
| **CMOT (*p*=25%)** | 0.49 | 0.5 |
| **Seurat** | 0.49 | 0.5 |
| **MOFA+** | 0.52 | 0.52 |
| **GLUE** | 0.49 | 0.5 |
| **bindSC** | 0.40 | 0.51 |
| **Seurat CCA** | 0.5 | 0.51 |

**Table S17: Gene expression inference from chromatin accessibility in DEX-treat A549 lung cancer data [2].** Mean and median cell-wise Pearson correlation between inferred and measured gene expression comparing methods (x-axis) CMOT (*p*=25%,50%,75%,100%), Seurat, MOFA+, GLUE, bindSC, and, Seurat CCA plotted in Fig. S7 for DEX-treated A549 cells [2].

| **Methods** | **Mean Spearman Correlation** | **Median Spearman Correlation** |
| --- | --- | --- |
| **CMOT (*p*=100%)** | 0.43 | 0.43 |
| **CMOT (*p*=75%)** | 0.43 | 0.43 |
| **CMOT (*p*=50%)** | 0.43 | 0.43 |
| **CMOT (*p*=25%)** | 0.42 | 0.42 |
| **Seurat** | 0.41 | 0.41 |
| **MOFA+** | 0.44 | 0.44 |
| **GLUE** | 0.43 | 0.44 |
| **bindSC** | 0.39 | 0.49 |
| **Seurat CCA** | 0.42 | 0.42 |

**Table S18: Gene expression inference from chromatin accessibility in DEX-treat A549 lung cancer data [2].** Wilcox-rank sum test p-values of cell-wise Spearman correlation between inferred and measured gene expression comparing methods (x-axis) CMOT (*p*=25%,50%,75%,100%), Seurat, MOFA+, GLUE, bindSC, and, Seurat CCA plotted in Fig. S7 for DEX-treated A549 cells [2]. All values have been calculated between CMOT variations (*p*=100%,75%,50%,25%), and competing methods by setting alternative=“greater”.

| **Methods** | **Seurat** | **MOFA+** | **GLUE** | **bindSC** | **Seurat CCA** |
| --- | --- | --- | --- | --- | --- |
| **CMOT (*p*=100%)** | 1.27×10-05 | 0.64 | 4.7×10−06 | 0.016 | 0.016 |
| **CMOT (*p*=75%)** | 3.9×10-05 | 0.74 | 1.6×10−05 | 0.025 | 0.029 |
| **CMOT (*p*=50%)** | 0.012 | 0.99 | 0.007 | 0.22 | 0.45 |
| **CMOT (*p*=25%)** | 0.7 | 0.99 | 0.66 | 0.82 | 0.99 |

**Table S19: Gene expression inference from chromatin accessibility in DEX-treat A549 lung cancer data [2].** Wilcox-rank sum test p-values of cell-wise Pearson correlation between inferred and measured gene expression comparing methods (x-axis) CMOT (*p*=25%,50%,75%,100%), Seurat, MOFA+, GLUE, bindSC, and, Seurat CCA plotted in Fig. S7 for DEX-treated A549 cells [2]. All values have been calculated between CMOT variations (*p*=100%,75%,50%,25%), and competing methods by setting alternative=“greater”.

| **Methods** | **Seurat** | **MOFA+** | **GLUE** | **bindSC** | **Seurat CCA** |
| --- | --- | --- | --- | --- | --- |
| **CMOT (*p*=100%)** | 1.29×10−06 | 0.61 | 0.44 | 0.9 | 0.005 |
| **CMOT (*p*=75%)** | 5.73×10−06 | 0.73 | 0.57 | 0.9 | 0.012 |
| **CMOT (*p*=50%)** | 5.7×10−04 | 0.96 | 0.91 | 1 | 0.14 |
| **CMOT (*p*=25%)** | 0.06 | 0.99 | 0.99 | 1 | 0.74 |

**Table S20: Gene expression inference from chromatin accessibility in DEX-treat A549 lung cancer data [2].** Wilcox-rank sum test p-values of cell-wise Spearman correlation between inferred and measured gene expression comparing methods (x-axis) CMOT (*p*=25%,50%,75%,100%), Seurat, MOFA+, GLUE, bindSC, and, Seurat CCA for Table S8 for DEX-treated A549 cells [2]. All values have been calculated between CMOT variations (*p*=100%,75%,50%,25%), and competing methods by setting alternative=“greater”.

| **Methods (m)** | **#genes (CMOT>m)** | **#genes(CMOT<m)** | **P-value (CMOT>m)** |
| --- | --- | --- | --- |
| **MOFA+** | 636 | 547 | 2.19×10−06 |
| **Seurat** | 435 | 748 | 1 |

**Table S21: Gene-wise correlation between inferred and measured gene expression in DEX-treat A549 lung cancer data [2].** Number of genes with higher gene-wise Pearson correlation between inferred and measured gene expression profiles for DEX-treated A549 lung adenocarcinoma [2] cells. Column 1 reports the number of genes inferred by CMOT that have a higher correlation than the respective method m. Column 2 reports vice versa of column 1. Column 3 reports the p-value statistic for the Wilcox-rank sum test between gene-wise correlations of CMOT and competing methods (alternative=“greater”).

| **Methods** | **Mean Pearson Correlation** | **Median Pearson Correlation** |
| --- | --- | --- |
| **CMOT (*p*=100%)** | 0.67 | 0.69 |
| **CMOT (*p*=75%)** | 0.66 | 0.68 |
| **CMOT (*p*=50%)** | 0.62 | 0.63 |
| **CMOT (*p*=25%)** | 0.60 | 0.63 |
| **Seurat** | 0.63 | 0.65 |
| **MOFA+** | 0.47 | 0.55 |

**Table S22: Gene expression inference from chromatin accessibility in pan-cancer data [3].** Mean and median cell-wise Pearson correlation between inferred and measured gene expression comparing methods (x-axis) CMOT (*p*=25%,50%,75%,100%), Seurat, MOFA+ plotted in Fig. 5A for pan-cancer data [3].

| **Methods** | **Mean Spearman Correlation** | **Median Spearman Correlation** |
| --- | --- | --- |
| **CMOT (*p*=100%)** | 0.69 | 0.71 |
| **CMOT (*p*=75%)** | 0.67 | 0.69 |
| **CMOT (*p*=50%)** | 0.65 | 0.68 |
| **CMOT (*p*=25%)** | 0.63 | 0.66 |
| **Seurat** | 0.64 | 0.67 |
| **MOFA+** | 0.5 | 0.56 |

**Table S23: Gene expression inference from chromatin** **accessibility in pan-cancer data [3].** Mean and median cell-wise Spearman correlation between inferred and measured gene expression comparing methods (x-axis) CMOT (*p*=25%,50%,75%,100%), Seurat, MOFA+ for pan-cancer data [3].

| **Methods** | **Seurat** | **MOFA+** |
| --- | --- | --- |
| **CMOT (*p*=100%)** | 7.4×10-4 | 2.5×10-06 |
| **CMOT (*p*=75%)** | 0.011 | 4.5×10-05 |
| **CMOT (*p*=50%)** | 0.83 | 0.002 |
| **CMOT (*p*=25%)** | 0.98 | 0.017 |

**Table S24: Gene expression inference from chromatin accessibility in pan-cancer data [3].** Wilcox-rank sum test p-values of cell-wise Pearson correlation between inferred and measured gene expression comparing methods (x-axis) CMOT (*p*=25%,50%,75%,100%), Seurat, and MOFA+ plotted in Fig. 5A, Table S22 for pan-cancer cells [3]. All values have been calculated between CMOT variations (*p*=100%,75%,50%,25%) , and competing methods by setting alternative=“greater”.

| **Methods** | **Seurat** | **MOFA+** |
| --- | --- | --- |
| **CMOT (*p*=100%)** | 0.006 | 1.5×10-05 |
| **CMOT (*p*=75%)** | 0.033 | 8.6×10-05 |
| **CMOT (*p*=50%)** | 0.5 | 0.002 |
| **CMOT (*p*=25%)** | 0.8 | 0.01 |

**Table S25: Gene expression inference from chromatin accessibility in pan-cancer data [3].** Wilcox-rank sum test p-values of cell-wise Spearman correlation between inferred and measured gene expression comparing methods (x-axis) CMOT (*p*=25%,50%,75%,100%), Seurat, and, MOFA+ plotted in Fig. 5A, in Table S23 for pan-cancer cells [3]. All values have been calculated between CMOT variations (*p*=100%,75%,50%,25%) , and competing methods by setting alternative=“greater”.

| **Methods** | **Seurat** | **MOFA+** | **Measured Gene Expression** | **Measured Chromatin Peaks** |
| --- | --- | --- | --- | --- |
| **CMOT** | 1.03×10-05 | 1.6×10-10 | 1.5×10-18 | 5.38×10-18 |

**Table S26: Silhouette score across measured and inferred gene expression in pan-cancer data [3].** Wilcox-rank sum test p-values for Silhouette scores reported in Fig. 5B for Pan-cancer cells [3]. All p-values have been calculated between the silhouette scores reported for inferred gene expressions by CMOT (*p*=100%), Seurat, MOFA+, measured gene expression, and, measured chromatin peaks by setting alternative=“greater”.

| **Methods** | **Mean Pearson Correlation** | **Median Pearson Correlation** |
| --- | --- | --- |
| **CMOT (*p*=100%)** | 0.35 | 0.29 |
| **CMOT (*p*=75%)** | 0.31 | 0.26 |
| **CMOT (*p*=50%)** | 0.30 | 0.23 |
| **CMOT (*p*=25%)** | 0.28 | 0.21 |
| **Seurat** | 0.41 | 0.37 |
| **MOFA+** | -0.03 | -0.03 |

**Table S27: chromatin accessibility inference from gene expression in pan-cancer data [3].** Wilcox-rank sum test p-values of cell-wise Pearson correlation between inferred and measured gene expression comparing methods (x-axis) CMOT(*p*=25%,50%,75%,100%), Seurat, and, MOFA+ plotted in Fig. S4 for pan-cancer data [3]. All values have been calculated between CMOT variations (*p*=100%,75%,50%,25%), and competing methods by setting alternative=” greater”.

| **Methods** | **Seurat** | **MOFA+** |
| --- | --- | --- |
| **CMOT (*p*=100%)** | 0.18 | 0.73 |
| **CMOT (*p*=75%)** | 0.09 | 0.59 |
| **CMOT (*p*=50%)** | 0.32 | 0.8 |
| **CMOT (*p*=25%)** | 0.99 | 0.99 |

**Table S28: chromatin accessibility inference from gene expression in pan-cancer data [3].** Wilcox-rank sum test p-values of cell-wise Pearson correlation between inferred and measured gene expression comparing methods (x-axis) CMOT (*p*=25%,50%,75%,100%), Seurat, and, MOFA+ plotted in Fig. S4 for pan-cancer cells [3]. All values have been calculated between CMOT variations (*p*=100%,75%,50%,25%), and competing methods by setting alternative=“greater”.

| **Methods** | **Developing Human Brain[1]** | **Mouse Brain**  **[22]** | **PBMCs**  **[5]** | **DEX-**  **treated A549 [2]** | **Liu Cancer cell**  **lines [3]**  **(gene expression**  **from chromatin accessibility)** | **Liu Cancer cell**  **lines [3]**  **(chromatin accessibility from**  **gene expression )** |
| --- | --- | --- | --- | --- | --- | --- |
| **CMOT** | **22.62** | **19.56** | **84.14** | **2.93** | **0.96** | **0.73** |
| **Seurat** | 49.74 | - | 347.05 | 18.35 | 11.46 | 13.61 |
| **MOFA+** | 264.13 | - | 1953.2 | 272.3 | 226.54 | 164.91 |
| **TotalVI** | - | - | 426.36 | - | - | - |
| **Polarbear** | - | 964 | - | - | - | - |
| **Polarbear-coassay** | - | 292 | - | - | - | - |
| **scGLUE** | 844.12 | 1011 | - | 710.79 | - | - |
| **bindSC** | 951.3 | 2873 | - | 19.82 | - | - |
| **Seurat CCA** | 122.94 | 491.13 | - | 10.13 | - | - |

**Table S29**: **Running times (in seconds) of all methods across datasets.** (See Additional File 1: supplementary Runtime evaluations)

# Supplementary Figures

| 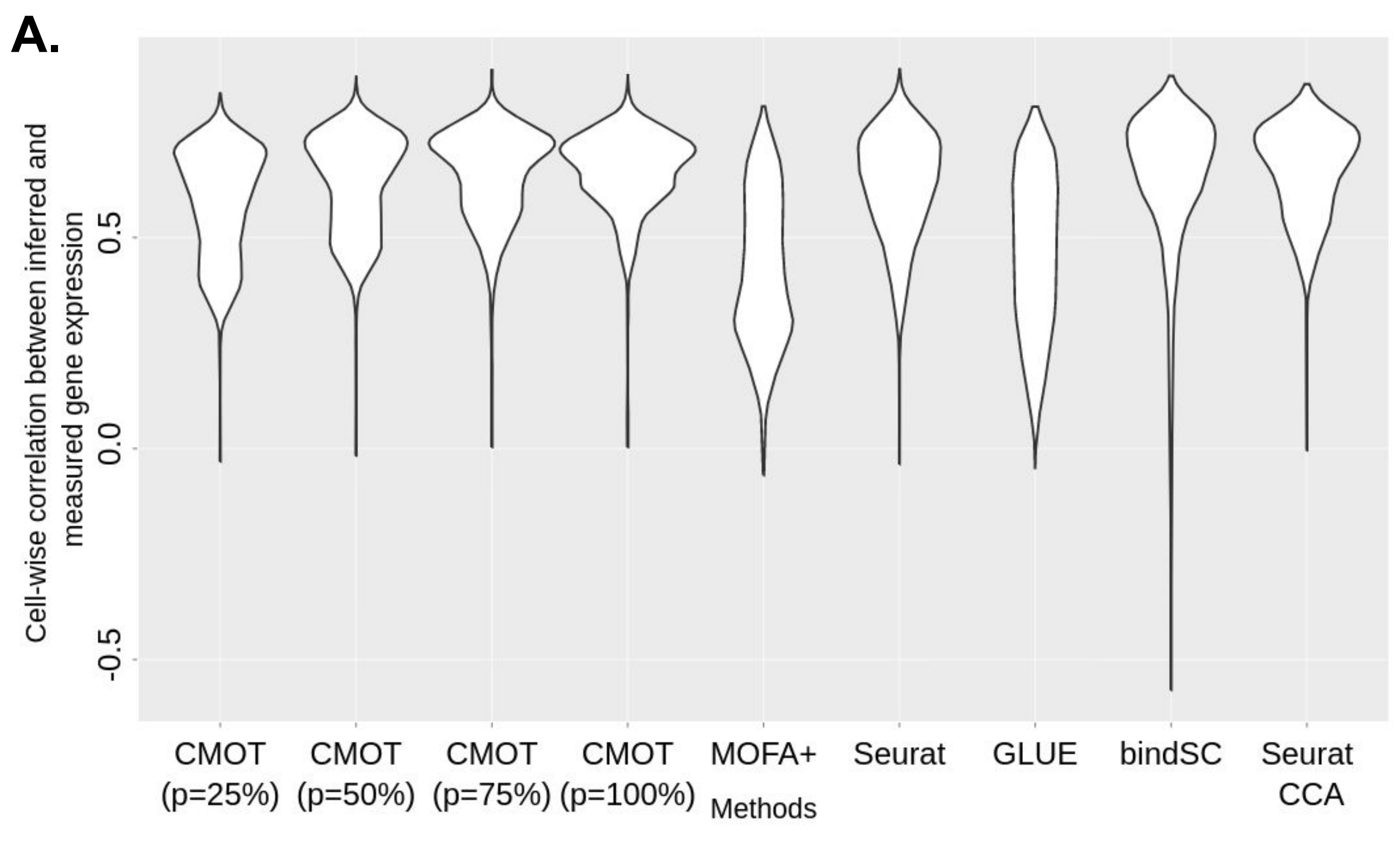 |
| --- |
| **Figure S1:** **Gene expression inference from chromatin accessibility in human developing brain data [1] (A)** Cell-wise Pearson correlation (y-axis) of inferred and measured chromatin accessibility by different methods (x-axis): CMOT (*p*=25%,50%,75%,100%), Seurat, MOFA+, GLUE, bindSC, Seurat CCA (**Tables S1-S4**). |

| 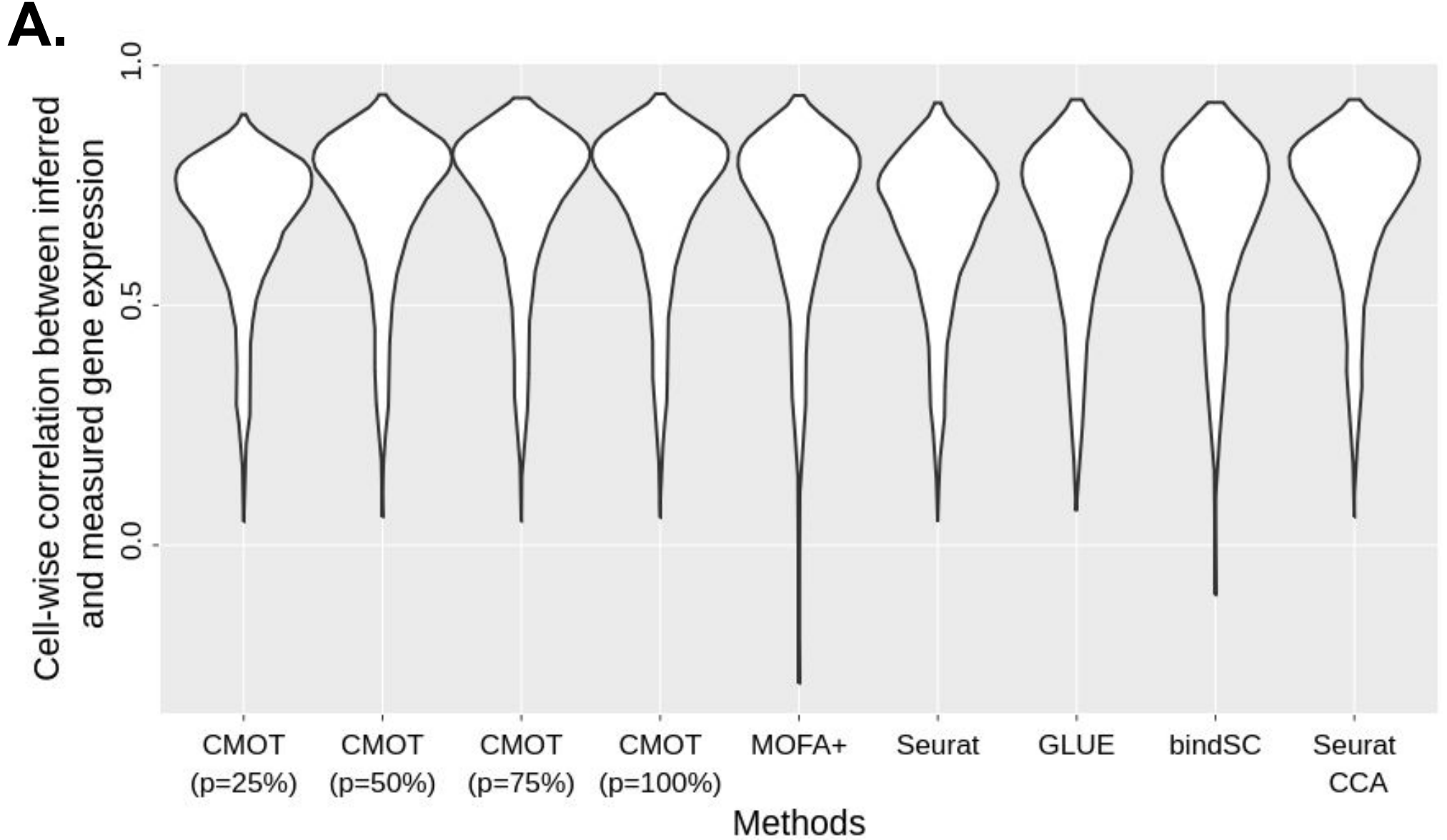 |
| --- |
| **Figure S2:** **Gene expression inference from chromatin accessibility in mouse brain data [22] (A)** Cell-wise Pearson correlation (y-axis) of inferred and measured gene expression by different methods (x-axis): CMOT(*p*=25%,50%,75%,100%), Seurat, MOFA+, GLUE, bindSC, and, Seurat CCA (**Table S5-S8**) |

| 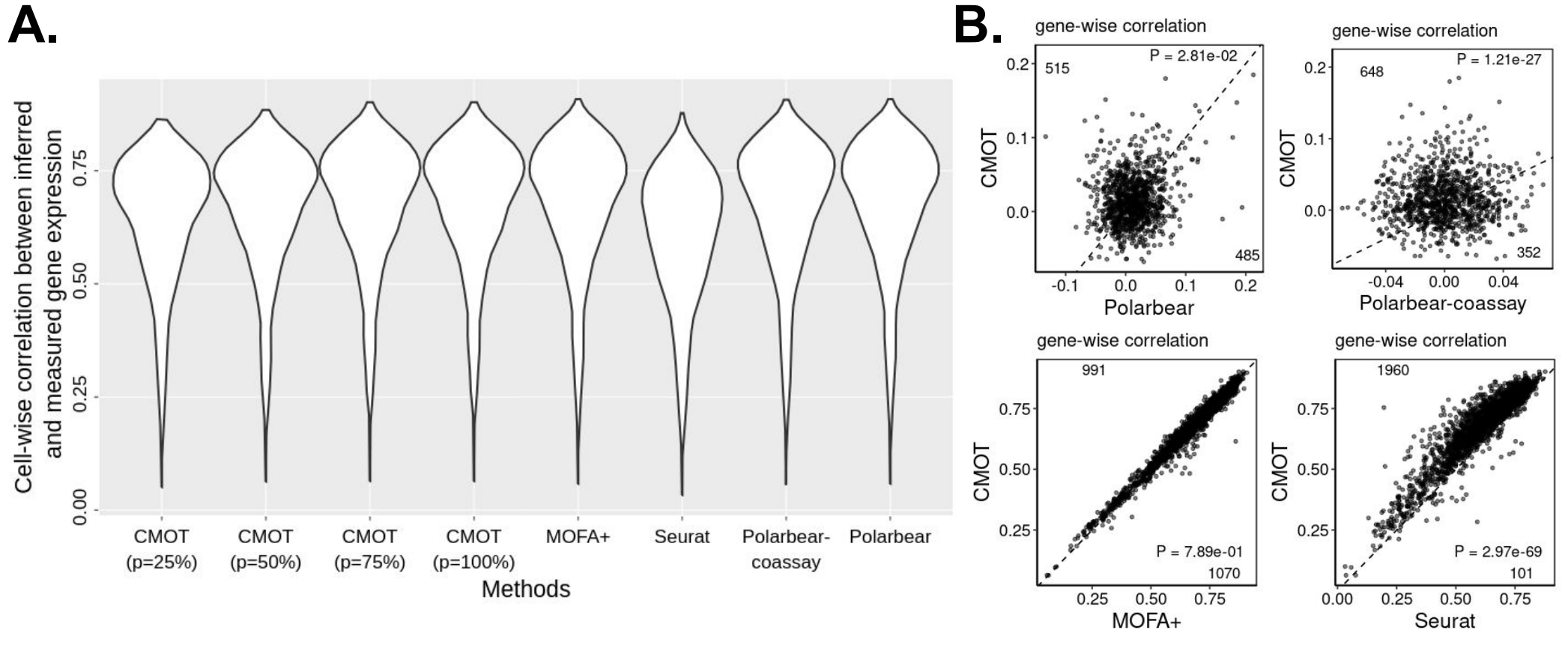 |
| --- |
| **Figure S3:** **Gene expression inference from chromatin accessibility in mouse brain data [22] (A)** Cell-wise Pearson correlation (y-axis) of inferred and measured gene expression by different methods (x-axis): CMOT(*p*=25%,50%,75%,100%), Seurat, MOFA+, Polarbear, and, Polarbear-coassay (**Table S9-S10**) **(B)** Gene-wise correlation between the inferred and measured expression, comparing CMOT (y-axis) with Polarbear, Polarbear-coassay, MOFA+, and, Seurat (x-axis). Dots: Genes; Numbers: Gene numbers above and below the dotted line. |

| 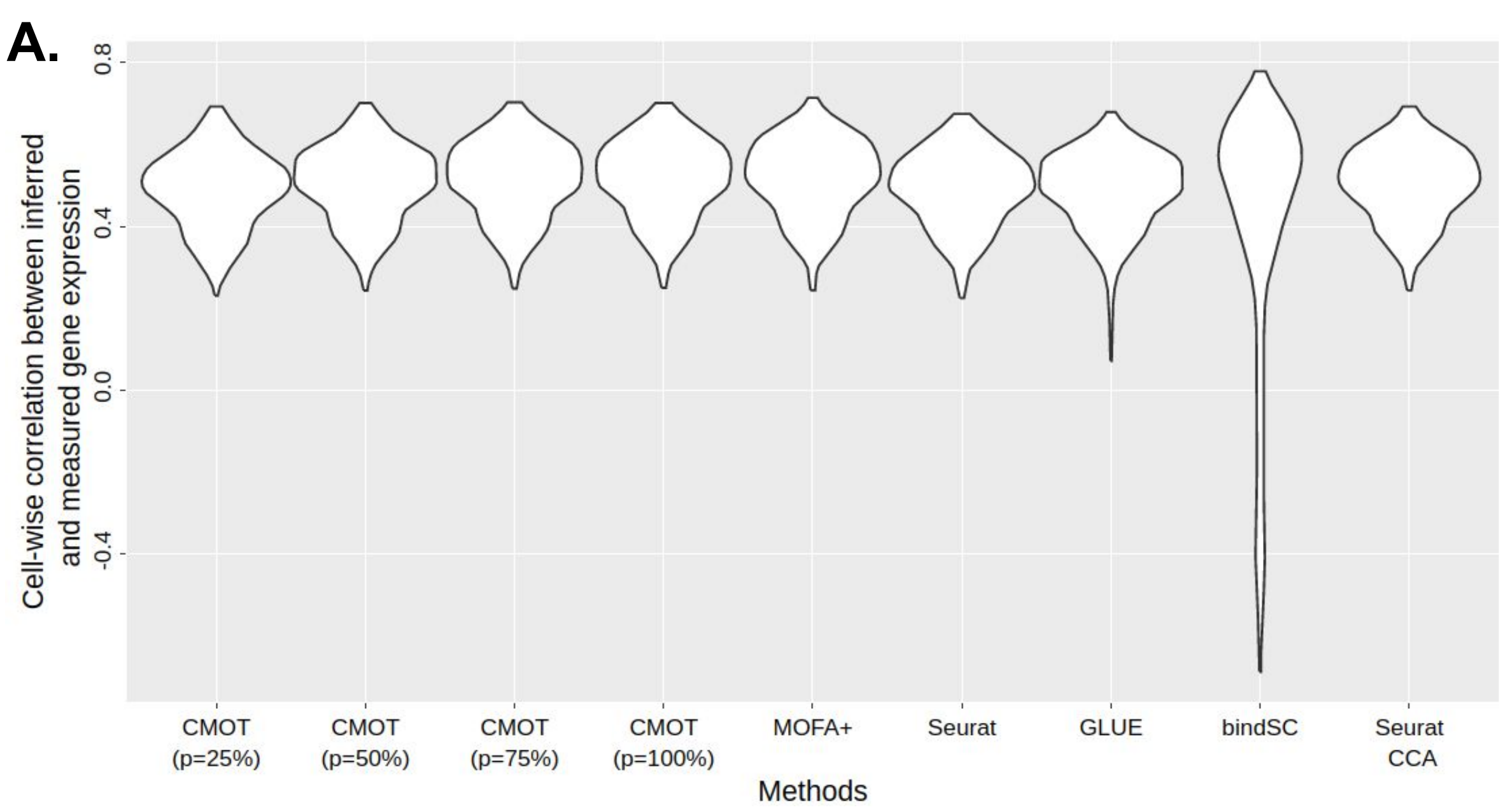 |
| --- |
| **Figure S4:** **Gene expression inference from chromatin accessibility in DEX-treated A549 [2] (A)** Cell-wise Pearson correlation (y-axis) of inferred and measured chromatin accessibility by different methods (x-axis): CMOT (*p*=25%,50%,75%,100%), MOFA+, Seurat, GLUE, bindSC, Seurat CCA (**Table S17-S20**) [2]. |

| 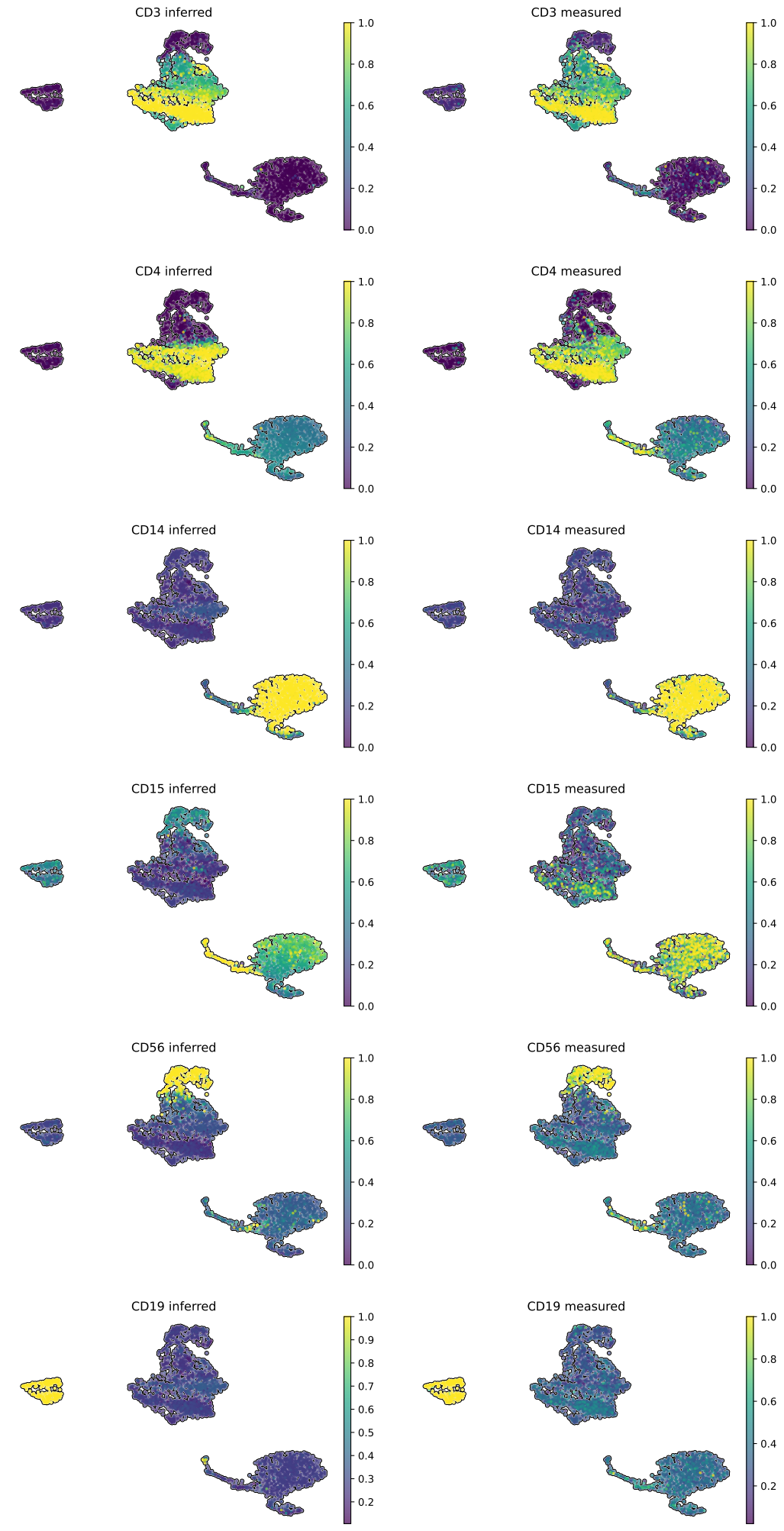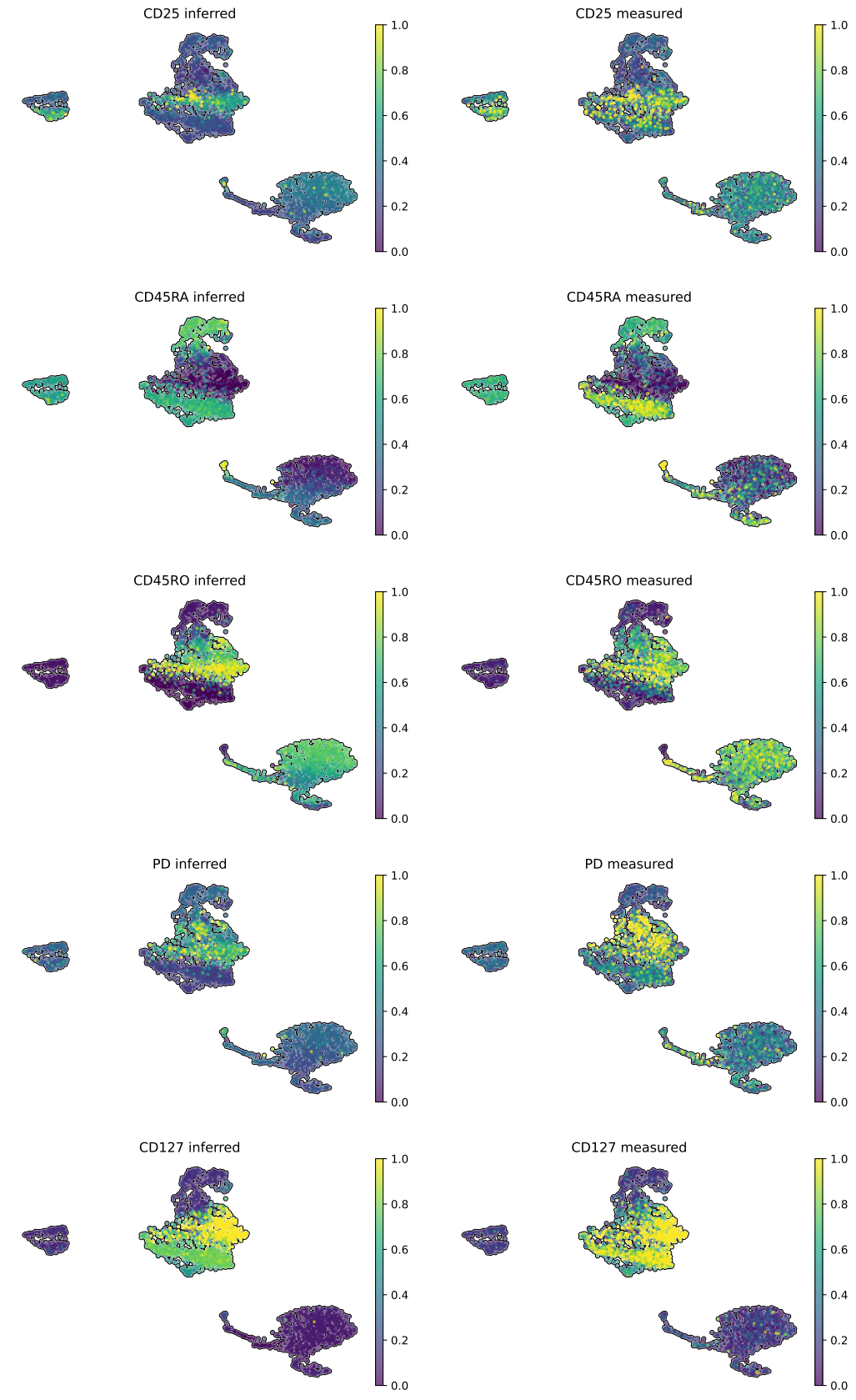 |
| --- |
| **Figure S5: Inferring protein expression from RNA in single-cell peripheral blood mononuclear cells [5].** Inferred versus Measured proteins expressions for Peripheral Blood Mononuclear Cells (PBMCs) [5] by CMOT. |

| 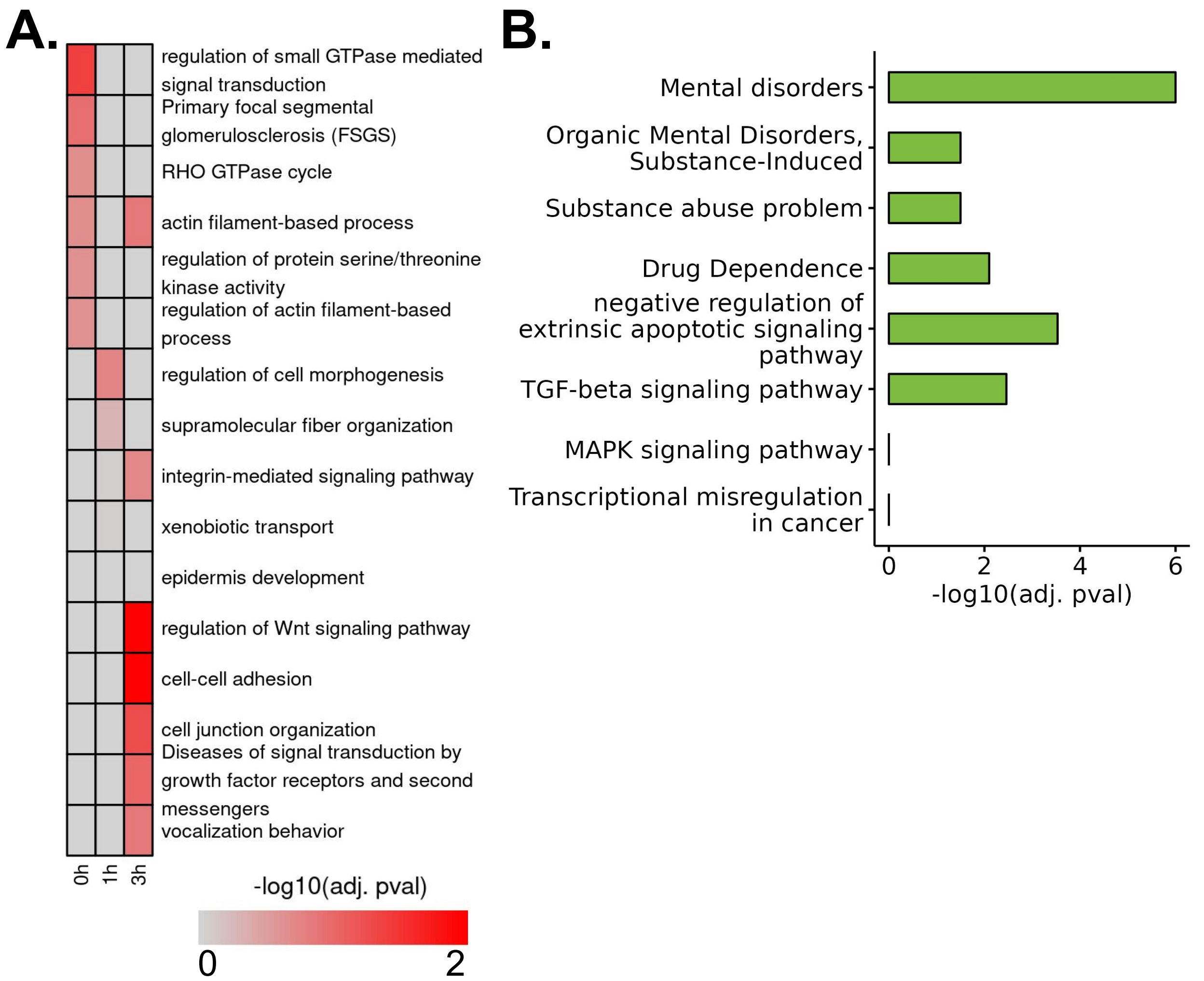 |
| --- |
| **Figure S6: Inference of gene expression for DEX-treated A549 lung cancer cells [2] using chromatin accessibility. (A)** Heatmap showing different enriched terms ranked by -log10(adj. p-value of enrichment) values for the top 100 highly predictive genes within each treatment hour (see Methods, Additional File 2) **(B)** Enriched terms associated with MOFA+ inferred gene expression using 748 genes with a higher gene-wise Pearson correlation compared to CMOT’s 435 genes inference in (**Fig. 4B, 4D**). |

| 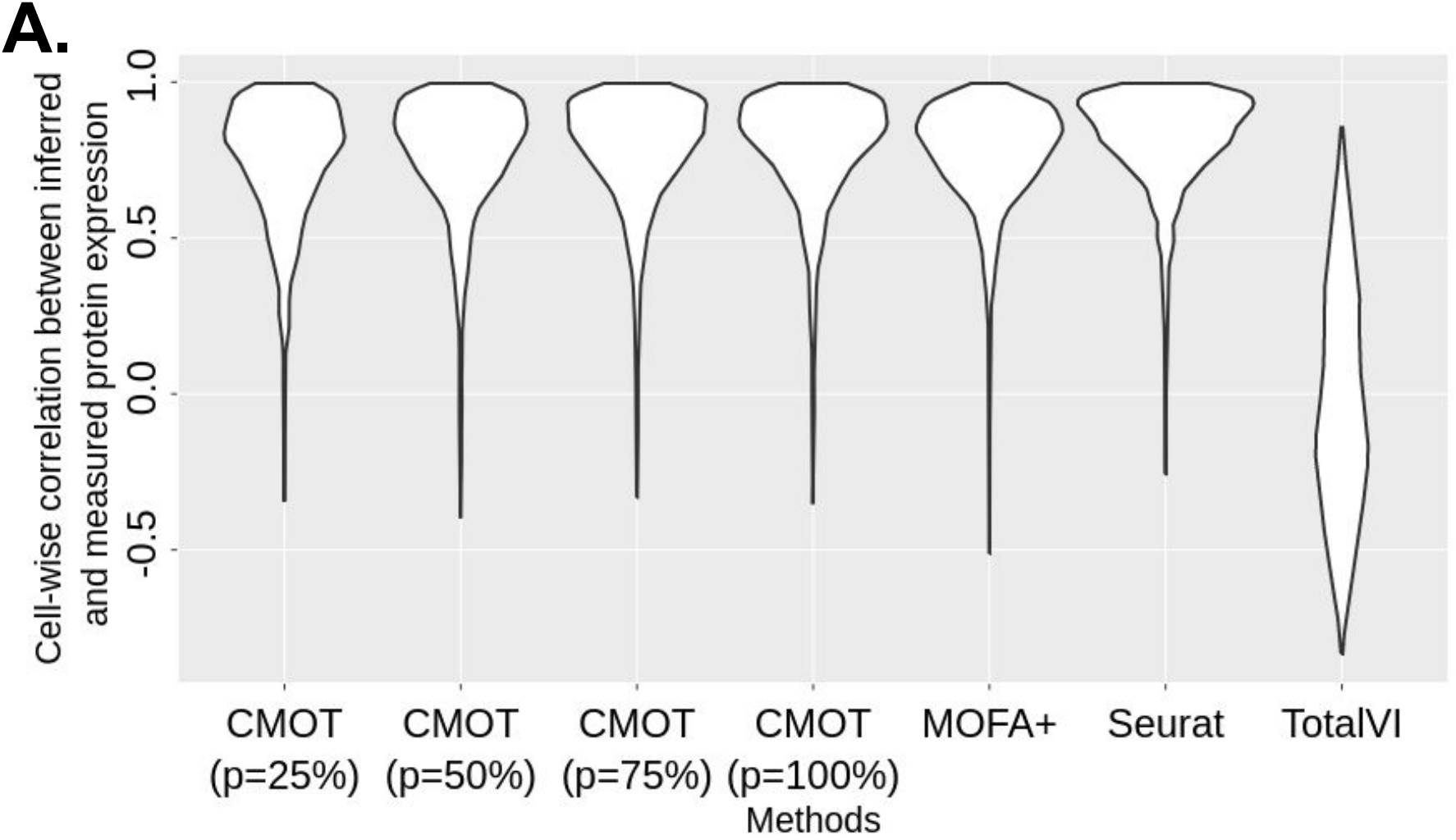 |
| --- |
| **Figure S7:** **Cross-modality inference between protein and gene expression in PBMC(10k) [5] (A)** Cell-wise Pearson correlation (y-axis) of inferred and measured protein expression by different methods (x-axis): CMOT (*p*=25%,50%,75%,100%), Seurat, MOFA+, TotalVI (**Tables S14-S16**) [5]. |

| 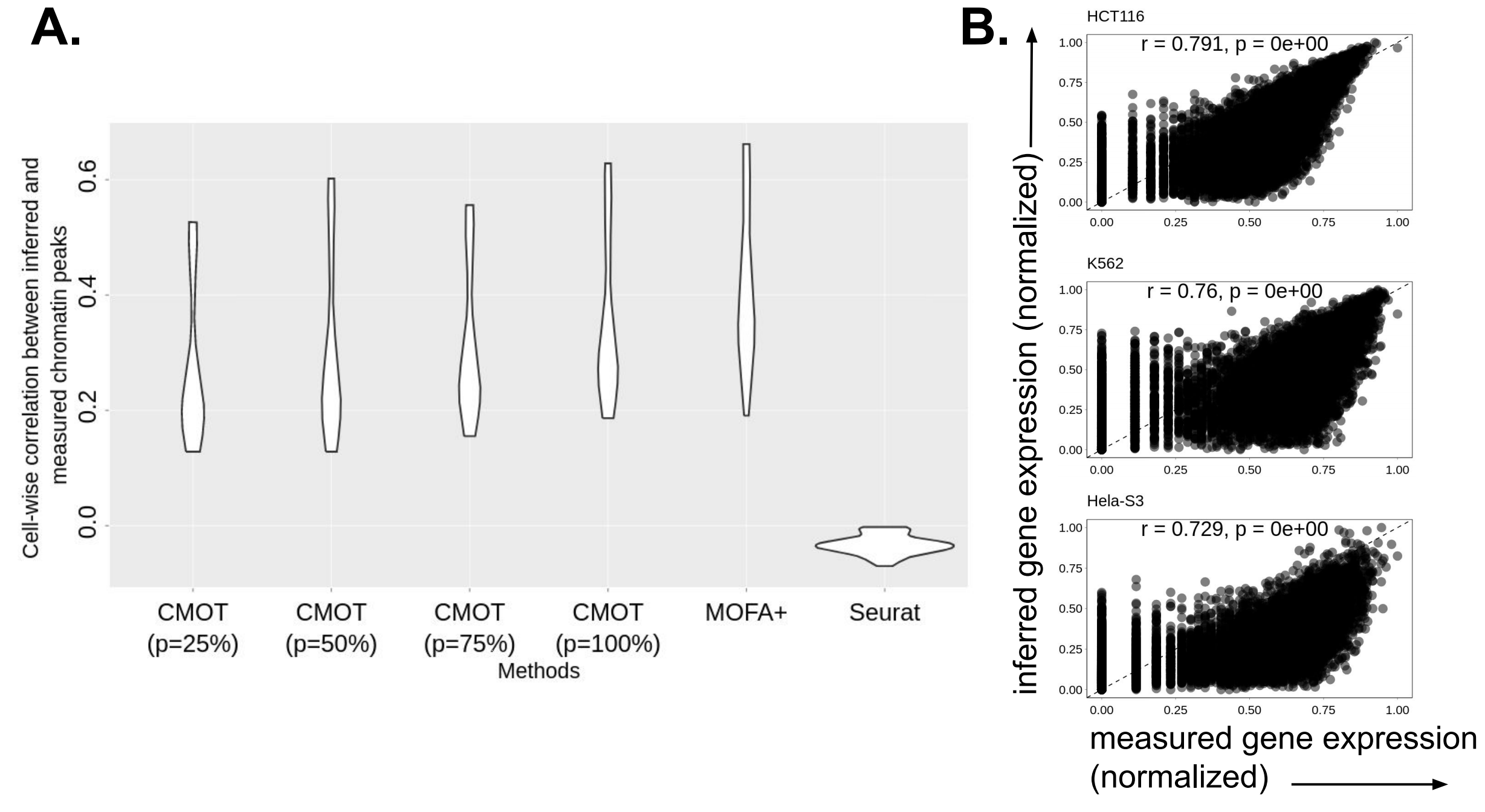 |
| --- |
| **Figure S8:** **Cross-modality inference between gene expression and chromatin accessibility in pan-cancer cells [3] (A)** Cell-wise Pearson correlation (y-axis) of inferred and measured chromatin accessibility by different methods (x-axis): CMOT (*p*=25%,50%,75%,100%), Seurat, MOFA+ (**Tables S27-S28**) [3]. **(B)** The measured (x-axis) versus inferred normalized expression (y-axis) of genes (dots) for three select cells. r is the Pearson correlation coefficient. p is the correlation p-value. |

| 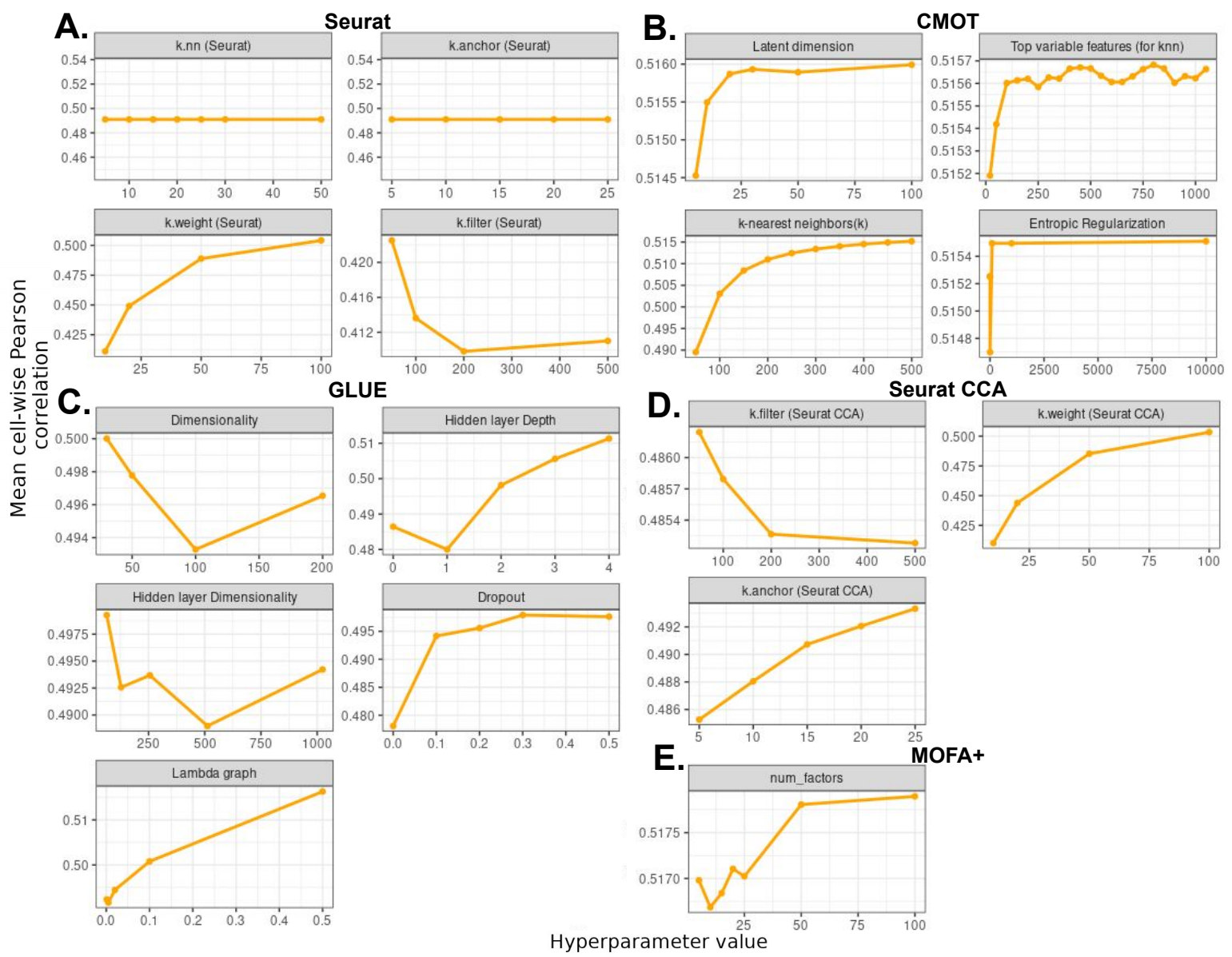 |
| --- |
| **Figure S9:** **Inference performance of all methods under different hyperparameter settings on DEX-treated A549 dataset [2].** **(A)** Seurat hyperparameters - k.nn: the number of multimodal neighbors to compute (default=20); k.anchor: How many neighbors (k) to use when finding anchors (default=5); k.weight: Number of neighbors to consider when weighting anchors (default=50); k.filter: How many neighbors (k) to use when filtering anchors (default=200); **(B)** CMOT hyperparameters – Latent dimension: latent dimension for alignment (step A); Top variable features: top high variables for k-nearest neighbors; k-nearest neighbors: number of neighbors for inference; Entropic regularization: entropic regularization parameter; **(C)** GLUE hyperparameters – Dimensionality: cell embedding dimensionality. Hidden layer depth: number of hidden layers in the data encoders and modality discriminator. Hidden layer dimensionality: dimensionality of hidden layers in the data encoders and modality discriminator. Dropout: dropout rate of hidden layers in data encoders and modality discriminator. Lambda graph: weight of the graph loss **(D)** Seurat CCA hyperparameters - k.anchor: How many neighbors (k) to use when finding anchors (default=5); k.weight: Number of neighbors to consider when weighting anchors (default=50) ; k.filter: How many neighbors (k) to use when filtering anchors (default=200); **(E)** MOFA+ hyperparameter - num_factors: latent factors. |

| 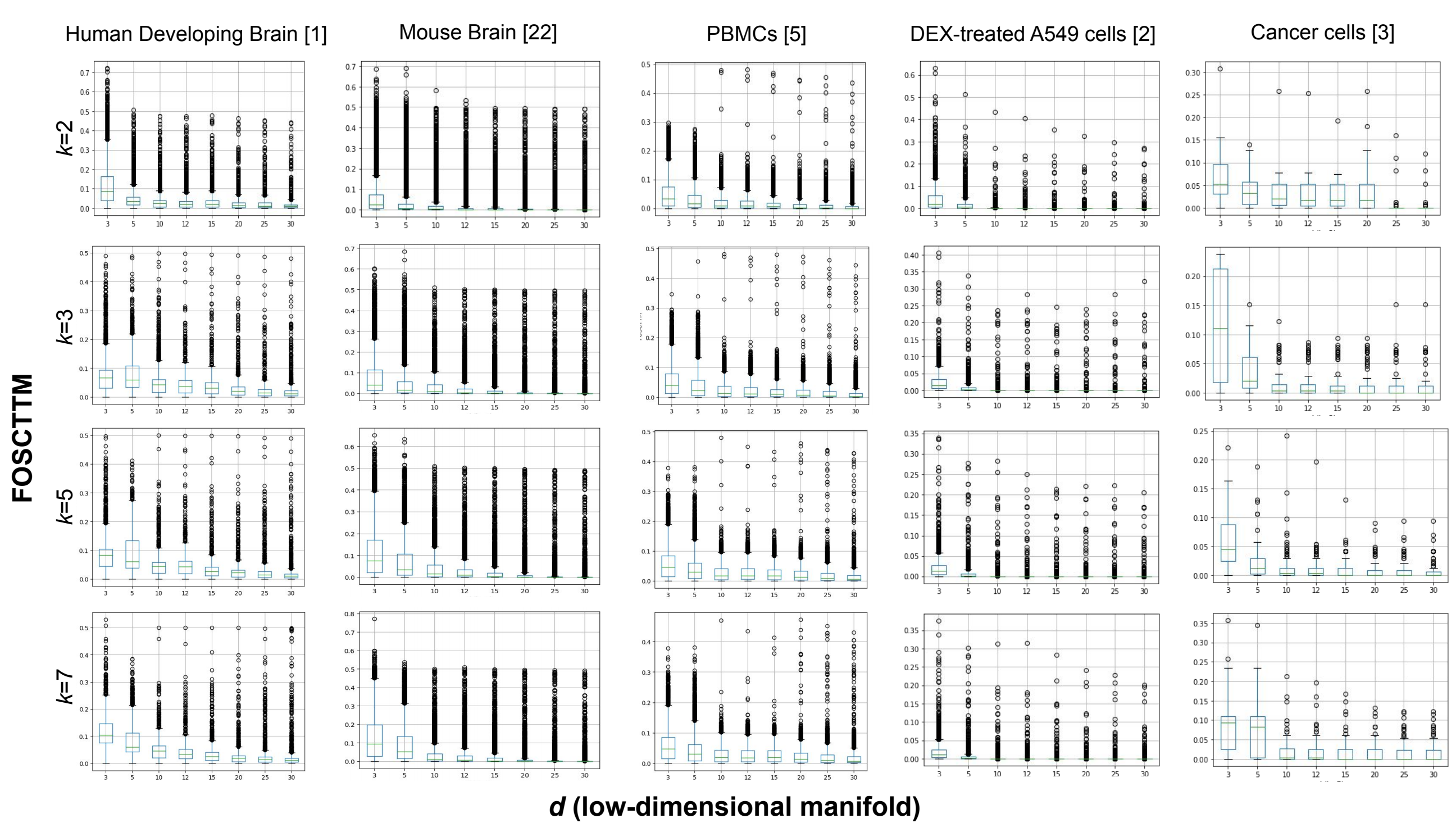 |
| --- |
| **Figure S10:** Boxplots show the pairwise cell Mean FOSCTTM Score (y-axis) after alignment on the latent dimension *d* (x-axis) for different choices of *k* nearest neighbors (row) in non-linear manifold learning (NMA) for different datasets (column). |

| 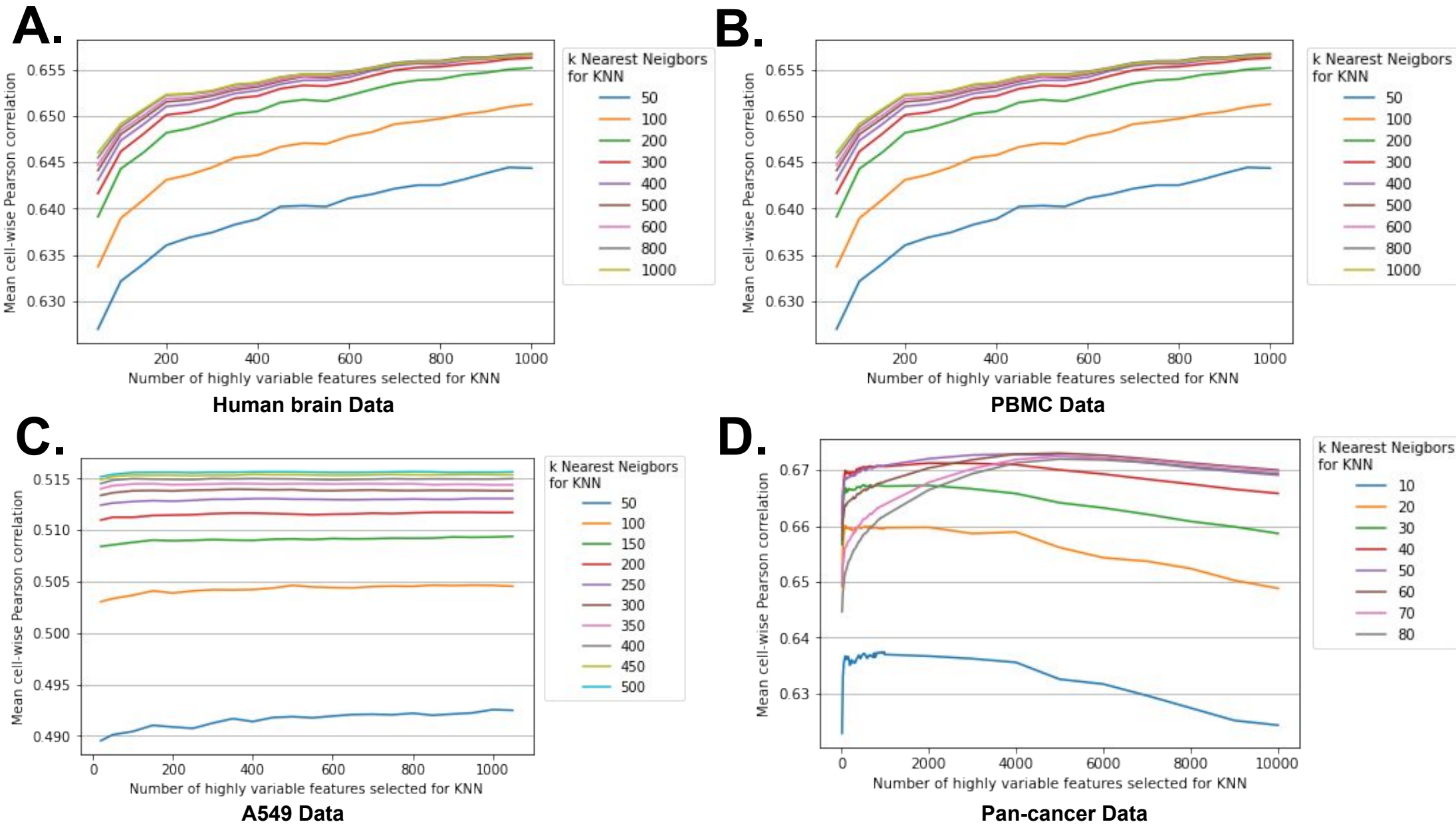 |
| --- |
| **Figure S11: Mean cell-wise Pearson correlation across top highly variable features for *k* nearest neighbors (A)** Mean cell-wise Pearson correlation (y-axis) of inferred and measured gene expression by CMOT across a different number of top highly variable genes (x-axis) in human brain data [1]. **(B)** Mean cell-wise Pearson correlation (y-axis) of inferred and measured gene expression by CMOT across a different number of top highly variable genes (x-axis) in PBMC [5]. **(C)** Mean cell-wise Pearson correlation (y-axis) of inferred and measured gene expression by CMOT across a different number of top highly variable genes (x-axis) in A549 data [2]. **(D)** Mean cell-wise Pearson correlation (y-axis) of inferred and measured gene expression by CMOT across a different number of top highly variable genes (x-axis) in pan-cancer data [3]. |

| 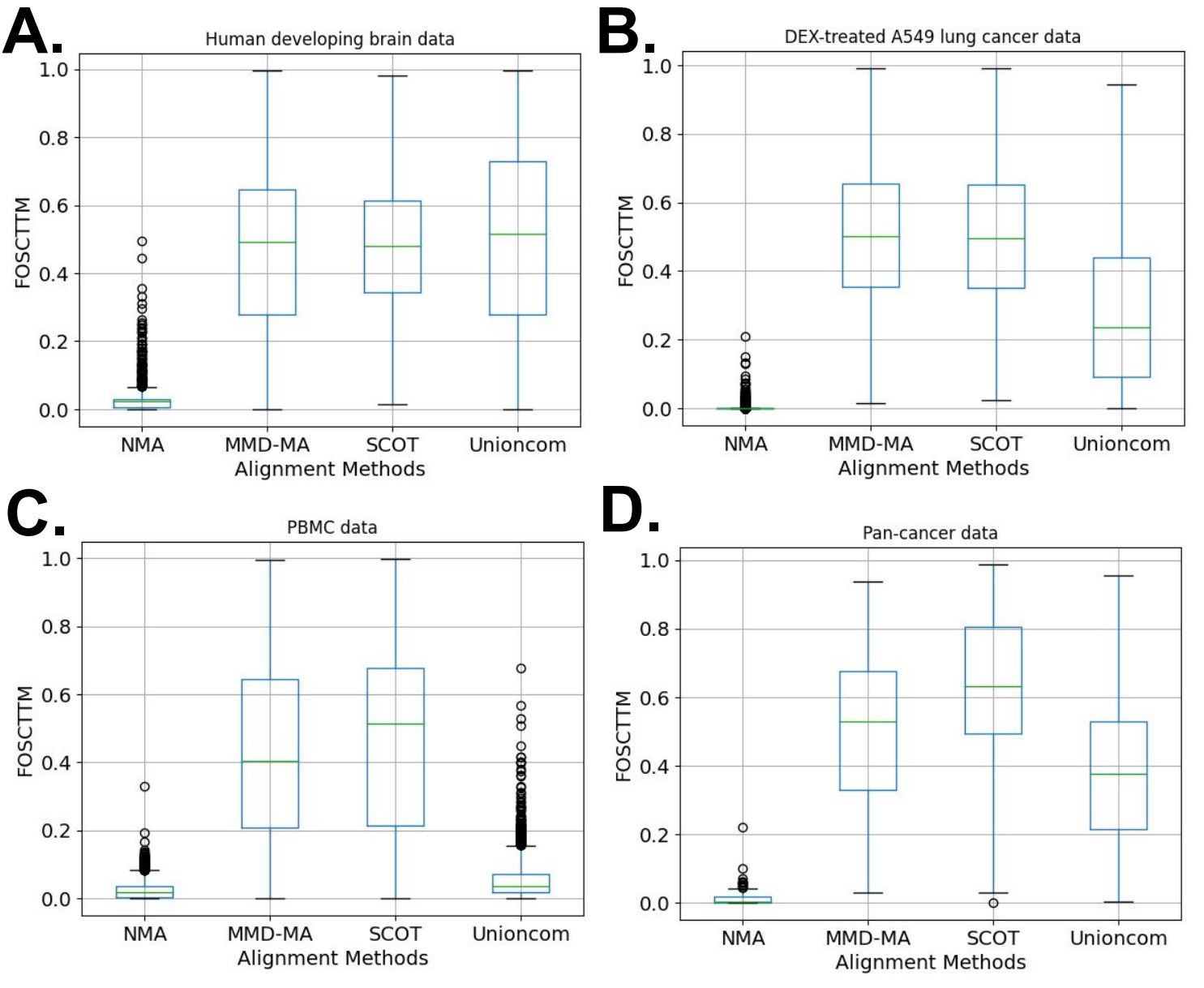 |
| --- |
| **Figure S12:** Boxplots show the FOSCTTM score for alignment methods: Nonlinear Manifold Alignment (NMA), Maximum mean discrepancy-based manifold alignment (MMD-MA), Single-cell alignment with optimal transport (SCOT) and Unioncom on the four datasets. The latent dimensions are the optimal dimensions reported for each dataset i.e. *d*=20 for human developing brain [1], *d*=15 PBMC [5], *d*=10 DEX-treated A549 lung cancer data [2], and, *d*=10 pan-cancer data [3]. |

| 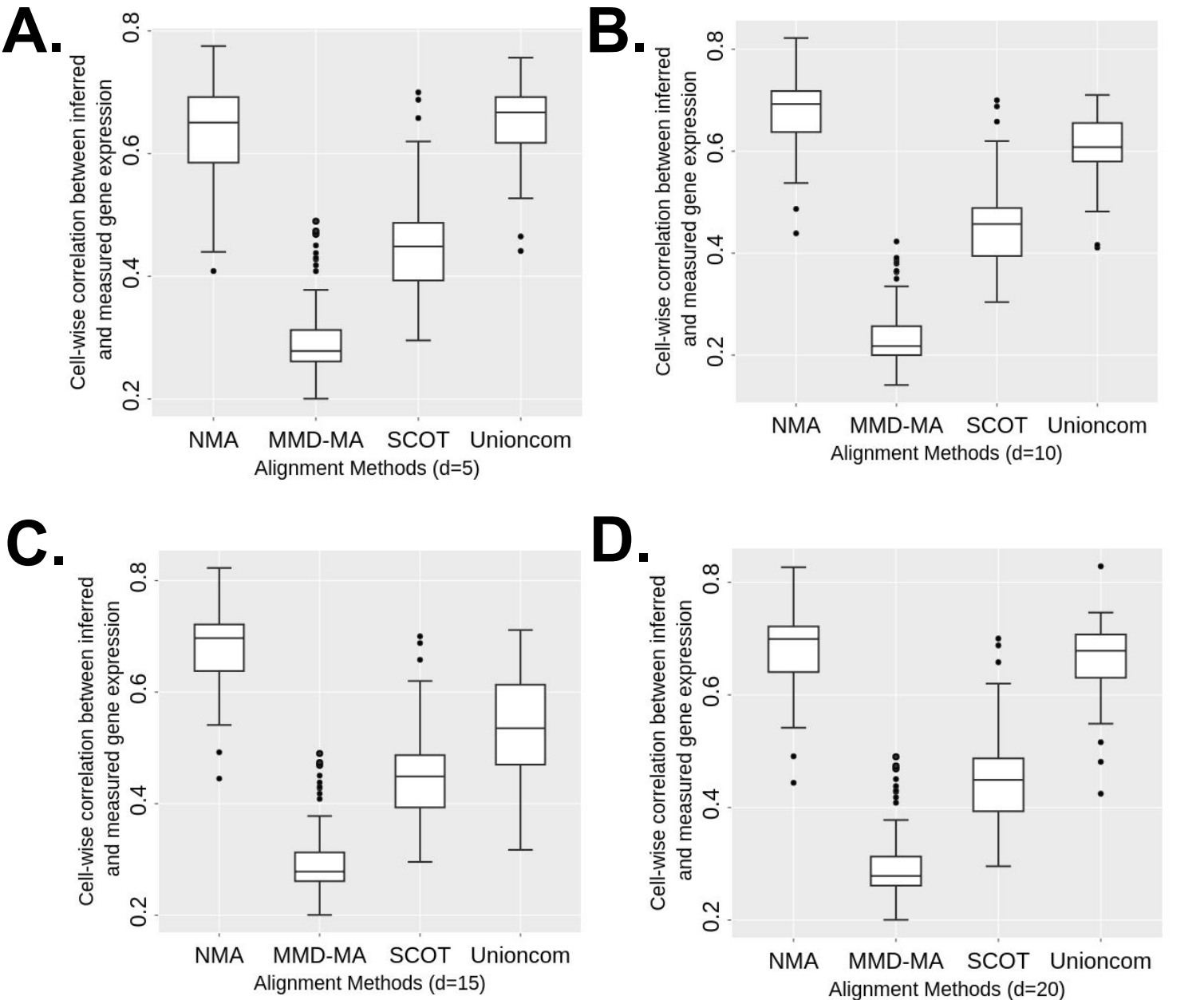 |
| --- |
| **Figure S13: Benchmarking alignment methods (NMA, MMD-MA, SCOT, Unioncom) to compare CMOT’s inference in pan-cancer data [3] (A)** Mean cell-wise Pearson correlation (y-axis) of inferred and measured gene expression by CMOT across alignment methods (x-axis) for *d*=5. **(B)** Mean cell-wise Pearson correlation (y-axis) of inferred and measured gene expression by CMOT across alignment methods (x-axis) for *d*=10. **(C)** Mean cell-wise Pearson correlation (y-axis) of inferred and measured gene expression by CMOT across alignment methods (x-axis) for *d*=15. **(D)** Mean cell-wise Pearson correlation (y-axis) of inferred and measured gene expression by CMOT across alignment methods (x-axis) for *d*=20. |

| 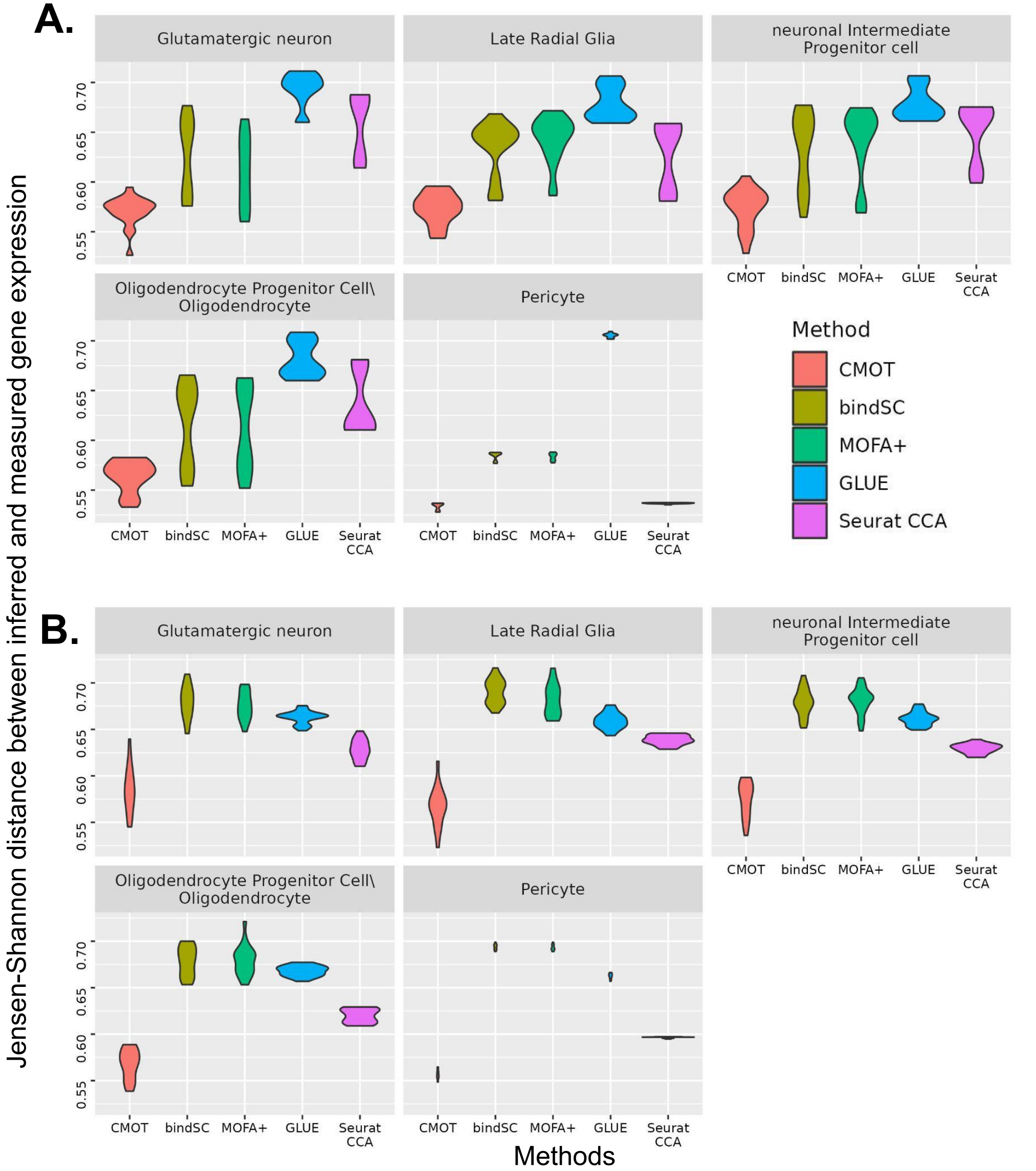 |
| --- |
| **­Figure S14: Gene expression inference from chromatin accessibility on scRNA-seq and scATAC-seq data from human developing brain data [1] (A)** Jensen-Shannon distance (y-axis) between measured and inferred gene expression by CMOT, bindSC, MOFA+, GLUE, and, Seurat CCA (x-axis) for 1000 highly variable genes on five major celltypes. **(B)** Jensen-Shannon distance (y-axis) between measured and inferred gene expression by CMOT, bindSC, MOFA+, GLUE, and, Seurat CCA (x-axis) for 2000 highly variable genes on five major celltypes. |

# Supplemental Methods

### Datasets preprocessing and feature selection

**Mouse brain:** The adult mouse brain dataset [22] was generated by SNARE-seq, containing jointly profiled gene expression and open chromatin regions for ~10k cells. However, we used the previously processed dataset by Cao et al. [21] (published with GLUE), including gene expression with 28,930 genes and open chromatin peaks with 241757 peaks for 9190 cells from the adult mouse brain. However, we reduced the size of the dataset by picking 2000 highly variable genes and peaks. The resulting data includes gene expression and open chromatin peaks of 9190 cells for 2000 genes and peaks. To compare CMOT with Polarbear we used their data with ~10k cells. However, we reduced the number of genes and peaks. We filtered out peaks and genes that occurred in less than 3 cells. For the binary scATAC data, we picked the top 1000 peaks that were expressed in the largest number of cells. For scRNA, we performed normalization and variance stabilization using SCTransform [41] and picked the top 1000 most variable genes. The resulting data includes gene expression and chromatin regions of 10,839 cells for 1000 genes and regions respectively.

**Peripheral Blood Mononuclear Cells:** The PBMC10k dataset [5] contains 6855 cells, containing genes and proteins from the same cells. For scRNAseq, we performed normalization and variance stabilization using SCTransform [41], and used the 2960 highly variable genes. For protein expression, we performed centered log-ratio (CLR) normalization using Seurat’s functions. The resulting dataset includes 6855 cells with 2960 genes and 14 proteins.

### Inferring protein expression from gene expression in Peripheral Blood Mononuclear Cells

We applied CMOT to infer protein expression from gene expression of peripheral blood mononuclear cells (PBMCs) using emerging CITE-seq data [5]. We randomly split the PBMC10k cells into 80% training for cross-validation and 20% testing set for evaluation. We trained CMOT with parameters: *K*=5, *d=* 15, λ=1e02, η = 1, *k*=100, and used the top 200 highly variable genes in the training data to find the *k* nearest neighbors. We induced cell labels by identifying two clusters using gene expression for the label regularization in optimal transport. As shown in Fig. S4, CMOT achieves a median correlation *r* of 0.91 for *p*=100% outperforming MOFA+ (median *r*=0.89, Wilcoxon p-values<1.22e-05) and TotalVI (median *r*=-0.08, Wilcoxon p-values=0) and performs comparably with Seurat (median *r*=0.92, Wilcoxon p-values<0.99). Also, for *p=*50%,75%, CMOT reports a higher correlation of 0.9 for both, outperforming both MOFA+ (Wilcoxon p-values<1.4e-03) and TotalVI (Wilcoxon p-values=0) (Tables S14-S16).

### Benchmark against state-of-art for cross-modality inference

We benchmarked CMOT against state-of-art methods: Seurat [7], Seurat CCA[8], MOFA+ [9], bindSC [20], GLUE [21], and, Polarbear [11]. We show benchmarking on 3 datasets: (1) mouse brain data [22] (GLUE’s application), (2) DEX-treated A549 data [2] (common application between CMOT and bindSC), and, (3) human developing brain [1] (our application),

For the mouse brain dataset [22], which is an application of GLUE [21], we used their preprocessed dataset to benchmark all methods. We split this dataset into 80% train and 20% test, and benchmarked all methods on default parameters. For CMOT, we used the following parameters: *K*=5, *d=* 20, λ=1e03, η=1e-2, *k*=400, and used the top 10 features of scATAC-seq training data to find the *k* nearest neighbors. We induced our cell labels by hierarchical clustering of the cells in the training set and identified two clusters to regularize the optimal transport in CMOT. As shown in Fig S2, CMOT outperforms state-of-art methods with a median correlation of 0.76 compared to Seurat (median correlation=0.7, Wilcoxon p-value<3.4e-40), MOFA+(median correlation=0.74, Wilcoxon p-value<1.15e-05), GLUE(median correlation=0.72, Wilcoxon p-value<5.6e-25), bindSC (median correlation=0.72, Wilcoxon p-value<8e-19), and, Seurat CCA (median correlation=0.75, Wilcoxon p-value<1.9e-04). Even for partial correspondences, CMOT reports a higher performance (median *r*=0.76 for *p*=75%, median *r*=0.76 for *p*=50%) continuing to outperform other methods: Seurat (Wilcoxon p-value<1.25e-39), GLUE (Wilcoxon p-value<1.63e-24), MOFA+ (Wilcoxon p-value<1.67e-05), bindSC (Wilcoxon p-value<2.07e-158), Seurat CCA (Wilcoxon p-value<2.8e-4). Even for correspondence as low as 25%, CMOT performs comparably with median *r*=0.71, still outperforming Seurat (Wilcoxon p-value<0.07) (see Additional File 1: Fig S2, Supplemental Methods, Supplemental Table S5-S8). We also compared CMOT with Polarbear on their mouse brain data for fair comparison using top 1000 features. We found that CMOT has significantly higher gene-wise correlations than Polarbear (515 genes versus 485 genes, Wilcoxon p-value<2.81e-02) and Polarbear co-assay (648 genes versus 352 genes, Wilcoxon p-value<1.2e-27), and, Seurat (1960 genes versus 101 genes, Wilcoxon p-value<2.97e-69) (Fig. S3, Tables S9-S10).

For the DEX-treated A549 dataset [2], which is a common application of CMOT and bindSC, we also split the dataset into 80% train and 20% test, similar to Fig 4. To run bindSC, we used the parameters suggested in their dataset tutorial [20]. We benchmarked other methods on tuned parameters on 80% of training data (Fig S9). After tuning, we used the following parameters for benchmarking:  A) Seurat: k.nn=20, k.anchor=5, k.weight=100, k.filter=50; B) CMOT: latent dimension=25, top variable features=20, k-nearest neighbors=500, entropic regularization=100; C) GLUE: Dimensionality=30, Hidden layer depth=4, Hidden layer dimensionality=64, Dropout=0.3, Lambda graph=0.5; D) Seurat CCA: k.filter=50, k.weight=100, k.anchor=25; E) MOFA+: num_factors=100. As shown in Additional File 1: supplemental Fig S7, CMOT outperforms state-of-art with a median correlation *r* of 0.52, compared to Seurat (median *r*=0.5, Wilcoxon p-value<21.27e-05), GLUE(median *r*=0.5, Wilcoxon p-value<4.7e-06), bindSC (median *r*=0.51, Wilcoxon p-value<0.016), Seurat CCA (median *r*=0.51, Wilcoxon p-value<0.016) and performs comparably to MOFA+(median *r*=0.52, Wilcoxon p-value<0.64). Even for partial correspondences, CMOT reports high and consistent performance (median *r*=0.52 for *p*=75%, median *r*=0.51 for *p*=50%) outperforming Seurat (Wilcoxon p-value<3.9e-05), GLUE (Wilcoxon p-value<1.6e-05), bindSC (Wilcoxon p-value<0.025), and, Seurat CCA (Wilcoxon p-value<0.029). (see Fig S7, Tables S17-S20).

To benchmark the human developing brain data [1], we split the dataset into 80% train and 20% as in Fig. 1. We trained all methods on default parameters and for CMOT, we used the same parameters as in Fig. 2. As shown Fig. S1, CMOT outperforms or performs comparably with state-of-arts with a median correlation of 0.67, compared to Seurat (median correlation=0.64, Wilcoxon p-value<1.23e-14), MOFA+(median correlation=0.41, Wilcoxon p-value=0), GLUE(median correlation=0.47, Wilcoxon p-value<1.4e-236), bindSC (median correlation=0.68, Wilcoxon p-value<0.97), and Seurat CCA (median correlation=0.68, Wilcoxon p-value<0.98). Also, for *p*<100%, CMOT continues to report consistent performance. For example, CMOT has significantly higher performances (median r=0.65 for *p*=75%, and r=0.63 for *p*=50%) than MOFA+ (Wilcoxon p-value<2.8e-294), GLUE (Wilcoxon p-value<1.3e-216), and Seurat (Wilcoxon p-value<3.43e-10). Also, with low correspondence such as *p*=25%, CMOT’s performance (*r*=0.61) is still significantly higher than MOFA+ (Wilcoxon p-value<1.65e-157) and GLUE (Wilcoxon p-value<2.4e-86) (see Fig S1, Tables S1-S4).

### Benchmarking on scRNA-seq and scATAC-seq datasets

We next benchmarked CMOT’s performance on single profiled scRNA-seq and scATAC-seq datasets from the developing human brain (week 21) [1]. We picked the top five major cell types common to both datasets: Glutamatergic neuron, Late Radial Glia, neuronal Intermediate Progenitor cells, Oligodendrocyte Progenitor Cell\Oligodendrocyte, and Pericyte. This resulted in 7420 cells from scATAC-seq and 6542 cells from scRNA-seq. We preprocessed each dataset similar to previous datasets, i.e. for scRNA-seq, we performed normalization and variance stabilization using SCTransform [41], and for scATAC-seq, we normalized the peaks using term frequency-inverse document frequency (TF-IDF) transformation using RunTFIDF [40]. Also, we conducted two evaluations by selecting 1000 and 2000 highly variable features. Finally, we split each profile into 80% training and 20% testing sets and evaluated CMOT against bindSC, MOFA+, GLUE, and Seurat CCA (Fig. S14). We ran all methods on default or recommended parameter settings. For CMOT, we used the same parameters as those used for the first application on developing humain brain. It is important that the inferred expressions must preserve the cell type distributions, hence we computed the Jensen-Shannon distance between cells from each cell type. We found that CMOT outperformed the state-of-art methods.

**Jensen-Shannon distance** **(JS distance):** The Jensen-Shannon distance measures the similarity between two probability distributions, and is the square root of Jensen-Shannon divergence (JSD). JSD is the average KL divergence between two distributions and their average (also the symmetric version of the KL divergence). KL divergence measures the difference between two probability distributions and increases proportionally. The smaller the JS distance, the closer are two probability distributions.

### Benchmarking alignment methods

We benchmarked CMOT’s performance across different alignment methods including Nonlinear Manifold Alignment (NMA) [10], Maximum mean discrepancy-based manifold alignment (MMD-MA) [27], Single-cell alignment with optimal transport (SCOT) [15], and Unioncom [28] (Fig S13, Fig S12 D) on the pan-cancer data [3]. We experimented with a different number of latent dimensions d=5,10,15,20 while using the default parameters for all methods. We evaluated CMOT’s performance on the pan-cancer data [3] across all dimensions and found that using NMA gives the best inference results (Fig S13). Additionally, we also evaluate the performance of alignment methods using the FOSCTTM score across all datasets (Fig. S12) for latent dimensions reported in the Results. We see that NMA significantly outperforms all other alignments.

### Outlier cell detection in target modality

We tested our outlier detection mechanism in the DEX-treated A549 dataset [2], where we randomly replaced 25% of cells in the test dataset with noisy cell samples. We generated the noisy cell samples by generating random real numbers within the interval of minimum and maximum values of the normalized chromatin expression. The IF mechanism can successfully identify the noisy cells, with an AUC=0.99.
