# Supplementary material for: CMOT: Cross-Modality Optimal Transport for multimodal inference
Source: Genome Biol. 2023 Jul 11;24:163. doi: 10.1186/s13059-023-02989-8 (PMC10334579; doi:10.1186/s13059-023-02989-8)
Supplement: Supplementary file 4 — Additional file 4. Review history [file 13059_2023_2989_MOESM4_ESM.docx]

**1^st^ round**

**Reviewer 1**

Single-cell multi-omics data presents comprehensive measurments of cellular states and thus can help derive a deep understanding of the cellular and molecular mechanisms. Despite the technical advances, simultaneous profiling of different modalities is not always avaiable in many applications. To resolve this challenge, Alatkar et al. developed CMOT to infer the missing modalities using cross-modality optimal transport (OT). The method first employs OT to map the target cells to the source cells (with both modalities) in the same modality. Then, based on mapping the same modality, CMOT uses the kNN to infer the missing modality of target genes using the known modality of the nearest mapped source cells.

Overall, I agree with the authors that: 1) the method has great potential in application scenarios. Namely, it could indeed help 'generate' and 'infer' missing modalities, which could substantially benefit the downstream analysis. 2) The proposed idea is feasible and is able to "infer" the missing modality. The approach is very straightforward (e.g., OT mapping and kNN). Particularly, the kNN strategy is non-parametric, which does not assume any data distributions. This, in many cases, could present a very robust imputation.

However, there are many potential caveats associated with the proposed method that the authors did not discuss in the manuscript.

Major comments:

(1) The method really relies upon a source dataset with simultaneous profiling of different modalities. In other words, the method needs to know the cell correspondence between modalities in the source data. Nevertheless, such information is not always avaiable. Most currently available single-cell multi-omics measurements are still non-simultaneous but profiled from different cells. To address this issue, the authors did provide an optional step A to infer or build the cell correspondence in the source data using Nonlinear manifold alignment. With that said, the accuracy of "Step A" is very critical for the overall performance of the entire method. The method performance is unclear on the data with unknown cell correspondence between modalities in the source data. The authors applied the method to a few datasets, all of which are simultaneous single-cell multi-omics (i.e., 10x genomics multiome, SNARE-seq, CITE-seq, sci-CAR).

(2)KNN mapping

Although the non-parametric kNN might be robust for imputing the missing modality as it doesn't assume any data distribution, it also suffers from a significant drawback, which may significantly affect the application of the method. kNN method does NOT reject imputation even in the cases of no good mapping being obtained. In other words, it always finds the nearest neighbors for a target cell. However, even the target cell is very different from all cells in the source data. The method will still find the "nearest" and use it to infer the missing modality, presenting a false imputation. The proposed method needs mechanisms to prevent this from happening. This problem will become severe if the source and target datasets differ significantly and thus significantly limit the method's application.

(3)The method has NOT benchmarked with a few recent methods in the same category. For example, scGLUE (https://www.nature.com/articles/s41587-022-01284-4). The authors could benchmark their method with a few SOTA methods published in recent years.

Minor comments:

(1) line 119, specify the summarization (over what?)

(2) Line 174, Is the Euclidean distance on the original feature space? If so, it might be problematic. Euclidean distance in high-dimensional space is misleading (curse of dimensionality). If on reduced space, please clarify.

**Reviewer 2**

Alatkar & Wang present here approach for matching single cells for multimodal intference, CMOT, including using optimal transport to deduce the missing modalities.

The method appears to be relatively novel, but would benefit from substantially more benchmarking against other state-of-the-art methods beyond the limited ones in the current manuscript. There are also some potential caveats with the way the training and inference was performed for some of the datasets, as elaborated below.

Major comments:

1. It is noteworthy that CMOT does not outperform other methods in scenarios where 100% of the cells are multiomic, but appears to have an improved performance when <100% of the cells are multiomic. However, based upon the benchmarking performed in this study, it is difficult for readers to properly assess the robustness of this cross-modality inference, such as Seurat CCA, BindSC, scGLUE, or even SpaOTsc (which also utilizes OT).

2. A key limitation is the relatively simplistic benchmarking metrics used here: a correlation of gene expression. There are other metrics that should be utilized to evaluate the performance of multimodal cell matching. These include methods used in portions of the paper, such as FOSCTTM, silhouette score, or a confusion matrix for assessing accuracy.

3. The training/validation data split varies significantly across the datasets used across the figures. These splits should be consistent/justified and also avoid overfitting the data. For instance

a. Fig 2 is 80% training/20% validation

b. Fig 3 is 6885 cells training/3994 validation

c. Fig 4 is 80% training/20% validation

d. Fig 5 is 60% training 40% validation

4. How the custom parameter selection was made is unclear for CMOT (which varies across the data), whereas the benchmarked other methods used was default. How much of the difference in performance of CMOT versus others was a result of parameter tuning?

5. The number of features used for the inference also varied greatly between datasets, eg

a. Fig 2: top 1000

b. Fig 3: top 200

c. Fig 4: top 20

d. Fig 5: top 150

e. How were these top parameters selected, and how much variation in performance across the methods do we see as a result of these?

6. How much of the separation seen in cancer cell lines in Fig 5 can be done using only chromatin accessibility, compared to the inferred genes? I.E. is CMOT necessary for this separation

7. The authors mentioned other methods eg SCOT/MMD-MA can be used as alignment approaches. How much does the selection of alignment approaches affect CMOT inference performance?

Minor Comments:

1. Line 99, missing words after "CMOT first aligns the [sic]."

2. Is there a rational for using Pearson and not a ranked based correlation approach for calculating cell-wise correlations in this study?

3. Median correlations are shown instead of mean. How different are the results in this case?

4. The parameter mu for line 119 was set to 0.5. Is this a value that has been optimized or chosen for a particular reason?

5. Line 274: Is it a typo for "top 8 highly predictive cells" or genes?

6. Why was Seurat & MOFA not used for the inference in the Mouse Brain data for consistency, where CMOT was compared with Polarbear?

7. Violin plots are much preferred over boxplots eg in Fig 3A due to their ability to display distribution better

8. The number of cells used for Fig 5 was not specified, since this number was detailed for the other datasets, it will be good for consistency

9. I noticed the correlation by all methods were consistently lower compared to other figures. Does this mean this data is of lower quality or perhaps is a more challenging dataset to predict? The authors should comment on this for readers.

**Authors’ response**

Reviwer #1

Single-cell multi-omics data presents comprehensive measurements of cellular states and thus can help derive a deep understanding of the cellular and molecular mechanisms. Despite the technical advances, simultaneous profiling of different modalities is not always available in many applications. To resolve this challenge, Alatkar et al. developed CMOT to infer the missing modalities using cross-modality optimal transport (OT). The method first employs OT to map the target cells to the source cells (with both modalities) in the same modality. Then, based on mapping the same modality, CMOT uses the kNN to infer the missing modality of target genes using the known modality of the nearest mapped source cells.

Overall, I agree with the authors that: 1) the method has great potential in application scenarios. Namely, it could indeed help 'generate' and 'infer' missing modalities, which could substantially benefit the downstream analysis. 2) The proposed idea is feasible and is able to "infer" the missing modality. The approach is very straightforward (e.g., OT mapping and kNN). Particularly, the kNN strategy is non-parametric, which does not assume any data distributions. This, in many cases, could present a very robust imputation.

However, there are many potential caveats associated with the proposed method that the authors did not discuss in the manuscript.

Thank the reviewer. We appreciate the valuable feedback and have addressed the comments as follows.

The method really relies upon a source dataset with simultaneous profiling of different modalities. In other words, the method needs to know the cell correspondence between modalities in the source data. Nevertheless, such information is not always available. Most currently available single-cell multi-omics measurements are still non-simultaneous but profiled from different cells. To address this issue, the authors did provide an optional step A to infer or build the cell correspondence in the source data using Nonlinear manifold alignment. With that said, the accuracy of "Step A" is very critical for the overall performance of the entire method. The method performance is unclear on the data with unknown cell correspondence between modalities in the source data. The authors applied the method to a few datasets, all of which are simultaneous single-cell multi-omics (i.e., 10x genomics multiome, SNARE-seq, CITE-seq, sci-CAR).

Thank the reviewer for pointing out this. We tested missing corresponding scenarios by intentionally dropping correspondence information between randomly chosen cells of multimodalities. Here, we tested for different dropout percentages p i.e. 25%, 50%, 75%, and 100%. We see that even when correspondence < 100%, CMOT performs reasonably well compared to other state-of-art methods. For example, CMOT has significantly higher performances (median r =0.68 for p=75%, and r=0.65 for p=50%) than MOFA+ (median r=0.41, Wilcoxon p-value<2.8e-294), Seurat (median r=0.64, Wilcoxon p-value<3.43e-10), GLUE (median r=0.47, Wilcoxon p-value<1.4e-236), and is still closer in performance to bindSC (median r=0.68, Wilcoxon p-value=0.99) and Seurat CCA (median r=0.68, Wilcoxon p-value=0.99). Also, with low correspondence such as p=25%, CMOT’s performance (median r=0.61) is still significantly higher than MOFA+ (Wilcoxon p-value < 1.65e-157) and GLUE (Wilcoxon p-value < 1.65e-157). Below we show the results from one of our datasets (Fig. 6A).

*Methods (Page 13 Line 379)*

*Partial correspondence in multi-omics data: Joint profiling of single cells is challenging and therefore, it may not always be feasible to get completely corresponding cells across profiled modalities. In such scenarios, there could be partial to no correspondence across cells of multimodalities. For example, a 50% cell-to-cell correspondence between modalities means that only 50% of the cells have been jointly profiled between the modalities. As a result, training on partially corresponding multimodalities for cross-modality inference can lead to misleading or wrong inferences. Therefore, to address this problem, CMOT first aligns such partially corresponding datasets and then performs inference. In this paper, we have used datasets that have a 100% correspondence originally, so that we can validate the inference performance. However, we simulate different levels of cell-to-cell correspondence by setting the p value in non-linear manifold alignment (Methods Step A). In particular, we randomly chose p percent cells for whom correspondence information is assumed available, while the remaining cells are treated as non-corresponding. We report CMOT's performance when trained on p=25%, 50%, 75%, 100% cell-to-cell correspondence levels, and show that CMOT's cross-modality inference performance can beat state-of-the-art methods that require 100% cell-to-cell correspondence for training.*

*Supplemental Fig S1 Gene expression inference from chromatin accessibility in human brain data [1] (A) Cell-wise Pearson correlation (y-axis) of inferred and measured chromatin accessibility by different methods (x-axis): CMOT (p=25%,50%,75%,100%), Seurat, MOFA+, GLUE, bindSC, Seurat CCA (Tables S1-S4) [1].*

Although the non-parametric kNN might be robust for imputing the missing modality as it doesn't assume any data distribution, it also suffers from a significant drawback, which may significantly affect the application of the method. kNN method does NOT reject imputation even in the cases of no good mapping being obtained. In other words, it always finds the nearest neighbors for a target cell. However, even the target cell is very different from all cells in the source data. The method will still find the "nearest" and use it to infer the missing modality, presenting a false imputation. The proposed method needs mechanisms to prevent this from happening. This problem will become severe if the source and target datasets differ significantly and thus significantly limit the method's application.

We thank the author for this insightful comment. We address this point by adding an additional mechanism after the optimal transport step (step B) which originally finds an optimal transport plan between cells of source (Y) and target (Ŷ) modalities. Then, we remove any poorly mapped cells from the target modality. This helps ensure that CMOT does not infer false expression profiles for cells in the target that may not share expression profiles with those in the available source modality. To identify poorly mapped target cells (Ŷ), we first multiply the optimal probabilistic mapping (ⲡ*∊RsY×sŶ) with the cost of transportation (C∊RsY×sŶ) calculated by optimal transport. The optimal mapping (ⲡ*(y,ŷ)) gives the probability of transportation between y and ŷ, whereas the C(y,ŷ) gives the cost of mapping between y,ŷ. Then we look for outlier target cells within the resulting product using the Isolation Forest [38] algorithm. If any outliers, we simply remove those cells from the target modality. We tested this mechanism on one of our applications (DEX-treated A549 data) by randomly replacing cells with noisy cell samples, and the mechanism could successfully identify and remove such cells (see Supplemental Methods). Below we show that the mechanism can identify such outlier cells easily. Users can use this mechanism if they think their source multimodalities differ significantly from the target single modality.

[38] F. T. Liu, K. M. Ting, and Z.-H. Zhou, “Isolation Forest,” in 2008 Eighth IEEE International Conference on Data Mining, Pisa, Italy, Dec. 2008, pp. 413–422. doi: 10.1109/ICDM.2008.17.

*Methods (Page 12 Line 351)*

*Identify outlier cells in target modality: Some cells from the target modality (Ŷ) may have a different distribution (e.g. belonging to a cell type absent in source modality). For such cells, the inference is difficult and may even lead to false predictions. To avoid this, we add an additional mechanism to identify and remove such cells. We use the optimal coupling ⲡ* and the cost matrix C calculated in step B, and compute an element-wise dot product P=ⲡ*T∘CT, where P∊RsŶ×sY. Then, we find any outliers within P using the Isolation Forest (IF) algorithm [38]. The Isolation Forest (IF) algorithm isolates samples by randomly selecting a feature and then randomly selecting a split value between the minimum and maximum values of the selected features. Since this algorithm is prone to the curse of dimensionality, we first apply principal component analysis (PCA) to P and use components that explain at least 95% variance. We then apply the IF to the lower dimensional P. We tested this mechanism by randomly replacing cells with noise in the DEX-treated A549 dataset (see Supplemental Methods)*

*Supplemental Methods*

*Outlier cell detection in target modality*

*We tested our outlier detection mechanism in the DEX-treated A549 dataset [2], where we randomly replaced 25% of cells in the test dataset with noisy cell samples. We generated the noisy cell samples by generating random real numbers within the interval of minimum and maximum values of the normalized chromatin expression. The IF mechanism can successfully identify the noisy cells, with an AUC=0.99.*

The method has NOT benchmarked with a few recent methods in the same category. For example, scGLUE (https://www.nature.com/articles/s41587-022-01284-4). The authors could benchmark their method with a few SOTA methods published in recent years.

We thank the reviewer for pointing this out. As suggested, we benchmarked CMOT with recent state-of-art methods including GLUE, bindSC, and, Seurat CCA. We tried the SNARE-seq data from scGLUE (see Fig S2, Supplemental Methods, Supplemental Table 5-8). As shown on the supple. Fig S2, CMOT outperforms state-of-art methods with a median correlation of 0.76 compared to Seurat (median correlation=0.7, Wilcoxon p-value<3.4e-40), MOFA+(median correlation=0.74, Wilcoxon p-value<1.15e-05), GLUE(median correlation=0.72, Wilcoxon p-value<5.6e-25), bindSC (median correlation=0.72, Wilcoxon p-value<8e-19), and, Seurat CCA (median correlation=0.75, Wilcoxon p-value<1.9e-04).

line 119, specify the summarization (over what?)

Thank the reviewer for pointing this out. We have fixed the formula by updating it to summarization over the cells, i,j.

*Methods (Page 11 Line 295)*

Line 174, Is the Euclidean distance on the original feature space? If so, it might be problematic. Euclidean distance in high-dimensional space is misleading (curse of dimensionality). If on reduced space, please clarify.

We thank the reviewer for the comment. We do calculate the euclidean distance in the original space, however, we do not choose all features to do so. We choose only top n highly variable features to find the k nearest neighbors (knn). We propose to use n as a hyperparameter to select the n highly variable subset of features that can be used for the knn step. Below we show the robustness of CMOT’s performance for the different number of features selected. We also clarified this in the revision. In the plots below, we show CMOT’s inference performance (y-axis) as the number of features selected varies (x-axis) for different values of k (nearest neighbors). We select the optimal number of features n and k based on performance saturation i.e. the line plot stops increasing.

*Methods (Page 15 Line 456)*

*Parameter selection*

*In Step A, we found the optimal alignment by testing different values of d common manifold dimensions and K nearest neighbors for building the similarity within each modality (Fig. S10). In Step B and Step C, we performed cross-validation to select the regularization coefficients 𝜆 and 𝜂 for optimal transport, and, top highly variable features and k-nearest neighbors for modality inference. In particular, we chose the optimal number of features and k-nearest neighbors based on CMOT’s performance saturation (Fig. S11). Also, for all datasets, applied in this paper, we held out a 20% testing set to report CMOT’s performance. For datasets with no prior knowledge (e.g., cell types), we induced cell labels by cell clusters through hierarchical clustering of the training set, when training the final model (see Supplementary Methods). We split the training data into training and validation sets to select parameters through 5-fold cross-validation (see Supplementary Methods).*

*Supplemental Fig S11 Mean cell-wise Pearson correlation across top highly variable features for k nearest neighbors Mean cell-wise Pearson correlation (y-axis) of inferred and measured gene expression by CMOT across a different number of top highly variable genes (x-axis) in human brain data [1]. (B) Mean cell-wise Pearson correlation (y-axis) of inferred and measured gene expression by CMOT across a different number of top highly variable genes (x-axis) in PBMC [5]. (C) Mean cell-wise Pearson correlation (y-axis) of inferred and measured gene expression by CMOT across a different number of top highly variable genes (x-axis) in A549 data [2]. (D) Mean cell-wise Pearson correlation (y-axis) of inferred and measured gene expression by CMOT across a different number of top highly variable genes (x-axis) in pan-cancer data [3].*

Reviewer #2

It is noteworthy that CMOT does not outperform other methods in scenarios where 100% of the cells are multiomic, but appears to have an improved performance when <100% of the cells are multiomic. However, based upon the benchmarking performed in this study, it is difficult for readers to properly assess the robustness of this cross-modality inference, such as Seurat CCA, BindSC, scGLUE, or even SpaOTsc (which also utilizes OT).

We thank the reviewer for pointing this out. As suggested, we benchmarked CMOT with recent state-of-art methods including GLUE, bindSC, and, Seurat CCA on three datasets.

First, we tried the mouse brain data from GLUE (see Fig S2, Supplemental Methods, Supplemental Table S5-S8). As shown in supplemental Fig S2, CMOT outperforms state-of-art methods with a median correlation of 0.76 compared to Seurat (median correlation=0.7, Wilcoxon p-value<3.4e-40), MOFA+(median correlation=0.74, Wilcoxon p-value<1.15e-05), GLUE(median correlation=0.72, Wilcoxon p-value<5.6e-25), bindSC (median correlation=0.72, Wilcoxon p-value<8e-19), and, Seurat CCA (median correlation=0.75, Wilcoxon p-value<1.9e-04). Even for partial correspondences, CMOT reports a higher performance (median r=0.76 for p=75%, median r=0.76 for p=50%) continuing to outperform other methods: Seurat (Wilcoxon p-value<1.25e-39), GLUE (Wilcoxon p-value<1.63e-24), MOFA+ (Wilcoxon p-value<1.67e-05), bindSC (Wilcoxon p-value<2.07e-158), Seurat CCA (Wilcoxon p-value<2.8e-4). Even for correspondence as low as 25%, CMOT performs comparably with median r=0.71, still outperforming Seurat (Wilcoxon p-value<0.07). Next, we tried the sci-CAR dataset containing A549 cells, from bindSC which was also one of our applications (see Fig S7, Supplemental Methods, Supplemental Table S17-S20). As shown in supplemental Fig S7, CMOT outperforms state-of-art with a median correlation r of 0.52, compared to Seurat (median r=0.5, Wilcoxon p-value<21.27e-05), GLUE(median r=0.5, Wilcoxon p-value<4.7e-06), bindSC (median r=0.51, Wilcoxon p-value<0.016), Seurat CCA (median r=0.51, Wilcoxon p-value<0.016) and performs comparably to MOFA+(median r=0.52, Wilcoxon p-value<0.64). Even for partial correspondences, CMOT reports high and consistent performance (median r=0.52 for p=75%, median r=0.51 for p=50%) outperforming Seurat (Wilcoxon p-value<3.9e-05), GLUE (Wilcoxon p-value<1.6e-05), bindSC (Wilcoxon p-value<0.025), and, Seurat CCA (Wilcoxon p-value<0.029).

Finally, we tried the 10XMultiome data, which was on one of our applications (see Fig S1, Supplemental Methods, Supplemental Table S1-S4). As shown in supplemental Fig S1, CMOT outperforms state-of-art with a median correlation r of 0.67, compared to Seurat (median correlation=0.64, Wilcoxon p-value<1.23e-14), MOFA+(median r=0.41, Wilcoxon p-value=0), GLUE(median r=0.47, Wilcoxon p-value<1.4e-236), bindSC (median r=0.68, Wilcoxon p-value<0.97), and Seurat CCA (median r=0.68, Wilcoxon p-value<0.98). Also, for p<100%, CMOT continues to report consistent performance. For example, CMOT has significantly higher performances (median r=0.65 for p=75%, and r=0.63 for p=50%) than MOFA+ (Wilcoxon p-value<2.8e-294), GLUE (Wilcoxon p-value<1.3e-216), and Seurat (Wilcoxon p-value<3.43e-10). Also, with low correspondence such as p=25%, CMOT’s performance (r=0.61) is still significantly higher than MOFA+ (Wilcoxon p-value<1.65e-157) and GLUE (Wilcoxon p-value<2.4e-86).

*Supplemental Information*

*Supplemental Fig S2 Gene expression inference from chromatin accessibility in mouse brain data [22] (A) Cell-wise Pearson correlation (y-axis) of inferred and measured gene expression by different methods (x-axis): CMOT(p=25%,50%,75%,100%), Seurat, MOFA+, GLUE, bindSC, and, Seurat CCA (Table S5-S8)*

*Supplemental Fig S7 Gene expression inference from chromatin accessibility in DEX-treated A549 [2] (A) Cell-wise Pearson correlation (y-axis) of inferred and measured chromatin accessibility by different methods (x-axis): CMOT (p=25%,50%,75%,100%), MOFA+, Seurat, GLUE, bindSC, Seurat CCA (Table S17-S20) [2].*

*Supplemental Fig S1 Gene expression inference from chromatin accessibility in human developing brain data [1] (A) Cell-wise Pearson correlation (y-axis) of inferred and measured chromatin accessibility by different methods (x-axis): CMOT (p=25%,50%,75%,100%), Seurat, MOFA+, GLUE, bindSC, Seurat CCA (Tables S1-S4).*

*Supplemental Methods*

*Benchmark against state-of-art for cross-modality inference*

*For the mouse brain dataset [22], which is an application of GLUE [21], we used their preprocessed dataset to benchmark all methods. We split this dataset into 80% train and 20% test, and benchmarked all methods on default parameters. For CMOT, we used the following parameters: K=5, d= 20, λ=1e03, η=1e-2, k=400, and used the top 10 features of scATAC-seq training data to find the k nearest neighbors. We induced our cell labels by hierarchical clustering of the cells in the training set and identified two clusters to regularize the optimal transport in CMOT. As shown in Fig S2, CMOT outperforms state-of-art methods with a median correlation of 0.76 compared to Seurat (median correlation=0.7, Wilcoxon p-value<3.4e-40), MOFA+(median correlation=0.74, Wilcoxon p-value<1.15e-05), GLUE(median correlation=0.72, Wilcoxon p-value<5.6e-25), bindSC (median correlation=0.72, Wilcoxon p-value<8e-19), and, Seurat CCA (median correlation=0.75, Wilcoxon p-value<1.9e-04). Even for partial correspondences, CMOT reports a higher performance (median r=0.76 for p=75%, median r=0.76 for p=50%) continuing to outperform other methods: Seurat (Wilcoxon p-value<1.25e-39), GLUE (Wilcoxon p-value<1.63e-24), MOFA+ (Wilcoxon p-value<1.67e-05), bindSC (Wilcoxon p-value<2.07e-158), Seurat CCA (Wilcoxon p-value<2.8e-4). Even for correspondence as low as 25%, CMOT performs comparably with median r=0.71, still outperforming Seurat (Wilcoxon p-value<0.07) (see Fig S2, Supplemental Methods, Supplemental Table S5-S8). We also compared CMOT with Polarbear on their mouse brain data for fair comparison using top 1000 features. We found that CMOT has significantly higher gene-wise correlations than Polarbear (515 genes versus 485 genes, Wilcoxon p-value<2.81e-02) and Polarbear co-assay (648 genes versus 352 genes, Wilcoxon p-value<1.2e-27), and, Seurat (1960 genes versus 101 genes, Wilcoxon p-value<2.97e-69) (Fig. S3, Tables S9-S10).*

*For the DEX-treated A549 dataset [2], which is a common application of CMOT and bindSC, we also split the dataset into 80% train and 20% test, similar to Fig 4. To run bindSC, we used the parameters suggested in their dataset tutorial [20]. We benchmarked other methods on tuned parameters on 80% of training data (Fig S9). After tuning, we used the following parameters for benchmarking: A) Seurat: k.nn=20, k.anchor=5, k.weight=100, k.filter=50; B) CMOT: latent dimension=25, top variable features=20, k-nearest neighbors=500, entropic regularization=100; C) GLUE: Dimensionality=30, Hidden layer depth=4, Hidden layer dimensionality=64, Dropout=0.3, Lambda graph=0.5; D) Seurat CCA: k.filter=50, k.weight=100, k.anchor=25; E) MOFA+: num_factors=100. As shown in supplemental Fig S7, CMOT outperforms state-of-art with a median correlation r of 0.52, compared to Seurat (median r=0.5, Wilcoxon p-value<21.27e-05), GLUE(median r=0.5, Wilcoxon p-value<4.7e-06), bindSC (median r=0.51, Wilcoxon p-value<0.016), Seurat CCA (median r=0.51, Wilcoxon p-value<0.016) and performs comparably to MOFA+(median r=0.52, Wilcoxon p-value<0.64). Even for partial correspondences, CMOT reports high and consistent performance (median r=0.52 for p=75%, median r=0.51 for p=50%) outperforming Seurat (Wilcoxon p-value<3.9e-05), GLUE (Wilcoxon p-value<1.6e-05), bindSC (Wilcoxon p-value<0.025), and, Seurat CCA (Wilcoxon p-value<0.029). (see Fig S7, Tables S17-S20).*

*To benchmark the human developing brain data [1], we split the dataset into 80% train and 20% as in Fig. 1. We trained all methods on default parameters and for CMOT, we used the same parameters as in Fig. 2. As shown Fig. S1, CMOT outperforms or performs comparably with state-of-arts with a median correlation of 0.67, compared to Seurat (median correlation=0.64, Wilcoxon p-value<1.23e-14), MOFA+(median correlation=0.41, Wilcoxon p-value=0), GLUE(median correlation=0.47, Wilcoxon p-value<1.4e-236), bindSC (median correlation=0.68, Wilcoxon p-value<0.97), and Seurat CCA (median correlation=0.68, Wilcoxon p-value<0.98). Also, for p<100%, CMOT continues to report consistent performance. For example, CMOT has significantly higher performances (median r=0.65 for p=75%, and r=0.63 for p=50%) than MOFA+ (Wilcoxon p-value<2.8e-294), GLUE (Wilcoxon p-value<1.3e-216), and Seurat (Wilcoxon p-value<3.43e-10). Also, with low correspondence such as p=25%, CMOT’s performance (r=0.61) is still significantly higher than MOFA+ (Wilcoxon p-value<1.65e-157) and GLUE (Wilcoxon p-value<2.4e-86) (see Fig S1, Tables S1-S4).*

A key limitation is the relatively simplistic benchmarking metrics used here: a correlation of gene expression. There are other metrics that should be utilized to evaluate the performance of multimodal cell matching. These include methods used in portions of the paper, such as FOSCTTM, silhouette score, or a confusion matrix for assessing accuracy.

We thank the reviewer for pointing this out. We evaluated CMOT’s inference performance using Pearson(and Spearman) correlation between inferred and true expression profiles (Fig 2A,3A,4A,5A S1A, S2A, S6A; Supplementary Tables S1-S4, S5-S8, S13, S16, S17-S20, S22-S25). Next, we used the suggested metrics to evaluate the 1) alignment step (step A) and 2) interpretability of CMOT’s inferences. For example, the FOSCTTM metric was used to assess alignments e.g. NMA, MMD-MA, SCOT, (see Supplemental Fig S12) and choose the appropriate alignment method. The silhouette score was used to identify how well CMOT’s inferred gene expression could separate the different cancer subtypes (see Fig 5B, C; Supplemental Table S26). Based on the FOSCTTM score, we chose Non-linear Manifold Alignment (NMA) to align partially corresponding multimodal datasets. However, users can choose the alignment method of their choice. Based on the silhouette score, we show that CMOT’s inferred gene expression can better separate the cancer clusters compared to other methods in the pan-cancer dataset. We incorporated this into the revision.

*(Supplemental Information)*

*Supplemental Fig S12 Boxplots show the FOSCTTM score for alignment methods: Nonlinear Manifold Alignment (NMA), Maximum mean discrepancy-based manifold alignment (MMD-MA) , Single-cell alignment with optimal transport (SCOT) and Unioncom on the four datasets. The latent dimensions are the optimal dimensions reported for each dataset i.e. d=20 for the human developing brain, d=15 PBMC, d=10 DEX-treated lung cancer data, and, d=10 pan-cancer data.*

*Results (Page 8)*

*Fig 5 Cross-modality inference between gene expression and chromatin accessibility can distinguish cancer types (B) Silhouette score (x-axis) across measured and inferred gene expressions (x-axis), and, measured chromatin peaks (Table S26)*

3. The training/validation data split varies significantly across the datasets used across the figures. These splits should be consistent/justified and also avoid overfitting the data. For instance

a. Fig 2 is 80% training/20% validation

b. Fig 3 is 6885 cells training/3994 validation

c. Fig 4 is 80% training/20% validation

d. Fig 5 is 60% training 40% validation

We thank the reviewer for pointing this out. We have split all datasets consistently using an 80-20 train-test split. In particular, for Fig 3, we split the 6885 cells in PBMC data (PBMC10k) into 80% train and 20% validation and show that CMOT achieves a median cell-wise Pearson correlation=0.83 for p=100% significantly outperforming MOFA+ (median correlation=0.8, Wilcoxon p-value<1.22e-05) and TotalVI (median correlation=-0.03, Wilcoxon p-value=0), and performing comparably to Seurat (Fig S4). For Fig 5, we also updated our Fig 5 based on pan-cancer data into 80% train and 20% test. CMOT outperforms (median correlation=0.67) both Seurat (median correlation=0.8, Wilcoxon p-value=0) and MOFA+ (median correlation=0.8, Wilcoxon p-value=0). We have also provided details of evaluations and results in the revision.

*(Excerpt for Pan-cancer data)*

*Results (Page 8 Line 180)*

*Finally, we tested CMOT to see how well it can infer between two modalities, especially for relevantly small datasets. We used a pan-cancer scCAT-seq dataset which jointly profiled 206 single-cell gene expression and chromatin accessibility on OCRs for three cancer cell lines: HCT116, HeLa-S3, and K562 [3]. We stratified split data into 80% training and 20% testing sets using cancer-type information.*

*Fig 5 Cross-modality inference between gene expression and chromatin accessibility can distinguish cancer types (A) Cell-wise Pearson correlation (y-axis) of inferred and measured gene expression by different methods (x-axis): CMOT(p=25%,50%,75%,100%),Seurat, MOFA+ (Table S22-S25) (B) Silhouette score (x-axis) across measured and inferred gene expressions (x-axis), and, measured chromatin peaks (Table S26) (C) PCA of inferred gene expression (D) Gene-wise correlation between the inferred and measured expression, comparing CMOT (y-axis) with MOFA+ and Seurat (x-axis). Dots: Genes; Numbers: Gene numbers above and below the dotted line (E) Peak-wise AUROC, comparing CMOT (y-axis) with MOFA+ and Seurat (x-axis). Dots: Peaks; Numbers: Peak numbers above and below the dotted line. P-values are calculated by a one-sided Wilcoxon rank-sum test.*

*(Excerpt for PBMC data)*

*Results (Page 5 Line 140)*

*Additionally, we also show benchmarking on PBMC10k, by splitting it into 80% training and 20% testing data (see Fig S4, Supplementary Methods, Tables S14-S16).*

*Supplemental Fig S4 Cross-modality inference between gene expression and chromatin accessibility in PBMC [5] (A) Cell-wise Pearson correlation (y-axis) of inferred and measured chromatin accessibility by different methods (x-axis): CMOT (p=25%,50%,75%,100%), Seurat, MOFA+, TotalVI (Table S14-S16) [26].*

*Supplemental Methods*

*Inferring protein expression from gene expression in Peripheral Blood Mononuclear Cells*

*We applied CMOT to infer protein expression from gene expression of peripheral blood mononuclear cells (PBMCs) using emerging CITE-seq data [5]. We randomly split the PBMC10k cells into 80% training for cross-validation and 20% testing set for evaluation. We trained CMOT with parameters: K=5, d= 15, λ=1e02, η = 1, k=100, and used the top 200 highly variable genes in the training data to find the k nearest neighbors. We induced cell labels by identifying two clusters using gene expression for the label regularization in optimal transport. As shown in Fig. S4, CMOT achieves a median correlation r of 0.91 for p=100% outperforming MOFA+ (median r=0.89, Wilcoxon p-values<1.22e-05) and TotalVI (median r=-0.08, Wilcoxon p-values=0) and performs comparably with Seurat (median r=0.92, Wilcoxon p-values<0.99). Also, for p=50%,75%, CMOT reports a higher correlation of 0.9 for both, outperforming both MOFA+ (Wilcoxon p-values<1.4e-03) and TotalVI (Wilcoxon p-values=0) (Tables S14-S16).*

How the custom parameter selection was made is unclear for CMOT (which varies across the data), whereas the benchmarked other methods used was default. How much of the difference in performance of CMOT versus others was a result of parameter tuning?

We thank the reviewer for pointing this out. We tested the inference performance of all methods for different hyperparameters on the DEX-treated A549 dataset as shown below (Fig S9). For CMOT, we tuned the hyperparameters: latent dimension, k-nearest neighbors, top variable features, and entropic regularization. For Seurat CCA, we tuned the hyperparameters k.anchors, k.filter, and, k.weight. For Seurat, we tuned the hyperparameters k.nn, k.anchors, k.filter, and, k.weight. For MOFA+, we followed the suggestions from the tutorial stating the use of default model training options unless you are familiar with the underlying data distribution. To avoid assumptions about the underlying data distributions, we set their likelihoods to default which is “gaussian”. However, we tuned the dataset for the number of latent factors (num_factors). For GLUE, we tested the dataset for 5 hyperparameters: Dimensionality, Hidden layer depth, Hidden layer dimensionality, Dropout, and, Lambda graph. Also for bindSC, we followed their tutorial for the same dataset (https://htmlpreview.github.io/?https://github.com/KChen-lab/bindSC/blob/master/vignettes/A549/A5 49.html) using the parameters suggested. Based on the parameter tuning, we chose the following parameters for the methods:

A. Seurat: k.nn=20, k.anchor=5, k.weight=100, k.filter=50

B. CMOT: latent dimension=25, top variable features=20, k-nearest neighbors=500, entropic regularization=100

C. GLUE: Dimensionality=30, Hidden layer depth=4, Hidden layer dimensionality=64, Dropout=0.3, Lambda graph=0.5

D. Seurat CCA: k.filter=50, k.weight=100, k.anchor=25

E. MOFA+: num_factors=100

*Supplemental Information*

*Supplemental Fig S9 Inference performance of all methods under different hyperparameter settings on DEX-treated A549 dataset [2]. (A) Seurat hyperparameters - k.nn: the number of multimodal neighbors to compute (default=20); k.anchor: How many neighbors (k) to use when finding anchors (default=5); k.weight: Number of neighbors to consider when weighting anchors (default=50); k.filter: How many neighbors (k) to use when filtering anchors (default=200); (B) CMOT hyperparameters – Latent dimension: latent dimension for alignment (step A); Top variable features: top high variables for k-nearest neighbors; k-nearest neighbors: number of neighbors for inference; Entropic regularization: entropic regularization parameter; (C) GLUE hyperparameters – Dimensionality: cell embedding dimensionality. Hidden layer depth: number of hidden layers in the data encoders and modality discriminator. Hidden layer dimensionality: dimensionality of hidden layers in the data encoders and modality discriminator. Dropout: dropout rate of hidden layers in data encoders and modality discriminator. Lambda graph: weight of the graph loss (D) Seurat CCA hyperparameters - k.anchor: How many neighbors (k) to use when finding anchors (default=5); k.weight: Number of neighbors to consider when weighting anchors (default=50) ; k.filter: How many neighbors (k) to use when filtering anchors (default=200); (E) MOFA+ hyperparameter - num_factors: latent factors*

The number of features used for the inference also varied greatly between datasets, eg

a. Fig 2: top 1000

b. Fig 3: top 200

c. Fig 4: top 20

d. Fig 5: top 150

e. How were these top parameters selected, and how much variation in performance across the methods do we see as a result of these?

We thank the reviewer for the comment. We do calculate the euclidean distance in the original space, however, we do not choose all features to do so. We choose only top n highly variable features to find the k nearest neighbors (knn). We propose to use n as a hyperparameter to select the n highly variable subset of features that can be used for the knn step. Below we show the robustness of CMOT’s performance for the different number of features selected. We also clarified this in the revision. In the plots below, we show CMOT’s inference performance (y-axis) as the number of features selected varies (x-axis) for different values of k (nearest neighbors). We select the optimal number of features n and k based on performance saturation i.e. the line plot stops increasing.

*Methods (Page 15 Line 456)*

*Parameter selection*

*In Step A, we found the optimal alignment by testing different values of common manifold dimensions and K nearest neighbors for building the similarity within each modality (Fig. S10). In Step B and Step C, we performed cross-validation to select the regularization coefficients for optimal transport, and, top highly variable features, and, k-nearest neighbors for modality inference. In particular, we chose the optimal number of features and k-nearest neighbors based on CMOT’s performance saturation (Fig. S11). Also, for all datasets, applied in this paper, we held out a 20% testing set to report CMOT’s performance. For datasets with no prior knowledge (e.g., cell types), we induced cell labels by cell clusters through hierarchical clustering of the training set, when training the final model (see Supplementary Methods). We split the training data into training and validation sets to select parameters through 5-fold cross-validation (see Supplementary Methods).*

*Supplemental Information*

*Supplemental Fig S11 Mean cell-wise Pearson correlation across top highly variable features for k nearest neighbors (A) Mean cell-wise Pearson correlation (y-axis) of inferred and measured gene expression by CMOT across a different number of top highly variable genes (x-axis) in human brain data [1]. (B) Mean cell-wise Pearson correlation (y-axis) of inferred and measured gene expression by CMOT across a different number of top highly variable genes (x-axis) in PBMC [5]. (C) Mean cell-wise Pearson correlation (y-axis) of inferred and measured gene expression by CMOT across a different number of top highly variable genes (x-axis) in A549 data [2]. (D) Mean cell-wise Pearson correlation (y-axis) of inferred and measured gene expression by CMOT across a different number of top highly variable genes (x-axis) in pan-cancer data [3].*

How much of the separation seen in cancer cell lines in Fig 5 can be done using only chromatin accessibility, compared to the inferred genes? I.E. is CMOT necessary for this separation

We thank the reviewer for pointing this out. We computed the silhouette score for chromatin peaks to see how well they could separate the cancer types. CMOT reports a high silhouette score (0.74), outperforming all the measured gene expression (0.25) and chromatin peaks (0.27), and, inferred gene expressions from MOFA+(-0.07) and Seurat (0.61) (see Supplemental Table S21).

*Results (Page 9 Line 197)*

*CMOT reports a high median silhouette score of 0.74 compared to the measured gene expression (0.25), measured chromatin peaks (0.27), and, inferred expressions from Seurat (0.61) and MOFA+ (-0.07) (Fig. 5B, Table S26). As shown in Fig. 5C, the cancer cells from three cancer cell lines can be separated using CMOT inferred gene expression, suggesting the capability of CMOT inference to reveal cancer-type-specific expression. Then, we evaluated the CMOT’s OCR peaks inference from gene expression.*

*Figure 5: Cross-modality inference between gene expression and chromatin accessibility can distinguish cancer types (B) Silhouette score (x-axis) across measured and inferred gene expressions (x-axis), and, measured chromatin peaks (Table S26)*

The authors mentioned other methods eg SCOT/MMD-MA can be used as alignment approaches. How much does the selection of alignment approaches affect CMOT inference performance?

We thank the reviewer for the suggestion. We tried other alignment methods SCOT, MMD-MA, and, Unioncom to the pan-cancer data. Below we show CMOT’s inference performance for latent dimensions d=5,10,15,20 (Fig S13). We can see that nonlinear manifold alignment (NMA) consistently outperforms other alignment methods. However, users can choose a method of their choice.

*Supplemental Information*

*Supplemental Fig S13 Benchmarking alignment methods (NMA, MMD-MA, SCOT, Unioncom) to compare CMOT’s inference in pan-cancer data [3] (A) Mean cell-wise Pearson correlation (y-axis) of inferred and measured gene expression by CMOT across alignment methods (x-axis) for d=5. (B) Mean cell-wise Pearson correlation (y-axis) of inferred and measured gene expression by CMOT across alignment methods (x-axis) for d=10. (C) Mean cell-wise Pearson correlation (y-axis) of inferred and measured gene expression by CMOT across alignment methods (x-axis) for d=15. (D) Mean cell-wise Pearson correlation (y-axis) of inferred and measured gene expression by CMOT across alignment methods (x-axis) for d=20.*

Line 99, missing words after "CMOT first aligns the [sic]."

Thanks. We have fixed the typo.

*Methods (Page 10 Line 272)*

*That is, if cells across modalities have none to partial correspondence between them, then CMOT first aligns them.*

Is there a rational for using Pearson and not a ranked based correlation approach for calculating cell-wise correlations in this study?

We thank the reviewer for pointing this out. We evaluated inferences using Spearman correlation, along with Pearson correlation. CMOT continues to outperform other methods or reports comparable performances with state-of-art.

For example, in the pan-cancer dataset, CMOT reports a higher median Spearman correlation of 0.71 (med. Pearson correlation=0.69) compared to most other methods:

a. Seurat (med. Spearman corr=0.67, med. Pearson corr=0.65),

b. MOFA+ (med. Spearman corr=0.5, med. Pearson corr=0.55),

(See Supplemental Tables S22-S25 for detailed results)

We also see a similar trend across other datasets (Supplemental Tables S1-S4, S13, S16, S17-S20).

*Supplemental Information*

*Supplemental Table S22 Gene expression inference from chromatin accessibility in pan-cancer data [3]. Mean and median cell-wise Pearson correlation between inferred and measured gene expression comparing methods (x-axis) CMOT (p=25%,50%,75%,100%), Seurat, MOFA+ plotted in Fig. 5A for pan-cancer data [3].*

*Supplemental Table S23 Gene expression inference from chromatin accessibility in pan-cancer data [3]. Mean and median cell-wise Spearman correlation between inferred and measured gene expression comparing methods (x-axis) CMOT (p=25%,50%,75%,100%), Seurat, MOFA+ for pan-cancer data [3].*

*Supplemental Table S24 Gene expression inference from chromatin accessiblity in pan-cancer data [3]. Wilcox-rank sum test p-values of cell-wise Pearson correlation between inferred and measured gene expression comparing methods (x-axis) CMOT (p=25%,50%,75%,100%), Seurat, and, MOFA+ plotted in Fig. 5A, Table S22 for pan-cancer cells [3]. All values have been calculated between CMOT variations (p=100%,75%,50%,25%) , and competing methods by setting alternative=“greater”. Supplemental Table S25 Gene expression inference from chromatin accessibility in pan-cancer data [3]. Wilcox-rank sum test p-values of cell-wise Spearman correlation between inferred and measured gene expression comparing methods (x-axis) CMOT (p=25%,50%,75%,100%), Seurat, and, MOFA+ plotted in Fig. 5A, Table S23 for pan-cancer cells [3]. All values have been calculated between CMOT variations (p=100%,75%,50%,25%), and competing methods by setting alternative=“greater”*

Median correlations are shown instead of mean. How different are the results in this case?

We thank the reviewer for the suggestion. We calculated the mean correlation values for all inferences. We observed that the performances across mean and median correlations remain consistent across all methods. We report Pearson correlation results below. Additionally, we also report mean and median Spearman correlation results in the supplement (Supplemental Tables S1, S5, S9, S13).

*Supplemental Information*

*Supplemental Table S1 Gene expression inference from chromatin accessibility in human developing brain data [1]. Mean and median cell-wise Pearson correlation between inferred and measured gene expression comparing methods (x-axis) CMOT(p=25%,50%,75%,100%), Seurat, MOFA+, GLUE, bindSC, and, Seurat CCA plotted in Fig. S6 for human developing brain [1].*

*Supplemental Table S17 Gene expression inference from chromatin accessibility in DEX-treat A549 lung cancer data [2]. Mean and median cell-wise Pearson correlation between inferred and measured gene expression comparing methods (x-axis) CMOT (p=25%,50%,75%,100%), Seurat, MOFA+, GLUE, bindSC, and, Seurat CCA plotted in Fig. S7 for DEX-treated A549 cells [2].*

*Supplemental Table S22 Gene expression inference from chromatin accessibility in pan-cancer data [3]. Mean and median cell-wise Pearson correlation between inferred and measured gene expression comparing methods (x-axis) CMOT (p=25%,50%,75%,100%), Seurat, MOFA+ plotted in Fig. 5A for pan-cancer data [3].*

The parameter mu for line 119 was set to 0.5. Is this a value that has been optimized or chosen for a particular reason?

We thank the reviewer for pointing this out. Although mu is a hyperparameter that can be tuned by the user, we chose to set mu=0.5 to give equal weightage to preserve both the local geometry of a modality and corresponding samples across modalities. This helps us eliminates the need for any assumptions about underlying data distributions of modalities. We have clarified this in the revised manuscript.

*Methods (Page 11 Line 300)*

*Here, we set \mu to 0.5. This allows equal importance to both preserving the local geometry of each modality as well as cell-to-cell correspondences, thereby eliminating the need for assuming underlying assumptions about data distributions of modalities.*

Line 274: Is it a typo for "top 8 highly predictive cells" or genes?

We thank the reviewer for the comment. The human brain dataset contains multiple cell types (e.g. IN, nIPC, etc), with a highly imbalanced number of cells within each type. Since the cell type with the smallest number of cells i.e. EC/Peric. had only 8 cells, we decided to pick the top 8 highly predictive cells from the other cell types for consistency. Then, we used these cells to calculate the AUPRC of marker genes within each type. A higher AUPRC score of a marker gene within a cell type suggests that CMOT’s inferred expression is able to distinguish cell types. We have further clarified this in the revised manuscript.

*Results (Page 5 Line 115)*

*Next, we evaluated if the CMOT inferred gene expression to classify brain cell types. We used known cell-type marker genes provided along with the dataset and selected the top 8 highly predictive cells from each cell type within our inferred gene expression. Due to the high imbalance of the number of cells within each cell type, we picked the number of cells based on the size of the smallest cell type. In this case, EC/Peric. had only 8 cells, therefore we picked only 8 cells from the other cell types.*

Why was Seurat & MOFA not used for the inference in the Mouse Brain data for consistency, where CMOT was compared with Polarbear?

We thank the reviewer for this comment. As suggested, we compared the mouse brain data with MOFA+ and Seurat. CMOT reports median correlation of 0.71 comparable to Polarbear (med. correlation=0.71,Wilcoxon p-value<0.7), Polarbear co-assay (med. correlation=0.71,Wilcoxon p-value<0.29), Seurat (med. correlation=0.63,Wilcoxon p-value<2.97e-69), and, MOFA+ (med. correlation=0.71,Wilcoxon p-value<0.7). We found that CMOT has significantly higher gene-wise correlations than both Polarbear (515 genes versus 485 genes, Wilcoxon p-value<2.81e-02) and Polarbear co-assay (648 genes versus 352 genes, Wilcoxon p-value<1.2e-27). and Polarbear co-assay) (Fig. S3).

*Supplemental Information*

*Supplemental Fig S3 Gene expression inference from chromatin accessibility in mouse brain data [22] (A) Cell-wise Pearson correlation (y-axis) of inferred and measured gene expression by different methods (x-axis): CMOT(p=25%,50%,75%,100%), Seurat, MOFA+, Polarbear, and, Polarbear-coassay (Table S27-S28) (B) Gene-wise correlation between the inferred and measured expression, comparing CMOT (y-axis) with Polarbear, Polarbear-coassay, MOFA+, and, Seurat (x-axis). Dots: Genes; Numbers: Gene numbers above and below the dotted line.*

*Supplemental Methods*

*Benchmark against state-of-art for cross-modality inference*

*We also compared CMOT with Polarbear on their mouse brain data for fair comparison using top 1000 features. We found that CMOT has significantly higher gene-wise correlations than both Polarbear (515 genes versus 485 genes, Wilcoxon p-value<2.81e-02) and Polarbear co-assay (648 genes versus 352 genes, Wilcoxon p-value<1.2e-27), and, Seurat (1960 genes versus 101 genes, Wilcoxon p-value<2.97e-69) (Fig. S3, Tables S9-S10).*

Violin plots are much preferred over boxplots eg in Fig 3A due to their ability to display distribution Better

We thank the reviewer for the suggestion. We have updated all our boxplots to violin plots.

*Results*

*(Page 4)*

*Fig 2 Single-cell gene expression inference from chromatin accessibility in developing human brain (A) Cell-wise Pearson correlation (y-axis) of inferred and measured gene expression by different methods (x-axis): CMOT (p=25%,50%,75%,100%), Seurat, MOFA+ (Table S1-S4). See Fig. S1 A and Supplementary Methods for additional benchmarking.*

*(Page 6)*

*Fig 3 Inferring protein expression from gene expression in single-cell peripheral blood mononuclear cells (A) Cell-wise Pearson correlation (y-axis) of inferred and measured protein expression by different methods (x-axis): CMOT(p=25%,50%,75%,100%), Seurat, MOFA+, TotalVI (Tables S11-S12)*

*Fig 4 Inference of gene expression for drug-treated lung cancer cells using chromatin accessibility (A) Cell-wise Pearson correlation (y-axis) of inferred and measured gene expression by different methods (x-axis): CMOT(p=25%,50%,75%,100%),Seurat, MOFA+ (Table S17-S20). See Fig. S7 A and Supplementary Methods for additional benchmarking.*

*(Page 8)*

*Fig 5 Cross-modality inference between gene expression and chromatin accessibility can distinguish cancer types (A) Cell-wise Pearson correlation (y-axis) of inferred and measured gene expression by different methods (x-axis): CMOT(p=25%,50%,75%,100%),Seurat, MOFA+ (Table S24-S25)*

The number of cells used for Fig 5 was not specified, since this number was detailed for the other datasets, it will be good for consistency

We thank the reviewer for pointing this out. We updated the manuscript with details about the pan-cancer dataset.

*Results (Page 8 Line 180)*

*Finally, we tested CMOT to see how well it can infer between two modalities, especially for relevantly small datasets. We used a pan-cancer scCAT-seq dataset which jointly profiled 206 single-cell gene expression and chromatin accessibility on OCRs for three cancer cell lines: HCT116, HeLa-S3, and K562 [3].*

I noticed the correlation by all methods were consistently lower compared to other figures. Does this mean this data is of lower quality or perhaps is a more challenging dataset to predict? The authors should comment on this for readers.

We thank the reviewer for this insightful comment. We attribute the variation in correlation across datasets to factors like the number of available cells, total features used, and, the sparsity of the datasets used in training. We noticed a higher correlation for datasets either with more training cells or a high number of features. However, data sparsity continues to be a challenge with single-cell profiling technologies and therefore affects inference performance for such datasets.

*Discussion (Page 10 Line 230)*

*Additionally, we see that the overall correlation scores of all methods consistently varies across datasets. We attribute this variation in correlation across datasets to factors like the number of available cells, total features used, and, the sparsity of the datasets used in training. We noticed a higher correlation for datasets either with more training cells or a high number of features. However, data sparsity continues to be a challenge with single-cell profiling technologies and therefore affects inference performance for such datasets.*

**2^nd^ round**

**Reviewer 1**

The authors have addressed most of my previous comments, and I am inclined to recommend acceptance of the manuscript. However, I still have a few follow-up comments that could further strengthen the authors' argument that the limitations raised in the last round have been well-addressed.

In this revision, the authors implemented different levels of dropouts (e.g., p=25%, ..., p=100%), where p=100% represents no cell correspondence across modalities. Even in this scenario, the method performed very well when compared to other state-of-the-art methods, which is excellent evidence. However, the single-cell multi-omics data that the authors used are still joint profiling that simultaneously measures multiple modalities. While I understand why the authors chose to do it this way (as the true cell correspondence information is available for validation and downstream analysis), it would be more convincing to demonstrate the method on single-cell multi-omics data without joint profiling (e.g., single-cell RNA-seq and single-cell ATAC-seq measurements on the same tissue but not the same set of cells). Ideally, the method should still be applicable to such a dataset to generate cells across modalities. After that, a comparison between the generated cells and the actual measurements can be evaluated (even when no actual correspondence is known, e.g., by examining the overall data distribution or the overlap of cells in the reduced latent space). Since the simultaneous profiling of multiple modalities of single cells is a challenging problem that the method aims to address, it is crucial to test and evaluate the method under these circumstances to demonstrate its usefulness in various application scenarios where joint single-cell measurements may not be available.

It is exciting to see that the authors have added another method module to address the issue of the "kNN" force-mapping problem. The proposed strategy is solid, and the authors have provided an example demonstration. Here, I have a follow-up question. Given that the author's strategy is to remove outliers that do not have good matching across modalities, in a scenario where most cells in one modality (e.g., RNA-seq) do not have good correspondence in another (e.g., ATAC-seq), will the method report confidence or provide a warning to inform users that their cross-modal inference might be problematic because the two or more modalities are too distinct?

**Reviewer 2**

The authors have addressed all my concerns. Congratulations on a wonderful contribution.

**Authors’ response**

Reviewer #1

In this revision, the authors implemented different levels of dropouts (e.g., p=25%, ..., p=100%), where p=100% represents no cell correspondence across modalities. Even in this scenario, the method performed very well when compared to other state-of-the-art methods, which is excellent evidence. However, the single-cell multi-omics data that the authors used are still joint profiling that simultaneously measures multiple modalities. While I understand why the authors chose to do it this way (as the true cell correspondence information is available for validation and downstream analysis), it would be more convincing to demonstrate the method on single-cell multi-omics data without joint profiling (e.g., single-cell RNA-seq and single-cell ATAC-seq measurements on the same tissue but not the same set of cells). Ideally, the method should still be applicable to such a dataset to generate cells across modalities. After that, a comparison between the generated cells and the actual measurements can be evaluated (even when no actual correspondence is known, e.g., by examining the overall data distribution or the overlap of cells in the reduced latent space). Since the simultaneous profiling of multiple modalities of single cells is a challenging problem that the method aims to address, it is crucial to test and evaluate the method under these circumstances to demonstrate its usefulness in various application scenarios where joint single-cell measurements may not be available.

We thank the reviewer for this suggestion. We tested CMOT on single profiled scRNA-seq and scATAC-seq datasets from the developing human brain (week 21) [1]. We chose five major cell types common to each dataset: Glutamatergic neuron, Late Radial Glia, neuronal Intermediate Progenitor cells, Oligodendrocyte Progenitor Cell\Oligodendrocyte, and Pericyte. We had 7420 cells from scATAC-seq and 6542 cells from scRNA-seq. We split each dataset using an 80-20 train-test split. We tested all methods on 1000 and 2000 most variable features. We evaluated the inferred expressions using the Jensen-Shannon distance (JSD) (Fig. S14). In particular, we computed the JSD between inferred and measured gene expression of cells from the five major cell types. This allows us to see how well each method preserves the individual cell-type distributions. CMOT outperforms the state of-art methods by reporting the lowest JSD across all cell types. We have also added the results to our revised manuscript.

[1] A. E. Trevino et al., “Chromatin and gene-regulatory dynamics of the developing human cerebral cortex at single-cell resolution,” Cell, vol. 184, no. 19, pp. 5053-5069.e23, Sep. 2021, doi: 10.1016/j.cell.2021.07.039.

It is exciting to see that the authors have added another method module to address the issue of the "kNN" force-mapping problem. The proposed strategy is solid, and the authors have provided an example demonstration. Here, I have a follow-up question. Given that the author's strategy is to remove outliers that do not have good matching across modalities, in a scenario where most cells in one modality (e.g., RNA-seq) do not have good correspondence in another (e.g., ATAC-seq), will the method report confidence or provide a warning to inform users that their cross-modal inference might be problematic because the two or more modalities are too distinct?

We thank the reviewer for pointing this out. We have added an additional warning message that informs users about the poorly mapped cells in the test single modality that the users may want to remove to avoid incorrect inferences. We have updated this point in the revised manuscript and the source code.

**3^rd^ round**

**Reviewer 1**

The authors have addressed my last major concern, I now endorse the publication of this manuscript. Congrats on the great work!
